# Brain Expression Genome-Wide Association Study (eGWAS) Identifies Human Disease-Associated Variants

**DOI:** 10.1371/journal.pgen.1002707

**Published:** 2012-06-07

**Authors:** Fanggeng Zou, High Seng Chai, Curtis S. Younkin, Mariet Allen, Julia Crook, V. Shane Pankratz, Minerva M. Carrasquillo, Christopher N. Rowley, Asha A. Nair, Sumit Middha, Sooraj Maharjan, Thuy Nguyen, Li Ma, Kimberly G. Malphrus, Ryan Palusak, Sarah Lincoln, Gina Bisceglio, Constantin Georgescu, Naomi Kouri, Christopher P. Kolbert, Jin Jen, Jonathan L. Haines, Richard Mayeux, Margaret A. Pericak-Vance, Lindsay A. Farrer, Gerard D. Schellenberg, Alzheimer's Disease Genetics Consortium, Ronald C. Petersen, Neill R. Graff-Radford, Dennis W. Dickson, Steven G. Younkin, Nilüfer Ertekin-Taner

**Affiliations:** 1 Department of Neuroscience, Mayo Clinic, Jacksonville, Florida, United States of America; 2 Department of Biostatistics, Mayo Clinic, Rochester, Minnesota, United States of America; 3 Department of Biostatistics, Mayo Clinic, Jacksonville, Florida, United States of America; 4 Microarray Core, Mayo Clinic, Rochester, Minnesota, United States of America; 5 Department of Molecular Physiology and Biophysics and Vanderbilt Center for Human Genetics Research, Vanderbilt University, Nashville, Tennessee, United States of America; 6 Gertrude H. Sergievsky Center, Department of Neurology, and Taub Institute on Alzheimer's Disease and the Aging Brain, Columbia University, New York, New York, United States of America; 7 The John P. Hussman Institute for Human Genomics and Dr. John T. Macdonald Foundation Department of Human Genetics, University of Miami, Miami, Florida, United States of America; 8 Departments of Biostatistics, Medicine (Genetics Program), Ophthalmology, Neurology, and Epidemiology, Boston University, Boston, Massachusetts, United States of America; 9 Department of Pathology and Laboratory Medicine, University of Pennsylvania Perelman School of Medicine, Philadelphia, Pennsylvania, United States of America; 10 Department of Neurology, Mayo Clinic, Rochester, Minnesota, United States of America; 11 Department of Neurology, Mayo Clinic, Jacksonville, Florida, United States of America; Georgia Institute of Technology, United States of America

## Abstract

Genetic variants that modify brain gene expression may also influence risk for human diseases. We measured expression levels of 24,526 transcripts in brain samples from the cerebellum and temporal cortex of autopsied subjects with Alzheimer's disease (AD, cerebellar n = 197, temporal cortex n = 202) and with other brain pathologies (non–AD, cerebellar n = 177, temporal cortex n = 197). We conducted an expression genome-wide association study (eGWAS) using 213,528 *cis*SNPs within ±100 kb of the tested transcripts. We identified 2,980 cerebellar *cis*SNP/transcript level associations (2,596 unique *cis*SNPs) significant in both ADs and non–ADs (q<0.05, p = 7.70×10^−5^–1.67×10^−82^). Of these, 2,089 were also significant in the temporal cortex (p = 1.85×10^−5^–1.70×10^−141^). The top cerebellar *cis*SNPs had 2.4-fold enrichment for human disease-associated variants (p<10^−6^). We identified novel *cis*SNP/transcript associations for human disease-associated variants, including progressive supranuclear palsy *SLCO1A2*/rs11568563, Parkinson's disease (PD) *MMRN1*/rs6532197, Paget's disease *OPTN*/rs1561570; and we confirmed others, including PD *MAPT*/rs242557, systemic lupus erythematosus and ulcerative colitis *IRF5*/rs4728142, and type 1 diabetes mellitus *RPS26*/rs1701704. In our eGWAS, there was 2.9–3.3 fold enrichment (p<10^−6^) of significant *cis*SNPs with suggestive AD–risk association (p<10^−3^) in the Alzheimer's Disease Genetics Consortium GWAS. These results demonstrate the significant contributions of genetic factors to human brain gene expression, which are reliably detected across different brain regions and pathologies. The significant enrichment of brain *cis*SNPs among disease-associated variants advocates gene expression changes as a mechanism for many central nervous system (CNS) and non–CNS diseases. Combined assessment of expression and disease GWAS may provide complementary information in discovery of human disease variants with functional implications. Our findings have implications for the design and interpretation of eGWAS in general and the use of brain expression quantitative trait loci in the study of human disease genetics.

## Author Summary

Genetic variants that regulate gene expression levels can also influence human disease risk. Discovery of genomic loci that alter brain gene expression levels (brain expression quantitative trait loci = eQTLs) can be instrumental in the identification of genetic risk underlying both central nervous system (CNS) and non–CNS diseases. To systematically assess the role of brain eQTLs in human disease and to evaluate the influence of brain region and pathology in eQTL mapping, we performed an expression genome-wide association study (eGWAS) in 773 brain samples from the cerebellum and temporal cortex of ∼200 autopsied subjects with Alzheimer's disease (AD) and ∼200 with other brain pathologies (non–AD). We identified ∼3,000 significant associations between *cis*SNPs near ∼700 genes and their cerebellar transcript levels, which replicate in ADs and non–ADs. More than 2,000 of these associations were reproducible in the temporal cortex. The top *cis*SNPs are enriched for both CNS and non–CNS disease-associated variants. We identified novel and confirmed previous *cis*SNP/transcript associations for many disease loci, suggesting gene expression regulation as their mechanism of action. These findings demonstrate the reproducibility of the eQTL approach across different brain regions and pathologies, and advocate the combined use of gene expression and disease GWAS for identification and functional characterization of human disease-associated variants.

## Introduction

Expression quantitative trait loci (eQTL) are genomic loci that influence levels of gene transcripts and can be mapped by genetic linkage in families or eGWAS in unrelated populations [1]. eQTLs are distinct from other complex trait loci, because they directly identify the target gene, since the transcript trait is a reflection of the mRNA level from a single gene. Furthermore, eQTLs imply regulation of gene expression as the mechanism of action for the underlying variants. Recently, few studies identified an enrichment of eQTLs from lymphocytes [2] and lymphoblasts [3] amongst human complex disease and trait GWAS loci, suggesting that eQTLs may be useful in mapping human disease-associated variants.

Most human eQTL mapping studies to date assessed immortalized lymphoblastoid cell lines [4], [5], [6], [7], [8], [9], [10], [11], [12] and family-based samples from the CEPH [4], [5], [6], [7], [8], [13] (Centre d'Etude du polymorphisme humain) or HapMap [10], [11], [14], [15] repositories. Multiple other small and large scale eQTL studies investigated other tissues and populations including lymphocytes [16], monocytes [17], T-cells [18], fibroblasts [18], skin [19], subcutaneous and omental adipose tissue [20], [21], bone [22], liver [23] and brain [24], [25].

Despite the assumption that brain eQTLs would also influence human diseases and traits, there are no systematic gene mapping studies for human diseases that utilize brain gene expression phenotypes. Furthermore, the brain region most relevant for such studies and the influence of brain pathology on eQTL mapping studies are largely unknown. To address these issues, we performed an eQTL using cerebellar tissue from 197 subjects with Alzheimer's disease (AD) neuropathology and 177 with other pathologies (non–AD). We validated the results in a different brain region using temporal cortex samples from 202 ADs and 197 non–ADs (Supplementary Tables 1 and 2 in Dataset S1), 85% of whom overlapped with the cerebellar group. We evaluated significant *cis*SNPs from our study for association with human diseases/traits using a GWAS catalog [26]. We also assessed our significant eGWAS *cis*SNPs for association with two central nervous system (CNS) diseases, progressive supranuclear palsy (PSP) [27] and AD risk [28], using two recent GWAS for these diseases.

Our results demonstrate the power of the brain eQTL approach in the identification and characterization of many human CNS and non–CNS disease-associated variants. This study also highlights the remarkable reproducibility of human eQTLs across different brain regions and pathologies, which has implications for the design of eGWAS in general. Combined assessment of eQTLs and disease risk loci can be instrumental in mapping disease genes with regulatory variants.

## Results

### Brain eGWAS

Levels of 24,526 transcripts for 18,401 genes were measured in 773 brain samples from the cerebellum and temporal cortex of ∼200 ADs and ∼200 non–ADs, using WG-DASL assays. Nearly 70% of all probes could be detected in >75% of the samples tested. All autopsied subjects were genotyped for 313,330 single nucleotide polymorphisms (SNPs) from Illumina HumanHap300-Duo Genotyping BeadChips, as part of the Mayo AD GWAS [29]. An eGWAS testing association of transcript levels with *cis*SNPs was performed using multivariable linear regression correcting for *APOE* ε4 dosage, age at death, gender and multiple technical variables. False discovery rate (FDR)-based q values [30] (q) were used for corrections of multiple testing.

**Figure 1:**
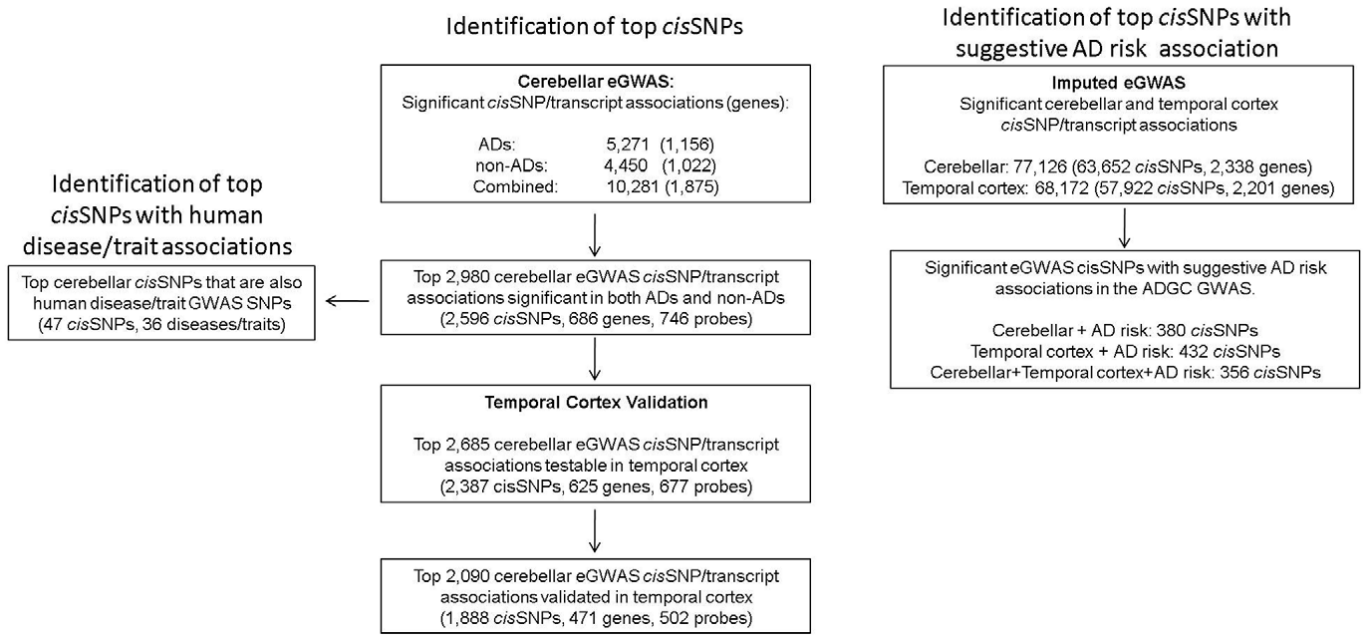
Summary of brain eGWAS and human disease associations.

To achieve internal replication, we first analyzed the ADs and non–ADs separately. In our cerebellar eGWAS, at q<0.05, there were 5,271 significant *cis*SNP/transcript associations (1,156 unique genes) in the AD, 4,450 (1,022 unique genes) in the non–AD and 10,281 (1,875 unique genes) in the combined datasets. Q-Q plots suggested a clear excess of significant results (Figure 1, Figure S1a–S1d). 2,980 *cis*SNP/transcript associations (2,596 unique *cis*SNPs, 686 unique genes) were significant at q<0.05 in both ADs and non–ADs (Table 1, Supplementary Table 3 in Dataset S1, Figure S2). The direction and magnitude of associations in both groups demonstrate remarkable similarities (Pearson's correlation coefficient = 0.98, p<0.0001). The box plots depicted for some of these top associations (Figure S3a–S3c) demonstrate this replication in ADs and non–ADs. Most associations have an additive or dominant pattern with respect to the minor allele.

**Table 1:** Examples of top cerebellar eGWAS *cis*SNP/transcript associations.

				NON–AD				AD				ALL			
CHR	SNP	PROBE	SYMBOL	P	P_Bonf_	Q	Beta	P	P_Bonf_	Q	Beta	P	P_Bonf_	Q	Beta
11	rs11552421	ILMN_1651745	TMEM25	1.44.E-68	6.37.E-63	1.14.E-58	2.69	1.67.E-82	7.41.E-77	7.26.E-71	2.65	3.50.E-153	1.55.E-147	3.10.E-138	2.67
5	rs3776455	ILMN_1718932	MTRR	6.70.E-64	2.97.E-58	1.90.E-54	1.30	4.06.E-70	1.80.E-64	7.41.E-60	1.12	6.37.E-133	2.83.E-127	7.34.E-120	1.20
6	rs1096699	ILMN_1694711	MAD2L1BP	6.09.E-56	2.70.E-50	4.86.E-47	1.31	6.54.E-60	2.90.E-54	5.35.E-50	1.22	1.41.E-116	6.25.E-111	5.44.E-104	1.26
12	rs10843881	ILMN_2345908	DDX11	7.87.E-54	3.49.E-48	3.15.E-45	-1.06	5.59.E-58	2.48.E-52	9.92.E-49	−1.06	1.48.E-112	6.57.E-107	2.39.E-100	−1.06
12	rs2708389	ILMN_1730477	TAS2R43	9.30.E-59	4.12.E-53	1.29.E-49	1.13	5.26.E-53	2.34.E-47	2.23.E-44	1.01	2.80.E-112	1.24.E-106	2.39.E-100	1.07
12	rs1971762	ILMN_1807798	ATP5G2	1.52.E-44	6.73.E-39	4.49.E-37	0.96	4.76.E-59	2.11.E-53	2.11.E-49	1.04	1.10.E-104	4.87.E-99	1.64.E-93	1.01
21	rs2838859	ILMN_2376667	POFUT2	1.19.E-48	5.28.E-43	8.06.E-41	−0.81	1.17.E-53	5.20.E-48	6.24.E-45	−0.89	2.86.E-103	1.27.E-97	2.73.E-92	−0.86
8	rs3802266	ILMN_2184966	ZHX2	8.18.E-44	3.63.E-38	2.17.E-36	−1.89	2.13.E-56	9.47.E-51	2.64.E-47	−1.99	4.35.E-101	1.93.E-95	3.06.E-90	−1.94
19	rs260462	ILMN_2052079	ZNF544	7.19.E-54	3.19.E-48	3.15.E-45	−1.21	1.75.E-47	7.79.E-42	2.60.E-39	−1.12	8.26.E-101	3.67.E-95	4.38.E-90	−1.16
17	rs2525574	ILMN_2369018	EVI2A	2.04.E-48	9.05.E-43	1.08.E-40	1.81	8.82.E-49	3.92.E-43	1.61.E-40	1.79	1.52.E-99	6.73.E-94	4.88.E-89	1.81
7	rs6955367	ILMN_1723984	PILRB	1.58.E-52	6.99.E-47	4.02.E-44	1.21	8.75.E-49	3.89.E-43	1.66.E-40	0.99	4.80.E-97	2.13.E-91	1.23.E-86	1.08
7	rs7313	ILMN_2400759	CPVL	2.40.E-42	1.06.E-36	5.15.E-35	−0.88	2.13.E-49	9.48.E-44	5.07.E-41	−1.01	5.00.E-92	2.22.E-86	5.96.E-82	−0.96
11	rs7124057	ILMN_2262288	EEF1G	6.28.E-50	2.78.E-44	6.10.E-42	−1.03	1.54.E-41	6.83.E-36	4.95.E-34	−0.74	2.64.E-91	1.17.E-85	2.67.E-81	−0.90
10	rs2182513	ILMN_1689177	PPAPDC1A	4.60.E-35	2.04.E-29	2.09.E-28	0.87	1.25.E-52	5.55.E-47	4.30.E-44	0.82	1.08.E-87	4.79.E-82	6.93.E-78	0.84
6	rs2975033	ILMN_2130441	HLA-H	8.19.E-42	3.63.E-36	1.60.E-34	1.53	8.13.E-42	3.61.E-36	2.83.E-34	1.39	3.53.E-84	1.57.E-78	1.73.E-74	1.45
5	rs11738432	ILMN_2093720	THG1L	5.52.E-45	2.45.E-39	1.84.E-37	−0.94	3.78.E-39	1.68.E-33	9.18.E-32	−0.81	8.06.E-84	3.58.E-78	3.49.E-74	−0.86
15	rs12912744	ILMN_1691772	ZSCAN29	1.25.E-35	5.53.E-30	7.32.E-29	−0.52	4.47.E-47	1.99.E-41	5.78.E-39	−0.59	3.47.E-83	1.54.E-77	1.33.E-73	−0.56
22	rs136564	ILMN_1809147	FAM118A	1.79.E-35	7.93.E-30	8.98.E-29	0.88	1.58.E-44	7.02.E-39	7.45.E-37	0.82	8.44.E-81	3.74.E-75	2.57.E-71	0.85
17	rs8070454	ILMN_2388272	MED24	1.77.E-33	7.82.E-28	5.89.E-27	−1.98	7.10.E-46	3.15.E-40	7.11.E-38	−2.05	3.51.E-80	1.56.E-74	9.58.E-71	−2.03
20	rs2387976	ILMN_2064132	NANP	3.88.E-38	1.72.E-32	3.69.E-31	−0.57	1.42.E-40	6.29.E-35	3.68.E-33	−0.51	9.58.E-79	4.25.E-73	1.93.E-69	−0.54
7	rs10239340	ILMN_2312606	IRF5	5.84.E-33	2.59.E-27	1.66.E-26	0.81	7.47.E-44	3.32.E-38	3.05.E-36	0.82	1.52.E-76	6.73.E-71	2.31.E-67	0.81
6	rs2395943	ILMN_1683279	PEX6	7.06.E-35	3.13.E-29	2.93.E-28	−0.39	1.90.E-41	8.43.E-36	5.68.E-34	−0.40	3.63.E-76	1.61.E-70	5.06.E-67	−0.39
13	rs1046028	ILMN_2390162	PHF11	1.08.E-34	4.79.E-29	4.28.E-28	0.37	1.26.E-40	5.61.E-35	3.52.E-33	0.33	1.07.E-75	4.73.E-70	1.36.E-66	0.35
17	rs1981997	ILMN_1710903	MAPT	1.89.E-37	8.38.E-32	1.48.E-30	−0.51	2.55.E-33	1.13.E-27	1.20.E-26	−0.44	4.16.E-71	1.85.E-65	3.88.E-62	−0.48
9	rs2240913	ILMN_1811048	GPR107	4.16.E-34	1.84.E-28	1.51.E-27	0.62	3.74.E-36	1.66.E-30	4.81.E-29	0.69	2.76.E-69	1.23.E-63	2.39.E-60	0.66
16	rs11649236	ILMN_2201966	N4BP1	1.99.E-39	8.82.E-34	2.59.E-32	−1.70	2.71.E-32	1.20.E-26	1.10.E-25	−1.48	5.69.E-69	2.53.E-63	4.37.E-60	−1.57
10	rs7909832	ILMN_1795336	PTER	3.28.E-33	1.45.E-27	1.01.E-26	−0.70	6.43.E-35	2.86.E-29	5.23.E-28	−0.81	1.70.E-68	7.54.E-63	1.04.E-59	−0.75
5	rs11134054	ILMN_1789419	EXOC3	1.45.E-35	6.43.E-30	8.07.E-29	−0.54	1.07.E-33	4.77.E-28	5.40.E-27	−0.59	4.40.E-68	1.95.E-62	2.52.E-59	−0.57
19	rs10405576	ILMN_2074477	GPR4	2.49.E-32	1.10.E-26	6.57.E-26	−1.35	4.86.E-34	2.16.E-28	3.04.E-27	−1.27	5.76.E-68	2.56.E-62	3.09.E-59	−1.32
6	rs198834	ILMN_2075334	HIST1H4C	1.98.E-29	8.78.E-24	2.23.E-23	−0.42	8.82.E-38	3.92.E-32	1.60.E-30	−0.44	4.76.E-67	2.11.E-61	2.05.E-58	−0.43
21	rs6517526	ILMN_2296011	BRWD1	3.00.E-30	1.33.E-24	4.50.E-24	−0.71	6.10.E-37	2.71.E-31	9.69.E-30	−0.88	6.31.E-67	2.80.E-61	2.51.E-58	−0.80
22	rs737950	ILMN_1697286	SF3A1	2.77.E-30	1.23.E-24	4.36.E-24	0.42	3.78.E-34	1.68.E-28	2.52.E-27	0.45	2.05.E-66	9.11.E-61	7.72.E-58	0.44
1	rs1763601	ILMN_2364072	CLCNKA	1.06.E-36	4.69.E-31	7.35.E-30	1.30	8.25.E-31	3.66.E-25	1.99.E-24	1.14	3.49.E-66	1.55.E-60	1.24.E-57	1.22
12	rs10878255	ILMN_2183938	LEMD3	1.25.E-29	5.54.E-24	1.55.E-23	−0.68	9.20.E-36	4.09.E-30	9.31.E-29	−0.56	2.61.E-65	1.16.E-59	7.91.E-57	−0.62
19	rs4806187	ILMN_1655637	UPK1A	6.89.E-27	3.05.E-21	4.54.E-21	−0.99	5.73.E-38	2.55.E-32	1.15.E-30	−1.05	2.82.E-65	1.25.E-59	8.11.E-57	−1.04
11	rs2848630	ILMN_1765332	TIMM10	4.66.E-36	2.07.E-30	3.05.E-29	−0.62	1.18.E-28	5.23.E-23	1.84.E-22	−0.42	3.40.E-65	1.51.E-59	9.32.E-57	−0.53
17	rs962800	ILMN_2401641	ALDH3A2	7.84.E-28	3.48.E-22	6.56.E-22	0.44	4.61.E-38	2.05.E-32	9.84.E-31	0.49	3.31.E-64	1.47.E-58	8.21.E-56	0.46

There are 2,980 cerebellar *cis*SNP/transcript associations with q<0.05 both in the ADs and non–ADs. Some of these top associations are shown. Only one *cis*SNP/transcript pair is selected for depiction. The chromosome (CHR), SNP, Probe, Gene Symbol (SYMBOL) of these associations are depicted. The uncorrected (P), Bonferroni-corrected (P_Bonf_) P, q values, and Beta coefficients of association are shown for the Non–ADs, ADs and combined (All) analyses. Regression coefficients are based on the SNP minor allele using an additive model.

To assess the genetic component contributing to gene expression variability, we estimated intraclass correlation coefficients (ICC) [31] in the 15 samples measured in replicate on 5–6 different plates and 2–3 different days. Between-subject variance accounted for a median of 60% of total probe expression variance (Supplementary Table 4 in Dataset S1; Figure S4). The 746 probes for the top 2,980 cerebellar *cis*SNP associations had higher between-subject variance (median = 78%).

Using multivariable linear regression, we next estimated the percent variation in cerebellar probe expression levels due to the “best” *cis*SNP for each transcript after accounting for technical and biological covariates. We found that the “best” *cis*SNP explained a median of ∼3% of the expression variation. For the top 746 probes, the “best” *cis*SNPs accounted for a median of 18% of the expression variance (Table 2, Supplementary Table 5 in Dataset S1).

**Table 2:** Variance of cerebellar probe expression levels due to technical, biological, and *cis*SNP effects.

Probe	Symbol	Raw_Variance	R2technical	addR2covariates	adjR2covariates	addR2best-SNP	adjR2best-SNP	Best-cis-SNP
ILMN_1651745	TMEM25	1.894	0.08	0.004	0.005	0.784	0.852	rs11552421
ILMN_1718932	MTRR	0.864	0.05	0.004	0.005	0.768	0.809	rs3776455
ILMN_1694711	MAD2L1BP	1.169	0.154	0.007	0.008	0.642	0.763	rs1096699
ILMN_1730477	TAS2R43	1.212	0.372	0.001	0.002	0.477	0.76	rs2708389
ILMN_2345908	DDX11	0.798	0.029	0.003	0.003	0.732	0.755	rs4031375
ILMN_1807798	ATP5G2	0.722	0.156	0.009	0.011	0.61	0.722	rs1971762
ILMN_2052079	ZNF544	0.983	0.055	0.003	0.003	0.675	0.714	rs260462
ILMN_2369018	EVI2A	2.208	0.043	0.005	0.005	0.68	0.711	rs2525574
ILMN_2184966	ZHX2	2.816	0.11	0.012	0.013	0.629	0.706	rs3802266
ILMN_2376667	POFUT2	0.503	0.076	0.032	0.035	0.649	0.703	rs2838859
ILMN_1723984	PILRB	0.671	0.253	0.006	0.008	0.519	0.695	rs6955367
ILMN_2400759	CPVL	0.644	0.062	0.013	0.014	0.631	0.672	rs7313
ILMN_2262288	EEF1G	0.615	0.107	0.025	0.028	0.592	0.663	rs7124057
ILMN_1689177	PPAPDC1A	0.519	0.138	0.006	0.007	0.57	0.661	rs2182513
ILMN_2093720	THG1L	0.621	0.208	0.003	0.004	0.512	0.647	rs11738432
ILMN_1691772	ZSCAN29	0.19	0.14	0.011	0.013	0.548	0.638	rs12912744
ILMN_1809147	FAM118A	0.282	0.07	0.002	0.002	0.589	0.634	rs104664
ILMN_2130441	HLA-H	1.386	0.092	0.033	0.037	0.573	0.628	rs2975033
ILMN_2388272	MED24	3.036	0.043	0.007	0.007	0.597	0.624	rs8070454
ILMN_2064132	NANP	0.283	0.194	0.005	0.006	0.502	0.62	rs2387976
ILMN_1683279	PEX6	0.164	0.245	0.006	0.008	0.46	0.609	rs2395943
ILMN_2312606	IRF5	0.662	0.181	0.005	0.006	0.496	0.606	rs10239340
ILMN_2390162	PHF11	0.117	0.165	0.015	0.018	0.501	0.601	rs1046028
ILMN_1710903	MAPT	0.118	0.052	0.01	0.011	0.552	0.584	rs1981997
ILMN_1811048	GPR107	0.519	0.354	0.002	0.004	0.374	0.58	rs2240913
ILMN_2201966	N4BP1	1.97	0.029	0.005	0.006	0.555	0.572	rs11649236
ILMN_1795336	PTER	0.591	0.143	0.012	0.014	0.486	0.567	rs7909832
ILMN_1789419	EXOC3	0.214	0.097	0.012	0.014	0.508	0.563	rs11134054
ILMN_2364072	CLCNKA	1.356	0.044	0.009	0.01	0.535	0.562	rs1763601
ILMN_2075334	HIST1H4C	0.213	0.296	0.007	0.01	0.395	0.561	rs198834
ILMN_2296011	BRWD1	0.821	0.413	0.005	0.009	0.328	0.559	rs6517526
ILMN_2074477	GPR4	1.342	0.058	0.016	0.017	0.526	0.558	rs10405576
ILMN_1697286	SF3A1	0.168	0.359	0.009	0.014	0.356	0.555	rs737950
ILMN_1765332	TIMM10	0.164	0.079	0.005	0.006	0.507	0.551	rs2848630
ILMN_2401641	ALDH3A2	0.22	0.142	0.012	0.014	0.472	0.543	rs2108971
ILMN_2183938	LEMD3	0.335	0.084	0.026	0.029	0.495	0.541	rs10878255
ILMN_2198408	MFF	0.153	0.26	0.002	0.003	0.399	0.54	rs7560053
ILMN_1728199	POLE	0.113	0.175	0.006	0.007	0.446	0.538	rs4883627
ILMN_1655637	UPK1A	1.044	0.131	0.023	0.026	0.467	0.538	rs4806187
ILMN_2209027	RPS26	0.238	0.146	0.002	0.002	0.457	0.535	rs10876864

Results from some of the top probes are depicted. Only one probe is selected per gene for depiction. R2technical = variance due to technical variables only (i.e. plates, RIN). addR2covariates = added proportion of variance due to biological covariates (i.e. age, sex, ApoE4 dose), adjR2covariates = addR2covariates adjusted for technical variance, addR2best-SNP = proportion of variance due to the best *cis*SNP, adjR2best-SNP = addR2best-SNP adjusted for technical variance.

The top 2,980 cerebellar eGWAS associations were followed up in the temporal cortex validation study. We found that 2,685 top cerebellar *cis*SNP/transcript associations could be tested in the temporal cortex (2,387 unique *cis*SNPs, 677 unique probes and 625 unique genes) (Figure 1, Table 3, Supplementary Table 6 in Dataset S1). A total of 2,089 of these (1,888 unique *cis*SNPs, 502 unique probes and 471 unique genes) were significant after study-wide Bonferroni corrections, many of which had effect sizes showing remarkable similarity to those from the cerebellar eGWAS (Pearson's correlation coefficient = 0.94, p<0.0001).

**Table 3:** Validation of top cerebellar *cis*SNP/transcript associations in the temporal cortex.

				Cerebellar eGWAS				Temporal Cortex Validation		
CHR	SNP	PROBE	SYMBOL	ALL_P	ALL_P_Bonf_	ALL_Q	ALL_BETA	ALL_P	ALL_P_Bonf-study_	ALL_BETA
5	rs3776455	ILMN_1718932	MTRR	6.37E-133	2.83E-127	7.34E-120	1.20	5.15E-133	1.38E-129	1.23
12	rs10843881	ILMN_2345908	DDX11	1.48E-112	6.57E-107	2.39E-100	−1.06	1.81E-113	4.87E-110	−1.16
12	rs1971762	ILMN_1807798	ATP5G2	1.10E-104	4.87E-99	1.64E-93	1.01	7.83E-107	2.10E-103	1.15
21	rs2838859	ILMN_2376667	POFUT2	2.86E-103	1.27E-97	2.73E-92	−0.86	4.15E-115	1.11E-111	−0.84
8	rs3802266	ILMN_2184966	ZHX2	4.35E-101	1.93E-95	3.06E-90	−1.94	1.11E-104	2.97E-101	−2.35
19	rs260462	ILMN_2052079	ZNF544	8.26E-101	3.67E-95	4.38E-90	−1.16	7.00E-129	1.88E-125	−1.30
17	rs2525574	ILMN_2369018	EVI2A	1.52E-99	6.73E-94	4.88E-89	1.81	4.56E-89	1.22E-85	1.67
7	rs6955367	ILMN_1723984	PILRB	4.80E-97	2.13E-91	1.23E-86	1.08	1.70E-141	4.56E-138	1.56
7	rs7313	ILMN_2400759	CPVL	5.00E-92	2.22E-86	5.96E-82	−0.96	5.76E-71	1.55E-67	−1.34
11	rs7124057	ILMN_2262288	EEF1G	2.64E-91	1.17E-85	2.67E-81	−0.90	1.49E-91	4.01E-88	−0.70
10	rs2182513	ILMN_1689177	PPAPDC1A	1.08E-87	4.79E-82	6.93E-78	0.84	8.07E-01	NS	0.01
5	rs11738432	ILMN_2093720	THG1L	8.06E-84	3.58E-78	3.49E-74	−0.86	1.16E-100	3.12E-97	−1.09
15	rs12912744	ILMN_1691772	ZSCAN29	3.47E-83	1.54E-77	1.33E-73	−0.56	6.18E-96	1.66E-92	−0.63
22	rs136564	ILMN_1809147	FAM118A	8.44E-81	3.74E-75	2.57E-71	0.85	7.87E-57	2.11E-53	0.68
17	rs8070454	ILMN_2388272	MED24	3.51E-80	1.56E-74	9.58E-71	−2.03	3.97E-85	1.06E-81	−2.16
20	rs2387976	ILMN_2064132	NANP	9.58E-79	4.25E-73	1.93E-69	−0.54	1.97E-87	5.29E-84	−0.65
7	rs10239340	ILMN_2312606	IRF5	1.52E-76	6.73E-71	2.31E-67	0.81	3.76E-90	1.01E-86	0.92
6	rs2395943	ILMN_1683279	PEX6	3.63E-76	1.61E-70	5.06E-67	−0.39	1.64E-69	4.40E-66	−0.46
13	rs1046028	ILMN_2390162	PHF11	1.07E-75	4.73E-70	1.36E-66	0.35	5.38E-83	1.44E-79	0.38
17	rs1981997	ILMN_1710903	MAPT	4.16E-71	1.85E-65	3.88E-62	−0.48	2.42E-44	6.48E-41	−0.51
9	rs2240913	ILMN_1811048	GPR107	2.76E-69	1.23E-63	2.39E-60	0.66	2.11E-80	5.66E-77	0.95
16	rs11649236	ILMN_2201966	N4BP1	5.69E-69	2.53E-63	4.37E-60	−1.57	1.95E-85	5.25E-82	−1.44
10	rs7909832	ILMN_1795336	PTER	1.70E-68	7.54E-63	1.04E-59	−0.75	1.24E-84	3.32E-81	−1.00
5	rs11134054	ILMN_1789419	EXOC3	4.40E-68	1.95E-62	2.52E-59	−0.57	1.51E-78	4.06E-75	−0.60
19	rs10405576	ILMN_2074477	GPR4	5.76E-68	2.56E-62	3.09E-59	−1.32	5.20E-62	1.39E-58	−1.53
6	rs198834	ILMN_2075334	HIST1H4C	4.76E-67	2.11E-61	2.05E-58	−0.43	1.25E-51	3.35E-48	−0.52
22	rs737950	ILMN_1697286	SF3A1	2.05E-66	9.11E-61	7.72E-58	0.44	1.01E-71	2.71E-68	0.46
1	rs1763601	ILMN_2364072	CLCNKA	3.49E-66	1.55E-60	1.24E-57	1.22	6.22E-50	1.67E-46	1.02
12	rs10878255	ILMN_2183938	LEMD3	2.61E-65	1.16E-59	7.91E-57	−0.62	1.73E-69	4.65E-66	−0.78
19	rs4806187	ILMN_1655637	UPK1A	2.82E-65	1.25E-59	8.11E-57	−1.04	3.82E-39	1.02E-35	−0.72
11	rs2848630	ILMN_1765332	TIMM10	3.40E-65	1.51E-59	9.32E-57	−0.53	2.04E-68	5.48E-65	−0.45
17	rs962800	ILMN_2401641	ALDH3A2	3.31E-64	1.47E-58	8.21E-56	0.46	4.26E-76	1.14E-72	0.44
2	rs7560053	ILMN_2198408	MFF	6.41E-63	2.85E-57	1.38E-54	0.37	5.20E-71	1.40E-67	0.30
12	rs10876864	ILMN_2209027	RPS26	6.36E-62	2.82E-56	1.06E-53	0.49	3.01E-64	8.07E-61	0.57
12	rs4883627	ILMN_1728199	POLE	8.61E-62	3.82E-56	1.36E-53	0.33	3.95E-19	1.06E-15	0.22
6	rs2191651	ILMN_1694100	PRIM2	1.55E-61	6.88E-56	2.26E-53	−0.59	5.06E-57	1.36E-53	−0.57
10	rs9527	ILMN_2151056	C10orf32	2.90E-61	1.29E-55	3.90E-53	−0.46	1.27E-65	3.42E-62	−0.45

Of the 2,980 top *cis*SNP/transcript associations, 2,685 existed in the temporal cortex replication study. Some of these top associations are shown. Only one *cis*SNP/transcript pair is selected for depiction. The chromosome (CHR), SNP, Probe, Gene Symbol (SYMBOL) of these associations are depicted. The uncorrected (P), genome-wide (P_Bonf_) and study-wide Bonferroni-corrected (P_Bonf-study_) P values, Beta coefficient of association are shown for the combined (All) analyses in the cerebellar eGWAS and the temporal cortex replication study. Regression coefficients are based on the SNP minor allele using an additive model.

The top cerebellar eGWAS results were also compared to published liver [23] and brain [24], [25] eGWAS and overlap was identified for 4–11% of the top transcripts from these published studies (Text S1) Using HapMap2 genotypes, all transcripts and association threshold p<1.0E-4 in our eGWAS, we determined that 24–32% of the top transcripts from the published eGWAS overlapped with ours.

We used the cerebellar eGWAS as the discovery analysis and the temporal cortex eGWAS as the validation; since our goal is to identify significant *cis*SNP associations while minimizing any confounding factors due to pathology and given the fact that half of our subjects had pathologic AD, in which cerebellum is relatively unaffected whereas temporal cortex is one of the first affected brain regions. Nonetheless, we have also used temporal cortex as the discovery set and cerebellum as the validation, with remarkably similar results (Text S1, Supplementary Tables 7 and 8 in Dataset S1).

### Enrichment of brain *cis*SNPs among human disease-associated SNPs

To examine whether the brain eGWAS approach identified variants implicated in human diseases/traits, we linked the 2,596 top cerebellar eGWAS *cis*SNPs to the “Catalog of Published GWAS” [26], which compiles weekly search results from all published GWAS of ≥100,000 SNPs where associations of p≤1.0E-05 are reported. We identified 47 *cis*SNPs that were also associated with 36 diseases/traits (Table 4, Supplementary Table 9 in Dataset S1). This represents a 2.4-fold enrichment of significant cerebellar *cis*SNPs amongst disease/trait associated SNPs, which is significant (p<10^−6^) based on simulations adjusted for minor allele frequencies [3] (Text S1).

**Table 4:** Examples of top cerebellar eGWAS *cis*SNPs also associated with complex diseases/traits.

		Cerebellar eGWAS					Disease GWAS					
CHR	SNP	MinorAllele	PROBE	SYMBOL	ALL_P	ALL BETA	PUBMED ID	Disease/Trait	Reported Gene(s)	Strongest SNP-Risk Allele	p-Value	OR or beta
7	rs4132601	C	ILMN_1676575	IKZF1	1.63.E-21	−0.36	19684604	Acute lymphoblastic leukemia (childhood)	IKZF1	rs4132601-C	1.00E-19	1.69
19	rs260461	A	ILMN_2052079	ZNF544	3.10.E-16	0.73	18821565	Attention deficit hyperactivity disorder	ZNF544	rs260461-?	8.00E-06	NR
11	rs10838738	G	ILMN_2195462	C1QTNF4	1.48.E-09	−0.18	19079261	Body mass index	MTCH2	rs10838738-G	5.00E-09	0.07
2	rs13015714	C	ILMN_1781700	IL18R1	8.30.E-24	0.55	18311140	Celiac disease	IL1RL1,IL18R1,IL18RAP, SLC9A4	rs13015714-C	4.00E-09	1.28
2	rs13015714	C	ILMN_2313672	IL1RL1	1.22.E-13	0.84	18311140	Celiac disease	IL1RL1,IL18R1,IL18RAP, SLC9A4	rs13015714-C	4.00E-09	1.28
7	rs2252521	A	ILMN_2400759	CPVL	1.86.E-18	0.55	19734545	Cognitive performance	CPVL	rs2252521-?	5.00E-06	NR
14	rs4444235	G	ILMN_1740900	BMP4	3.20.E-18	−0.27	19011631	Colorectal cancer	BMP4	rs4444235-C	8.00E-10	1.11
15	rs748404	G	ILMN_1691772	ZSCAN29	2.29.E-40	−0.42	19654303	Lung cancer	TGM5	rs748404-?	1.00E-06	1.15
6	rs499818	A	ILMN_1661622	TBC1D7	1.32.E-15	0.17	17903304	Major CVD	Intergenic	rs499818-?	7.00E-06	NR
17	rs6565681	A	ILMN_1749722	RNF213	2.10.E-22	−0.38	21048783	Moyamoya disease	RNF213	rs6565681-A	2.00E-08	4.82
6	rs2517713	C	ILMN_2130441	HLA-H	8.27.E-15	−0.66	19664746	Nasopharyngeal carcinoma	HLA-A	rs2517713-A	4.00E-20	1.88
6	rs3129055	G	ILMN_2203729	HCG4	1.90.E-14	−0.7	19664746	Nasopharyngeal carcinoma	HLA-F	rs3129055-G	7.00E-11	1.51
10	rs1561570	G	ILMN_2381899	OPTN	1.51.E-11	0.07	20436471	Paget's disease	OPTN	rs1561570-?	6.00E-13	1.54
17	rs8070723	G	ILMN_1710903	MAPT	7.02.E-69	−0.47	21044948	Parkinson's disease	MAPT	rs8070723-?	7.00E-12	1.3
17	rs11012	A	ILMN_2393693	LRRC37A4	1.69.E-33	−0.52	20070850	Parkinson's disease	PLEKHM1, MAPT, IMP5	rs11012-T	6.00E-08	1.43
17	rs11012	A	ILMN_2286783	LRRC37A4	1.64.E-37	−0.65	20070850	Parkinson's disease	PLEKHM1, MAPT, IMP5	rs11012-T	6.00E-08	1.43
4	rs6532197	G	ILMN_1660114	MMRN1	4.86.E-12	0.52	19915575	Parkinson's disease	MMRN1	rs6532197-G	1.00E-07	1.32
11	rs538147	A	ILMN_1772208	CCDC88B	3.92.E-22	−0.27	21399635	Primary biliary cirrhosis	RPS6KA4	rs538147-G	2.00E-10	1.23
2	rs13385191	G	ILMN_1660275	C2orf43	7.01.E-22	−0.26	20676098	Prostate cancer	C2orf43	rs13385191-G	8.00E-08	1.15
5	rs27524	A	ILMN_2336220	ERAP1	9.60.E-17	0.3	20953190	Psoriasis	ERAP1	rs27524-A	3.00E-11	1.13
20	rs1008953	A	ILMN_1756590	SYS1	4.15.E-10	−0.09	20953189	Psoriasis	SDC4	rs1008953-C	1.00E-07	1.14
7	rs4728142	A	ILMN_2312606	IRF5	2.30.E-24	−0.52	19838193	Systemic lupus erythematosus	IRF5	rs4728142-A	8.00E-19	1.43
6	rs2301271	A	ILMN_1700428	HLA-DOB	1.27.E-11	0.38	21408207	Systemic lupus erythematosus	HLA-DQA2	rs2301271-T	2.00E-12	1.47
11	rs4963128	A	ILMN_2349061	IRF7	6.84.E-21	−0.27	18204446	Systemic lupus erythematosus	KIAA1542	rs4963128-?	3.00E-10	1.28
11	rs4963128	A	ILMN_2349061	IRF7	6.84.E-21	−0.27	21408207	Systemic lupus erythematosus	KIAA1542	rs4963128-?	4.00E-06	1.33
12	rs1701704	C	ILMN_2209027	RPS26	1.31.E-39	0.42	18198356	Type 1 diabetes	RAB5B, SUOX, IKZF4, ERBB3, CDK2	rs1701704-C	9.00E-10	1.25
12	rs3764021	A	ILMN_1782729	CLECL1	7.67.E-22	−0.39	17554300	Type 1 diabetes	NR	rs3764021-C	5.00E-08	1.57
15	rs8042680	A	ILMN_2395932	UNC45A	1.84.E-13	−0.26	20581827	Type 2 diabetes	PRC1	rs8042680-A	2.00E-10	1.07
7	rs4728142	A	ILMN_2312606	IRF5	2.30.E-24	−0.52	21297633	Ulcerative colitis	IRF5, TNPO3	rs4728142-A	2.00E-08	1.07
9	rs4077515	A	ILMN_1811301	INPP5E	2.74.E-13	−0.12	20228799	Ulcerative colitis	CARD9	rs4077515-C	5.00E-08	1.14

The top 2,980 cerebellar eGWAS *cis*SNPs were compared to the “Catalog of Published GWAS” (www.genome.gov/gwastudies). Some of the resulting 60 common associations are reported. The chromosome (CHR), SNP, eGWAS Minor Allele, Probe, Gene Symbol (SYMBOL) of these associations are depicted. The uncorrected (P) and Beta coefficient of associations are shown for the combined (All) analyses of the cerebellar eGWAS. Regression coefficients are based on the SNP minor allele using an additive model. The information for the complex disease/trait GWAS was downloaded from their website accessed on 04/23/2011. The disease/trait associated SNPs shown are the strongest SNPs depicted in the disease/trait GWAS. The associating allele (Strongest SNP-Risk Allele), p-value, OR or beta for the strongest disease/trait SNPs are shown.

Among the 36 diseases/traits associating with top cerebellar *cis*SNPs were central nervous system (CNS)-related conditions including Parkinson's disease (PD), Moyamoya disease, cognitive performance and attention-deficit hyperactivity disorder (ADHD). We both identified novel *cis*SNP/transcript associations and confirmed some previously reported ones. We found novel associations between rs6532197, which confers increased risk of PD [32], and higher brain levels of *MMRN1* (cerebellar eGWAS p = p_Cer_ = 4.86×10^−12^; temporal cortex eGWAS p = p_TCx_ = 4.57×10^−9^). *MMRN1* encodes for multimerin and was found to be in a region of duplication/triplication with *SNCA* (encoding α-synuclein), a well-established risk gene in PD [33]. We found no significant *cis*SNP/*SNCA* level associations. These results suggest that *MMRN1* may deserve further investigations as an additional PD risk gene.

Another example of a *cis*SNP which associates with human disease risk is rs8070723, the minor allele of which is associated with reduced risk of PD [32] and reduced brain *MAPT* levels (p_Cer_ = 3.36×10^−7^–7.02×10^−69^; p_TCx_ = 9.03×10^−4^–8.61×10^−44^). Rs11012 minor allele, which confers increased risk of PD [34], showed association with lower brain *LRRC37A4* levels (p_Cer_ = 1.69×10^−33^; p_TCx_ = 3.378E^−20^). *MAPT* region variants were previously identified to associate with brain levels of *MAPT* and *LRRC37A4* in neurologically normal subjects [27], [32], in a *MAPT* haplotype H1/H2-dependent manner [27]. Indeed, rs8070723 is in tight linkage disequilibrium with rs1052553 (r^2^ = 0.95, D′ = 0.97), the major allele of which marks the *MAPT*-H1 haplotype and associates with higher brain *MAPT* levels [24].

Many top cerebellar *cis*SNPs also associate with non–CNS diseases/traits (Supplementary Table 9 in Dataset S1). *IRF5 cis*SNP rs4728142 is associated with both cerebellar *IRF5* levels and risk of systemic lupus erythematosus (SLE) [35]. Previously, *IRF* variants were shown to influence *IRF* splicing and expression as well as SLE risk [36], [37]. Interestingly, rs4728142 is also associated with ulcerative colitis (UC) [38] where both *IRF5* and *TNPO3* are reported as candidate genes. Given its influence on *IRF5*, but not *TNPO3* expression levels, rs4728142 most likely marks *IRF5*, but not *TNPO3* as the candidate UC risk gene.

Our approach to identify human disease-associated SNPs amongst the 2,596 top cerebellar eGWAS *cis*SNPs may be overly conservative, given our selection criteria to only include transcripts that are detectable in >75% of the subjects and only those *cis*SNPs that are significant in both independent cohorts (ADs and non–ADs). Furthermore, given that our eGWAS genotyping platform consisted of ∼300 K SNPs, it is plausible that transcript associations with SNPs from the “Catalog of Published GWAS” [26] may be missed if those SNPs did not exist in our platform. To address these issues, we repeated the cerebellar and temporal cortex eGWAS, without restrictions for transcript detection rates and using genotypes imputed to HapMap2 (>2 million SNPs). Comparison of the eGWAS associations with p<1.0E-4 to the “Catalog of Published GWAS” identified 392 unique cerebellar *cis*SNPs that also associate with 189 human diseases/traits; and 339 such temporal cortex *cis*SNPs associating with 167 diseases/traits (Text S1, Supplementary Tables 10 and 11 in Dataset S1). Amongst the associations identified by this less stringent approach were those for brain levels of *CLU* [*39*], [*40*], *CR1* [*40*] and *GAB2* [*41*] which were identified as risk loci in GWAS of Alzheimer's disease.

We also performed comparisons of the eGWAS results from the ADs and non–ADs separately to determine whether there were any results unique to these diagnostic groups (Text S1, Supplementary Tables 12, 13, 14, 15 in Dataset S1). Although 13–25% of the disease/trait associations were with *cis*SNPs that were unique to ADs or non–ADs, all but a few of these could also be identified in the combined analysis of all subjects. There were only 2–7 human diseases/traits with *cis*SNP associations that were detectable just in ADs or non–ADs, but not the combined group.

Of these unique *cis*SNP, those that associate with cerebellar levels of *C9orf72* in non–ADs are interesting, as these variants were previously identified in GWAS of amyotrophic lateral sclerosis (ALS), where *C9orf72* was one of the candidate genes at the disease locus [42], [43]. This gene was recently identified as the most common cause of familial ALS, with a repeat expansion leading to loss of an alternatively spliced transcript [44], [45]. These results further support the utility of the combined eGWAS and disease GWAS approaches in the potential identification of disease genes with modified transcript levels as the plausible disease mechanism.

### Identification of brain *cis*SNPs among PSP GWAS loci

In a recent PSP GWAS [27], four loci near *MAPT*, *STX6*, *EIF2AK3*, and *MOBP* conferred significant risk, in addition to three suggestive loci at *1q41 intergenic locus*, *BMS1* and *SLCO1A2*. We assessed these seven strongest PSP risk loci in our eGWAS in the ADs, non–ADs and combined datasets, as well as the PSP subset of non–ADs (Table 5, Supplementary Table 16 in Dataset S1). We found novel, significant rs11568563 minor allele associations with reduced brain *SLCO1A2* levels (p_Cer_ = 2.33×10^−8^; p_TCx_ = 4.36×10^−2^–9.14×10^−18^), which confers increased PSP risk [27]. *SLCO1A2* encodes solute carrier organic anion transporter family member 1a2 and is a drug transporter into the CNS [46]. Fine-mapping of the *SLCO1A2* region revealed rs11568563 to be the strongest *cis*SNP influencing brain levels of this gene (Figure S5). This SNP was also identified as the top PSP-associating variant at this locus [27]. All other *cis*SNPs that associate with brain *SLCO1A2* levels have weaker effects that appear to be due to their LD with s11568563, which is a missense coding mutation within *SLCO1A2*. Whether rs11568563 is merely tagging the functional variant(s) regulating levels of *SLCO1A2* or coding changes also influence expressed transcript levels require further investigations. Additionally, *MAPT*/rs242557 minor allele increased PSP risk [27] and brain *MAPT* levels (p_Cer_ = 9.78×10^−3^–8.8×10^−13^, p_TCx_ = 1.1×10^−8^). *MAPT*/rs8070723 minor allele associated with lower brain *MAPT* levels in our eGWAS, decreased PSP risk [27], similar to a PD GWAS [32]. We also found nominally significant increases in brain *MOBP* levels (p_Cer_ = 2.13×10^−2^–1.71×10^−7^; p_TCx_ = 1.55×10^−2^–1.57×10^−6^) with rs1768208, which increases PSP risk [27].

**Table 5:** PSP GWAS *cis*SNP/transcript associations in the cerebellar and temporal cortex.

				ALL CER		PSP CER		AD CER		ALL TCX		PSP TCX		AD TCX		PSP GWAS^b^			
CHR	SNP	PROBE	Symbol	BETA	P	BETA	P	BETA	P	BETA	P	BETA	P	BETA	P	Gene	OR	CI	P
17	rs8070723	ILMN_1710903	MAPT	−0.473	7.02E-69	−0.428	1.38E-08	−0.429	1.49E-31	−0.498	8.61E-44	−0.276	9.05E-02	−0.495	3.12E-37	MAPT	5.11	4.43–5.91	1.5×10−118
12	rs11568563	ILMN_2381020	SLCO1A2	−0.397	2.33E-08	−0.371	3.62E-03	−0.438	1.98E-05	−0.730	9.14E-18	−0.644	3.89E-05	−0.715	1.23E-08	SLCO1A2	0.68	0.59–0.78	7.0×10−8
17	rs242557	ILMN_1710903	MAPT	0.183	8.80E-13	0.087	1.66E-02	0.179	2.39E-06	0.181	1.10E-08	−0.033	6.65E-01	0.251	4.03E-10	MAPT	0.51	0.48–0.55	2.7×10−71
3	rs1768208	ILMN_2298464	MOBP	0.363	1.71E-07	0.342	1.24E-02	0.334	3.70E-04	0.325	1.57E-06	0.342	2.33E-02	0.305	1.23E-03	MOBP	0.73	0.67–0.78	5.3×10−17
3	rs1768208	ILMN_2414962	MOBP	0.243	1.17E-04	0.220	8.02E-02	0.204	1.25E-02	0.180	1.76E-05	0.238	1.74E-02	0.137	8.18E-03	MOBP	0.73	0.67–0.78	5.3×10−17
17	rs8070723	ILMN_2298727	MAPT	−0.173	3.36E-07	−0.063	5.91E-01	−0.161	8.40E-04	−0.137	9.03E-04	−0.073	6.15E-01	−0.211	8.67E-05	MAPT	5.11	4.43–5.91	1.5×10−118
3	rs1768208	ILMN_1768947	MOBP	0.143	7.57E-03	0.079	4.55E-01	0.159	1.72E-02	0.107	3.03E-03	0.199	1.83E-02	0.067	1.38E-01	MOBP	0.73	0.67–0.78	5.3×10−17
1	rs1411478	ILMN_2157951	STX6	−0.005	6.41E-01	−0.031	1.32E-01	−0.002	8.97E-01	0.027	1.17E-02	−0.003	8.90E-01	0.041	8.50E-03	STX6	0.79	0.73–0.84	3.5×10-11
3	rs1768208	ILMN_1750271	MOBP	0.074	2.13E-02	0.064	2.84E-01	0.073	8.86E-02	0.046	1.55E-02	0.082	1.03E-01	0.045	4.29E-02	MOBP	0.73	0.67–0.78	5.3×10−17
12	rs11568563	ILMN_1656097	SLCO1A2	−0.077	2.41E-01	−0.027	8.14E-01	−0.117	2.12E-01	−0.117	4.36E-02	−0.061	6.08E-01	−0.064	4.22E-01	SLCO1A2	0.68	0.59–0.78	7.0×10−8
17	rs242557	ILMN_2310814	MAPT	0.002	8.27E-01	−0.032	9.13E-02	0.008	6.19E-01	−0.026	1.09E-01	−0.054	3.49E-01	−0.022	9.33E-02	MAPT	0.51	0.48–0.55	2.7×10−71
17	rs8070723	ILMN_2200636	KIAA1267	0.021	1.14E-01	0.046	2.87E-01	0.036	4.04E-02	−0.016	1.70E-01	−0.025	5.80E-01	−0.026	1.02E-01	MAPT	5.11	4.43–5.91	1.5×10−118
17	rs8070723	ILMN_1665311	STH	0.044	1.79E-02	0.114	7.56E-02	0.026	3.05E-01	0.051	1.86E-01	0.188	2.59E-01	−0.022	6.24E-01	MAPT	5.11	4.43–5.91	1.5×10−118
17	rs242557	ILMN_2298727	MAPT	0.071	9.78E-03	0.086	9.40E-02	0.075	8.56E-02	0.041	2.17E-01	−0.054	4.26E-01	0.110	2.26E-02	MAPT	0.51	0.48–0.55	2.7×10−71
1	rs1411478	ILMN_2167416	MR1	0.045	4.27E-02	0.092	4.48E-02	0.015	6.02E-01	0.029	2.24E-01	−0.002	9.66E-01	0.077	1.20E-02	STX6	0.79	0.73–0.84	3.5×10−11
17	rs8070723	ILMN_2310814	MAPT	−0.007	5.77E-01	−0.028	5.12E-01	0.007	6.94E-01	0.018	3.65E-01	0.039	7.52E-01	0.024	1.03E-01	MAPT	5.11	4.43–5.91	1.5×10−118
17	rs242557	ILMN_2200636	KIAA1267	−0.012	2.59E-01	−0.022	2.56E-01	−0.019	2.23E-01	0.008	3.83E-01	0.030	1.50E-01	−0.003	8.08E-01	MAPT	0.51	0.48–0.55	2.7×10−71
17	rs242557	ILMN_1665311	STH	−0.027	6.59E-02	−0.020	4.88E-01	−0.017	4.42E-01	−0.024	4.28E-01	−0.082	2.91E-01	0.027	4.89E-01	MAPT	0.51	0.48–0.55	2.7×10−71
1	rs1411478	ILMN_1721833	IER5	−0.041	2.12E-01	−0.074	2.56E-01	NA	NA	0.016	4.76E-01	0.025	5.73E-01	0.000	9.95E-01	STX6	0.79	0.73–0.84	3.5×10−11
10	rs2142991	ILMN_1772713	BMS1	0.001	9.36E-01	−0.007	8.18E-01	0.006	7.26E-01	0.003	8.02E-01	0.028	2.30E-01	−0.014	4.56E-01	BMS1	1.3	1.18–1.44	3.2×10−7
3	rs1768208	ILMN_1781231	SLC25A38	0.004	7.76E-01	−0.020	4.33E-01	0.025	1.47E-01	0.002	9.02E-01	0.001	9.78E-01	0.019	2.84E-01	MOBP	0.73	0.67–0.78	5.3×10−17
2	rs7571971	ILMN_1714809	RPIA	−0.035	7.03E-02	−0.071	9.57E-02	NA	NA	0.002	9.21E-01	0.027	5.19E-01	0.008	7.68E-01	EIF2AK3	0.75	0.70–0.82	4.2×10−13
3	rs1768208	ILMN_2411723	RPSA	−0.025	6.92E-02	−0.057	3.77E-02	−0.034	8.72E-02	0.000	9.66E-01	−0.008	7.29E-01	−0.001	9.41E-01	MOBP	0.73	0.67–0.78	5.3×10−17

Seven SNPs near six PSP candidate risk genes from a recent PSP GWAS [27] were tested for transcript associations in *cis* in our cerebellar (CER) eGWAS and temporal cortex replications. The chromosome (CHR), SNP, Probe, Gene Symbol (SYMBOL) of these associations are depicted. The uncorrected (P) and Beta coefficient of association are shown for the combined (All), PSP, AD analyses in the cerebellar eGWAS and the temporal cortex replication study. Regression coefficients are based on the SNP minor allele using an additive model. NA: Not available. a. Though a SNP is present in the sequence of probe ILMN_2298727 (rs73314997), this is essentially monomorphic in the eGWAS subjects. b. The odds ratios (OR), confidence intervals and P values of disease risk association from the PSP GWAS are also shown using results from Supplementary Table 2 in Dataset S1 of this study [27].

The recent PSP GWAS by Hoglinger et al. [27] included eQTL analysis for the significant loci using brain expression levels from 387 subjects without clinical neurologic diseases. In addition to associations between *MAPT* locus *cis*SNPs with brain *MAPT* and *LRRC37A4* levels, they also detected signals for the nearby *ARL17A* and *PLEKHM1* genes, neither of which were detectable in our eGWAS. They also identified *cis*SNP associations with brain *MOBP* levels but even stronger influence on the nearby *SLC25A38* levels. We did not identify significant *cis*SNP/*SLC25A38* brain expression associations. Although some of the significant probes for *MOBP* and *MAPT* harbor variants within their probe sequence, which may potentially confound associations with expression levels, these genes had other significant probes without any sequence variants (Text S1).

Most non–AD subjects in our study had pathologic diagnosis of PSP (n_Cer_ = 98, n_TCx_ = 107, Supplementary Table 2 in Dataset S1). We assessed the 2,980 top cerebellar *cis*SNP/transcript associations in this subset, and found that most results were consistent with the ADs (Supplementary Tables 17 and 18 in Dataset S1).

### Enrichment of brain *cis*SNPs in the AD GWAS from ADGC

To investigate whether any of the significant brain *cis*SNPs may influence risk of AD, we compared our eGWAS results to the AD risk associations from the large AD GWAS conducted by ADGC [28]. We obtained results of meta-analyses for the ADGC Stage 1+2 cohort (11,840 LOAD vs. 10,931 controls) [28] and investigated those SNPs with suggestive AD risk association in this dataset (p_meta_<10^−3^). To ensure uniform comparison between our eGWAS and the ADGC GWAS, we assessed results from >2 million SNPs for each study using SNPs genome-wide imputed to HapMap phase 2 (release 22). There were 77,126 cerebellar (63,652 unique SNPs, 2,338 unique genes) and 68,172 temporal cortex (57,922 unique SNPs and 2,201 unique genes) *cis*SNP/transcript associations significant at q<0.05 representing a clear excess (Figure S6). There were 380 *cis*SNPs that were significant for the cerebellar transcript associations and also had suggestive AD risk associations (2.9-fold enrichment), 432 such temporal cortex *cis*SNPs (3.3-fold enrichment) and 356 *cis*SNPs significant in both the cerebellum and temporal cortex (2.7-fold enrichment, p<10^−6^ for all three analyses) (Figure 1, Supplementary Tables 19 and 20 in Dataset S1).

*MAPT* and *LRRC37A4 cis*SNPs, implicated in PSP [27] and PD [32] GWAS and which significantly influenced brain levels of these genes also had suggestive AD risk associations (p_meta_ = 8.82×10^−4^–1.53×10^−5^). *Cis*SNP alleles associating with lower brain *MAPT* levels were associated with lower AD risk, similar to PD [32] and PSP [27] GWAS, which may suggest a common mechanism for these neurodegenerative diseases. *ABCA7*, identified recently as a novel LOAD risk locus [28], [47], had significant cerebellar *cis*SNPs. Further investigations of the other genes with evidence of brain transcript and AD risk association is warranted to understand their role in AD (Text S1).

To ensure that we did not miss any associations due to the stringent eGWAS criteria that we applied, we repeated the analyses using no restrictions for transcript detection rates and eGWAS p value threshold of p<1.0E-4. We also investigated *cis*SNPs identified in AD and non–AD brains, both separately, and jointly, given that some *cis*SNP associations may be unique to one group. We compared these eGWAS results to the ADGC GWAS as described above (Supplementary Tables 21, 22, 23, 24, 25, 26 in Dataset S1). Using cerebellar and temporal cortex eGWAS from all subjects, 561 and 488 unique transcripts with *cis*SNPs that yield suggestive AD risk associations were identified, respectively. There were 259–312 such transcripts identified in each AD or non–AD eGWAS, with >50% overlap between the two diagnostic groups' results, although many of these results could be identified in the eGWAS of combined samples. About 7–10% of the transcripts could only be identified in just ADs or non–ADs, but not the combined eGWAS. Amongst such unique transcripts were *CLU* and *BIN1*, which reside at the LOAD GWAS loci [39], [40], [48] and associate with *cis*SNPs in the cerebellum of non–ADs. Detailed analyses of the *CLU* locus *cis*SNP/transcript associations are *in-press* [*49*].

## Discussion

In a large eQTL study on 773 brain samples from ∼400 autopsied subjects, we demonstrate significant contribution of genetic factors to human brain gene expression, reliably detected across different brain regions and pathologies. There is significant enrichment of brain *cis*SNPs amongst disease-associated variants, advocating gene expression changes as a mechanism for the first time for certain genes implicated in human diseases, including PSP (*SLCO1A2*), PD (*MMRN1*), Paget's disease (*OPTN*) while replicating others (e.g. PD/*MAPT*, SLE/UC/*IRF5*). *MAPT cis*SNPs associating with PSP, PD and AD risk highlight potential common mechanisms for these neurodegenerative diseases.

The reported results have several important implications for the genetics of human brain gene expression: First, despite technical challenges of gene expression measurements in post-mortem brain tissue [50], ∼70% of the transcriptome can be reliably detected in >75% of the subjects across two brain regions and different disease pathologies. Second, although there is significant contribution from technical covariates, genetic factors account for a substantial proportion of the variance in brain gene expression levels. We estimate that genetic factors explain an average 3% (range: 0–85%) of the variance in human cerebellar gene expression overall, and 18% (range: 8–85%) of the variance for the top *cis*-regulated transcripts. These estimates show remarkable similarity to those from other eQTL studies, such as a large, family-based lymphocyte eQTL, where *cis* eQTLs had an overall median effect size of 1.8% and significant eQTLs accounted for 24.6% of the variance in expression [16]. Similarly, significant *cis*SNPs explained 2–90% of expression variance in a liver eGWAS [23].

Third, there is remarkable replication of significant *cis*SNP associations across different brain regions and underlying tissue pathologies. Indeed, the 2,980 top cerebellar *cis*SNP/transcript associations represent 58% and 68%of all significant associations in the ADs and non–ADs. Since >50% of the non–ADs were comprised of subjects with PSP, we also conducted a separate analysis of this pathologically distinct group of non–ADs and again determined that many of the top *cis*SNPs were also significant in the PSPs despite the small sample size (n = 98). Importantly, most of the *cis*SNPs had highly similar effect sizes in the ADs, non–ADs and PSP subset of non–ADs. Furthermore, 78% of the top cerebellar *cis*-associations were also significant in the temporal cortex. Cerebellum is a relatively unaffected region in AD, whereas temporal cortex is typically one of the first areas to harbor neuropathology [51]. It is not inherently evident whether the unaffected or affected tissue regions would be most suitable for eQTL studies. Whereas unaffected regions would have the advantage of minimizing confounding on expression measurements from pathology (such as inflammation and cell death), affected regions may be more relevant for disease-associated eQTL mapping. The substantial overlap in significant *cis*SNP associations between different brain regions and disease types in our study implies that sample size may be the most critical element of successful eQTL mapping. In other words, analysis of expression data collected in different tissue regions and diseases, provided there is careful statistical control, could greatly enhance power to detect eQTLs. Nevertheless, there may be important eQTLs that are specific to brain region and disease.

It is not obvious whether the *cis*SNP that display similar effects in different brain regions and different disease types would have relevance to human disease. The top 2,596 cerebellar *cis*SNPs that are significant in both ADs and non–ADs, and many of which are also significant in the temporal cortex are also enriched for variants implicated in human disease, including CNS disease, such as PD and PSP. Thus, the fourth implication of our study is that it may be possible to map disease-associated variants using eQTL studies conducted in unaffected tissue or unaffected subjects. In addition to providing a general characterization of the genetics of brain gene expression, this study successfully replicated many previously published *cis*SNP associations, such as rs8070723/ *MAPT* level, rs11012/ *LRRC37A4* level associations, both of which were implicated in PD. We found novel brain expression level associations for transcripts implicated in disease, including rs11568563 association with *SLCO1A2*, recently identified in a PSP GWAS. The disease-associating *cis*SNP associations identified in this study were not restricted to CNS diseases, but also included non–CNS diseases, such as SLE, where we replicated the previously published rs4728142/ *IRF5* level associations.

These findings imply that many disease-associated *cis*SNPs can influence gene expression idependently of tissue/region/pathology, and be mapped reliably in tissue which is unaffected, not disease-related or from unaffected subjects. Indeed, our findings are consistent with a study of lymphoblastoid cell lines from subjects affected and unaffected with asthma, where Dixon et al. [12] found no differences between asthmatics and non-asthmatics. Furthermore, they detected significant transcript level associations with SNPs that also associate with asthma. Emilsson et al. [20] performed eQTL mapping in both blood and adipose tissue and determined that >50% of significant adipose tissue *cis*SNPs were also significant in blood. This is similar to the overlap we detected for cerebellum and temporal cortex, though two brain regions are more likely to have similar eQTL profiles than two different tissues.

Although many *cis*SNP effects can be detected in many different tissue types and disease conditions as shown here and by others [12], [20], there conceivably exist expression variants which exert their effects in a tissue or disease-specific manner. For example in the eQTL comparing blood and adipose tissue, Emilsson et al. [20] also found that more transcripts from adipose tissue had significant correlations with obesity-related traits. In reality, both scenarios may be at play, such that some expression variants have more ubiquitous effects, whereas others may need tissue/cell/region/disease specific factors to exert their influence on gene expression. Indeed, many of the CNS disease related *cis*SNP associations in our brain eGWAS could not be identified in our comparison to a liver eGWAS [23] or an existing database for a LCL eGWAS [12], suggesting that disease-relevant tissue may be necessary to detect effects of certain *cis*SNPs, and highlighting the value of this brain eGWAS for CNS traits/conditions.

Despite the enrichment of our samples with tissue from AD subjects and our use of both cerebellar and temporal cortex tissue, we did not identify strong transcript associations for some of the top genes recently implicated in AD risk in large LOAD GWAS studies [28], [39], [40], [47], [48]. This could be because the AD risk variants in these genes exert their effects via mechanisms other than influencing transcript levels, namely changes in protein conformation. If so, even the negative results from an eGWAS could be informative in guiding the future deep-sequencing efforts which should focus on coding rather than non-coding, functional regions. Alternative explanations include technical shortcomings, such as inability to measure all transcript species, measurements of global rather than cell-specific gene expression, not including all tested disease-associated variants in our genotyping platform. We also need to consider that the top genes nearest the strongest variants from the LOAD GWAS may not be actual disease genes. These loci require further investigations to account for this possibility. Additionally, our criteria for selection of the top *cis*SNPs, requiring significance in both ADs and non–ADs, might be too stringent, thereby leading to some false negative results. Finally, it may be possible to identify additional disease-related expression variants by focusing on those that have differential influence in disease vs. non-disease tissue, although this was not a focus of analysis in this study. Given that our non–AD tissue also consisted of subjects with other neurodegenerative diseases, there may be more similarities with the AD tissue, making it more difficult to detect variants with differential disease-related expression-associations in our current study. Nevertheless, we did find associations with *cis*SNPs for *ABCA7*, a novel AD risk locus gene [28], [47] and *MAPT* [52], [53], [24], [54] implicated in AD.

It is important to emphasize that although the identification of transcript level associations provides another layer of confidence for disease-associating variants and genes, it is entirely possible that a variant in an LD region encompassing multiple genes, could be marking a functional disease variant in one gene and an expression variant in another gene. Thus, although highly useful in conjunction with disease association studies, eGWAS should be seen as a guide rather than ultimate evidence in disease-mapping efforts. Similarly, absence of eGWAS associations for a disease-associated variant should not be seen as contradictory evidence, but rather raise the possibility of alternative functional mechanisms for that variant.

Despite the wealth of information our study provides, we acknowledge several shortcomings. First, our non–ADs were not normal controls but often had other brain pathologies. It will be necessary to seek replication of these findings or novel *cis*SNP/transcript associations in normal brain tissue, as well. Second, we only focused on single SNP associations. The preliminary observations from our eGWAS findings suggest that multiple independent variants may affect brain expression levels of some genes, whereas others might be under the influence of a single strong variant. Finally, like any association study, it is not clear whether the *cis*SNPs identified in our eGWAS are themselves the functional SNPs or simply in LD with un-genotyped regulatory variants. Future studies focusing on analysis of haplotypes, SNPxSNP interactions, novel variant discovery and functional *in-vitro* studies testing effects of multiple variants are required to dissect the genetic variation underlying brain gene expression levels.

In summary, this cerebellar eGWAS study and the temporal cortex validations provide insight about the genetics of brain gene expression, a framework to guide future studies with respect to tissue/region/disease choice in eQTL studies, examples about the utility of this approach in gene mapping, replication of some known transcript associations and evidence for novel transcript associations in human disease. Combined eGWAS-disease GWAS approach may provide complementary information in mapping human disease and enable identification of functional variants that may not be possible by either approach alone.

The complete set of results from the brain eGWAS can be accessed at the National Institute on Aging Genetics of Alzheimer's Disease Data Storage (NIAGADS) website at http://alois.med.upenn.edu/niagads/. Questions about the dataset can be addressed to the corresponding author of this manuscript (taner.nilufer@mayo.edu).

## Methods

### Subjects

All subjects were participants in the published Mayo LOAD GWAS [29] as part of the autopsy-based series (AUT_60–80). All subjects had neuropathologic evaluation by DWD. All ADs had definite diagnosis according to the NINCDS-ADRDA criteria [55] and had Braak scores of ≥4.0. All non–ADs had Braak scores of ≤2.5, and many had brain pathology unrelated to AD (Supplementary Tables 1 and 2 in Dataset S1). Three-hundred forty subjects had measurements in both cerebellum and temporal cortex. This study was approved by the appropriate institutional review board.

### Expression genome-wide association study (eGWAS)

#### RNA extraction and gene expression measurements

Total RNA was extracted from frozen brain samples using the Ambion RNAqueous kit according to the manufacturer's instructions. The quantity and quality of the RNA samples were determined by the Agilent 2100 Bioanalyzer using the Agilent RNA 6000 Nano Chip. Transcript levels were measured using the Whole Genome DASL assay (Illumina, San Diego, CA). Probe annotations were done based on NCBI Ref Seq, Build 36.2. The RNA samples were randomized across the chips and plates using a stratified approach to ensure balance with respect to diagnosis, age, gender, RINs and APOE genotype. Replicate samples were utilized for QC and also for intra-class coefficient (ICC) estimations. Raw probe level mRNA expression data were exported from GenomeStudio software (Illumina Inc.) for preprocessing with background correction, variance stabilizing transformation, quantile normalization and probe filtering using the lumi package of BioConductor [56], [57] (Text S1). A probe with detectable signal in >75% of the samples was regarded as informative and used in subsequent analyses, although we also did supplementary analyses without imposing any restrictions based on probe detection levels. The number of informative probes differed slightly between the AD, non–AD and combined groups (Figure S7).

#### Genome-wide genotyping

Genotypes were generated using Illumina's HumanHap300-Duo Genotyping BeadChips and analyzed with an Illumina BeadLab Station (Illumina, San Diego, CA) at the Mayo Clinic Genotyping Shared Resource according to the manufacturer's protocols. The LOAD GWAS QC methods were previously published [29] (Text S1).

#### Statistical methods for eGWAS

Linear regression analysis to test for *cis*SNP/transcript associations were done in PLINK [58]. Preprocessed probe transcript levels were utilized as endophenotypes. Each probe was assessed separately, even though one gene may have multiple probes. *Cis*SNPs localized to ±100 kb flanking region of the gene targeted by the probe of interest, mapped according to NCBI Build 36, were assessed for transcript level associations, using an additive model, with the minor allele dosage (0, 1, 2) as the independent variable, and APOE ε4 dosage (0, 1, 2), age at death, gender, PCR plate, RIN, (RIN-RINmean)^2^ as covariates. The cerebellum and temporal cortex expression levels were analyzed separately. The ADs and non–ADs were analyzed both separately and jointly. The joint analyses included diagnosis as an additional covariate (AD = 1, non–AD = 0). We also ran analyses including the top 10 eigenvectors from EIGENSTRAT, and compared eGWAS results to those excluding the eigenvectors (Text S1, Figure S8, Supplementary Table 27 in Dataset S1) [59].

Q values used for multiple testing corrections are based on false-discovery rates [30] and were corrected for genomic inflation of significance (Text S1). In addition, permutations (p_perm-WY_) and Bonferroni adjustment were used for comparison of correction strategies. Permutation p values were obtained by shuffling the endophenotype, while maintaining the covariate structure, 10,000 times and applying the Westfall and Young [60] resampling-style stepdown approach to account for correlations between probes.

### Variance of gene expression

To assess the genetic contribution to the variance in human cerebellar gene expression, we first determined between-subject variance, as a percentage of the total variance in probe expression, using ICC [31] for 15 samples measured in replicate on 5–6 different plates and 2–3 different days.

Using multivariable linear regression models, we then calculated the proportion of variance in cerebellar gene expression levels that were explained by technical effects (PCR plate, RIN, (RIN-RINmean)^2^), biological covariates (APOE ε4 dosage, age at death, gender) and the “best” *cis*SNP for each probe. These analyses were carried out on the combined dataset consisting of cerebellar expression measurements from 374 subjects and 15,283 probes with at least one *cis*SNP (Text S1).

### Replication of top cerebellar eGWAS hits in the temporal cortex

We identified 2,980 *cis*SNP/transcript associations (2,596 unique SNPs, 746 unique probes and 686 unique genes) that achieved genome-wide significance within both the ADs and non–ADs analyses with q values<0.05. All 2,980 *cis*SNP/transcript associations achieved genome-wide significance with q<0.05 and p_Bonf_<0.05 in the combined ADs+non–ADs analysis. We sought validation of these hits in the temporal cortex of 399 subjects who had WG-DASL whole transcriptome measurements and whole-genome genotypes. RNA extractions, QC, WG-DASL measurements, transcript level detections and association analyses were performed for these temporal cortex samples, in the same manner as that for the cerebellar samples. After appropriate QC, 2,685 of the 2,980 top cerebellar *cis*SNP/transcript associations remained detectable among the temporal cortex results (2,387 unique SNPs, 677 unique probes and 625 unique genes).

### Comparison of cerebellar eGWAS results with other published complex disease and trait GWAS

To determine whether the cerebellar eGWAS captured variants implicated in complex diseases/traits, we compared the top 2,980 cerebellar eGWAS *cis*SNPs with the top disease/trait associated SNPs in the “Catalog of Published GWAS” [26], curated by the National Human Genome Research Institute (www.genome.gov/gwastudies). This catalog compiles weekly search results from all published GWAS of ≥100,000 SNPs where associations of p≤1.0E-05 are reported. The catalog accessed on 04/23/2011 had 5,272 entries. We restricted our search to those entries where the “SNPs” column had only one SNP with an rs number. Thus, haplotypes and variants without rs numbers were excluded. There were 5,101 entries after this exclusion, comprised of 4,248 unique SNPs and 433 unique diseases. One SNP may associate with >1 disease/trait and each disease/trait may have ≥1 associating SNP. This list was linked to the 2,980 top cerebellar *cis*SNPs by common rs numbers.

To assess whether the number of observed *cis*SNPs that have both significant cerebellar eGWAS and disease/trait associations represent a significant enrichment, we performed simulations while adjusting for *cis*SNP minor allele frequencies, as previously reported [3]. We performed 1 million simulations and adjusted for the minor allele frequencies of all the tested *cis*SNPs in 10 bins from 0–0.05 to 0.45–0.50. Using the total number of *cis*SNPs that are both transcript and disease/trait associating for each simulation, we obtained an empirical p value and an estimate of fold-enrichment.

Cerebellar eGWAS results were also compared to other published eGWAS results from a human liver [23] and two human brain [24], [25] studies. The methods and results are depicted in Text S1.

### Alzheimer's Disease Genetics Consortium (ADGC) meta-analyses

To determine whether any of the *cis*SNPs significant at q<0.05 influenced risk of AD, we obtained meta-analyses results from the ADGC [28]. The cohorts that are assessed by ADGC, as well as the methodological details of the meta-analyses are described in detail in a recent publication [28]. Briefly, the meta-analyses of the ADGC dataset results reported here (Supplementary Tables 17 and 18 in Dataset S1) are generated from the combined analyses of stage 1 and stage 2 cohorts (Text S1), with detailed descriptions provided elsewhere [28]. Stage 1 cohorts are comprised of 8,309 LOAD cases and 7,366 cognitively normal elder controls. Stage 2 has 3,531 LOAD vs. 3,565 control subjects. Each cohort was tested for AD risk association using a logistic regression approach, assuming an additive model and adjusting for age, sex, APOE ε4 dosage and principal components from EIGENSTRAT [59]. The meta-analyses results were generated using the inverse variance method implemented in the software package METAL [61].

## Supporting Information

Dataset S1 This file includes Supplementary Tables 1–27. The individual supplementary table legends are included in the first tab of this file.
(XLS)

Figure S1 Q-Q-Plots: Q-Q plots of observed (y-axis) versus expected (X-axis) −log(p) values of association for all *cis*SNP/transcript associations in the combined cerebellar 374 samples obtained before (a,b) and after (c,d) inflation-adjustments. Q-Q plots for all data points (a, c), as well as those that are in the lower, left hand corner (b,d) are shown. The data in b and d account reflect the association results, where there should be no deviations from the expected (i.e. null hypothesis of no association).
(PDF)

Figure S2 Venn diagram of significant cerebellar *cis*SNP/transcript associations: Q values<0.05 in the ADs, non–ADs and combined (All) analyses. Notably, 2,980 cis-SNP/transcript associations are significant both in the ADs and non–ADs.
(PDF)

Figure S3 Box Plots of some top *cis*SNP/transcript associations in the non–AD (a), AD (b) and combined groups (c): The SNP genotypes are shown on the X-axis with the genotype counts in parentheses. Variance stabilizing transformed (VST) expression levels are on the Y-axis. The bottom and top of a box represent the lower and upper quartiles, respectively. The band near the middle of the box is the median. The ends of the whiskers depict the most extreme observations still within 1.5 inter quartile range of the corresponding quartile. Any data not included between the whiskers are plotted as dots.
(PDF)

Figure S4 Histogram of intra-class coefficients (ICC) for the cerebellar probe expressions. Using 15 replicate samples, ICC, which is the between-subject variance, as a percentage of the total variance in probe expression, was estimated for 17,121 probes.
(JPG)

Figure S5 Data plots of SNPs tested for association with expression levels of *SLCO1A2* in the Temporal Cortex and Cerebellum. Forty-six SNPs were tested for association of *SLCO1A2* levels in the Cerebellum (Blue lines) and Temporal Cortex (Pink lines). P-values were transformed using −log_10_ and are plotted against the position of each SNP along the chromosome (Kbp). Genes found within the locus boundaries are shown from the UCSC genome browser (http://genome.ucsc.edu/). The LD across the locus is represented by a plot generated with Haploview, using data from the Mayo GWAS. The top eSNP in this study, rs11568563, is highlighted on the p-value plot by red squares and a red box around the SNP in the list of rs numbers. This is also the top PSP-associating SNP at this locus in Hoglinger et al. (*Nat Genet*, 2011) [27].
(PDF)

Figure S6 Q-Q-Plots for cerebellar and temporal cortex cisSNP/transcript associations with the HapMap phase 2 imputed genotypes: Q-Q plots of observed (y-axis) versus expected (X-axis) −log(p) values of association for all *cis*SNP/transcript associations in the combined dataset obtained before (a) and after (b) genomic inflation-adjustments, as discussed in the text. Also shown are the Q-Q plots for the temporal cortex associations in the combined dataset obtained obtained before (c) and after (d) inflation-adjustments.
(PDF)

Figure S7 Venn diagram of detectable cerebellar probes. Venn diagram of cerebellar probes detectable in ≥75% of subjects in the AD (AD), non–AD (CON) and combined (All) analyses. Notably, 13,349 probes were detectable in all 374 subjects.
(PDF)

Figure S8 Scatterplots of −log10 p values for eGWAS associations with and without inclusion of eigenvectors. Transformed P-values of a) Cerebellar and b) Temporal Cortex eGWAS *cis*SNP/transcript associations from models including (y-axis) and excluding (x-axis) the top 10 eigenvectors are plotted. A linear line demonstrating the null hypothesis of no deviation of the results between the two datasets is also shown. The results are displayed for those SNPs with a Hardy-Weinberg P-value>1.0E-07 and a probe detection threshold >75%.
(JPG)

Text S1 Supplementary Results, Methods and References.
(DOC)
