# Supplementary material for: Brain Expression Genome-Wide Association Study (eGWAS) Identifies Human Disease-Associated Variants
Source: PLoS Genet. 2012 Jun 7;8(6):e1002707. doi: 10.1371/journal.pgen.1002707 (PMC3369937; doi:10.1371/journal.pgen.1002707)

Supplementary_Figure_1a

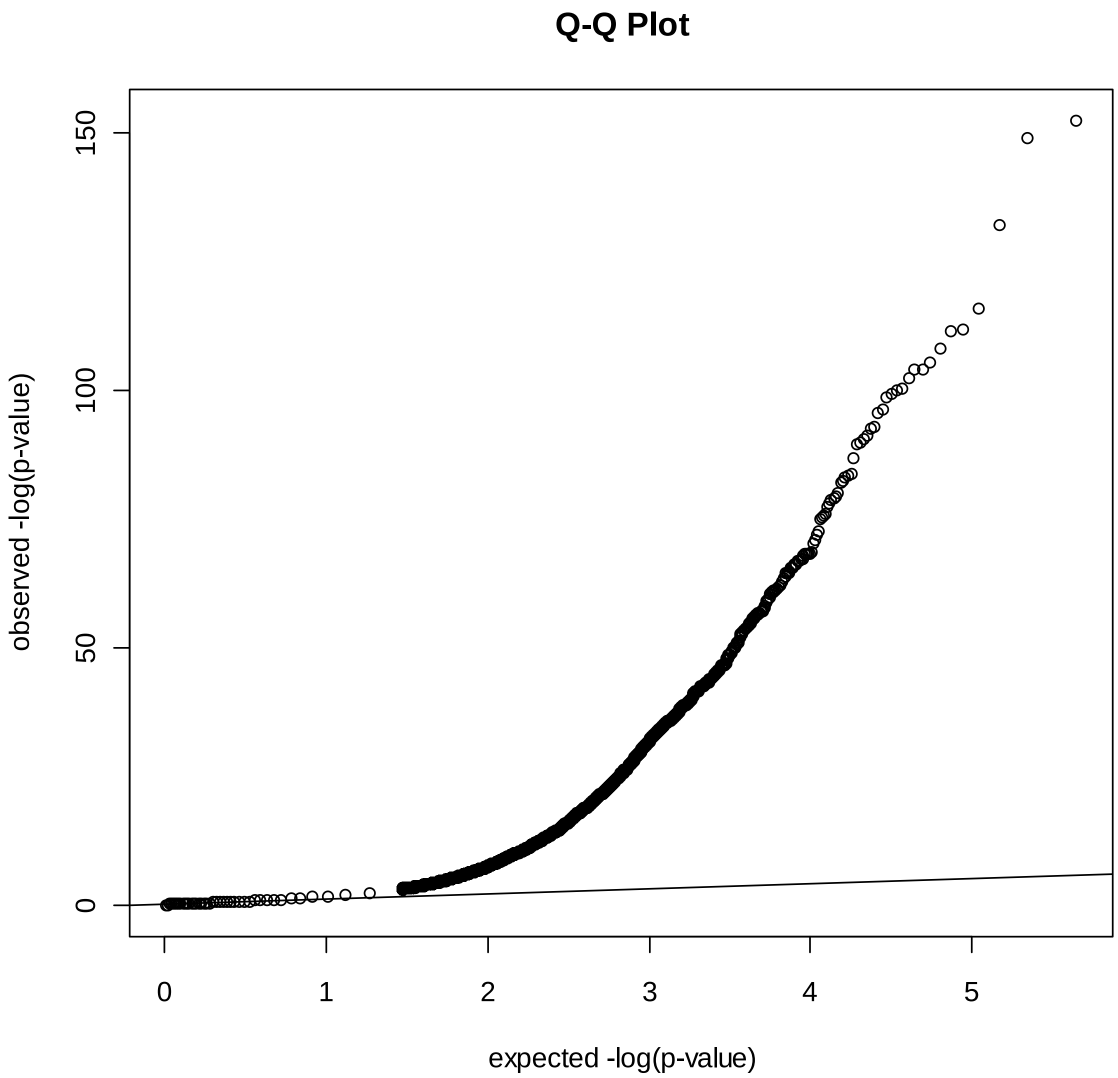

Supplementary_Figure_1b

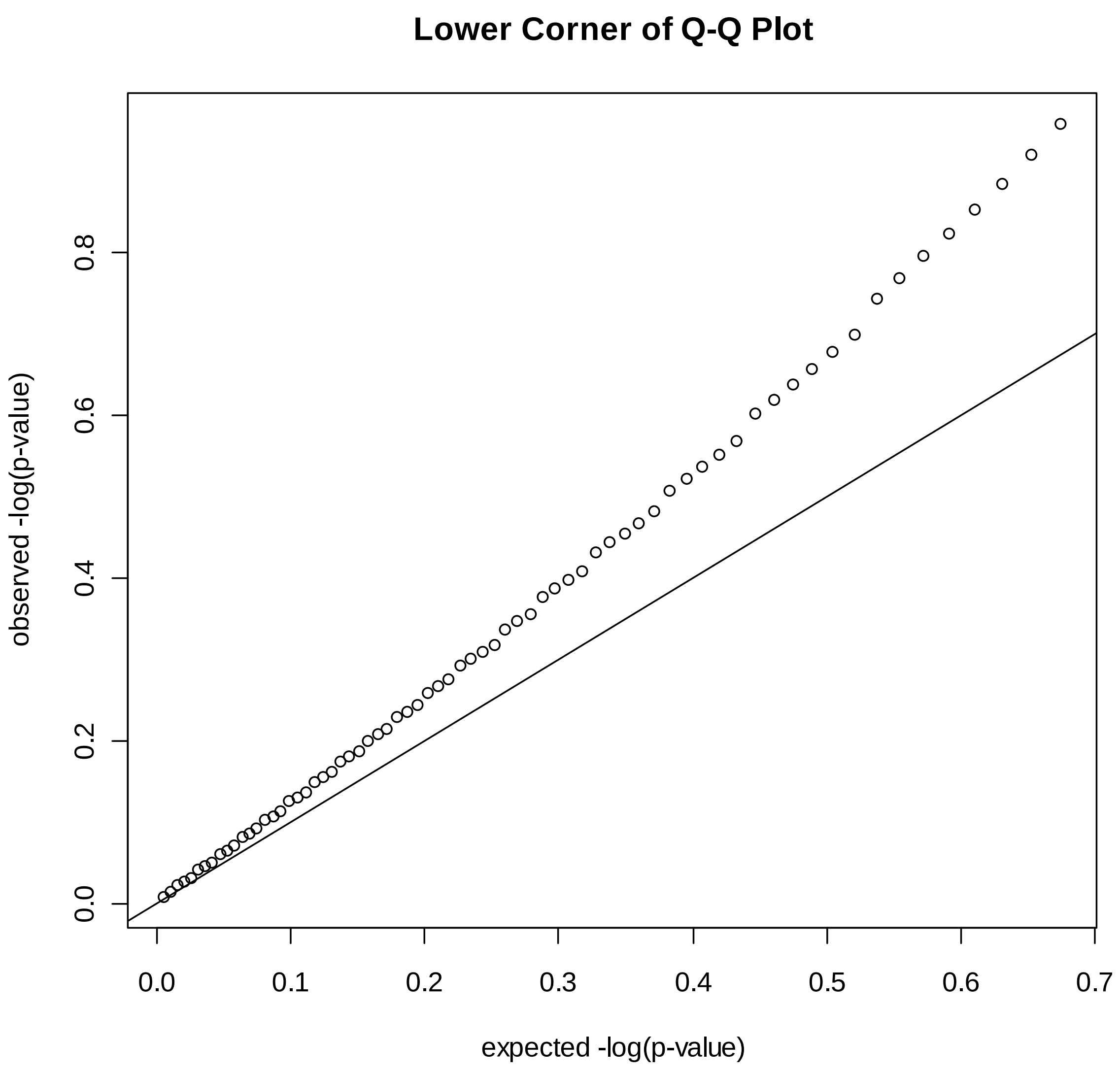

Supplementary_Figure_1c

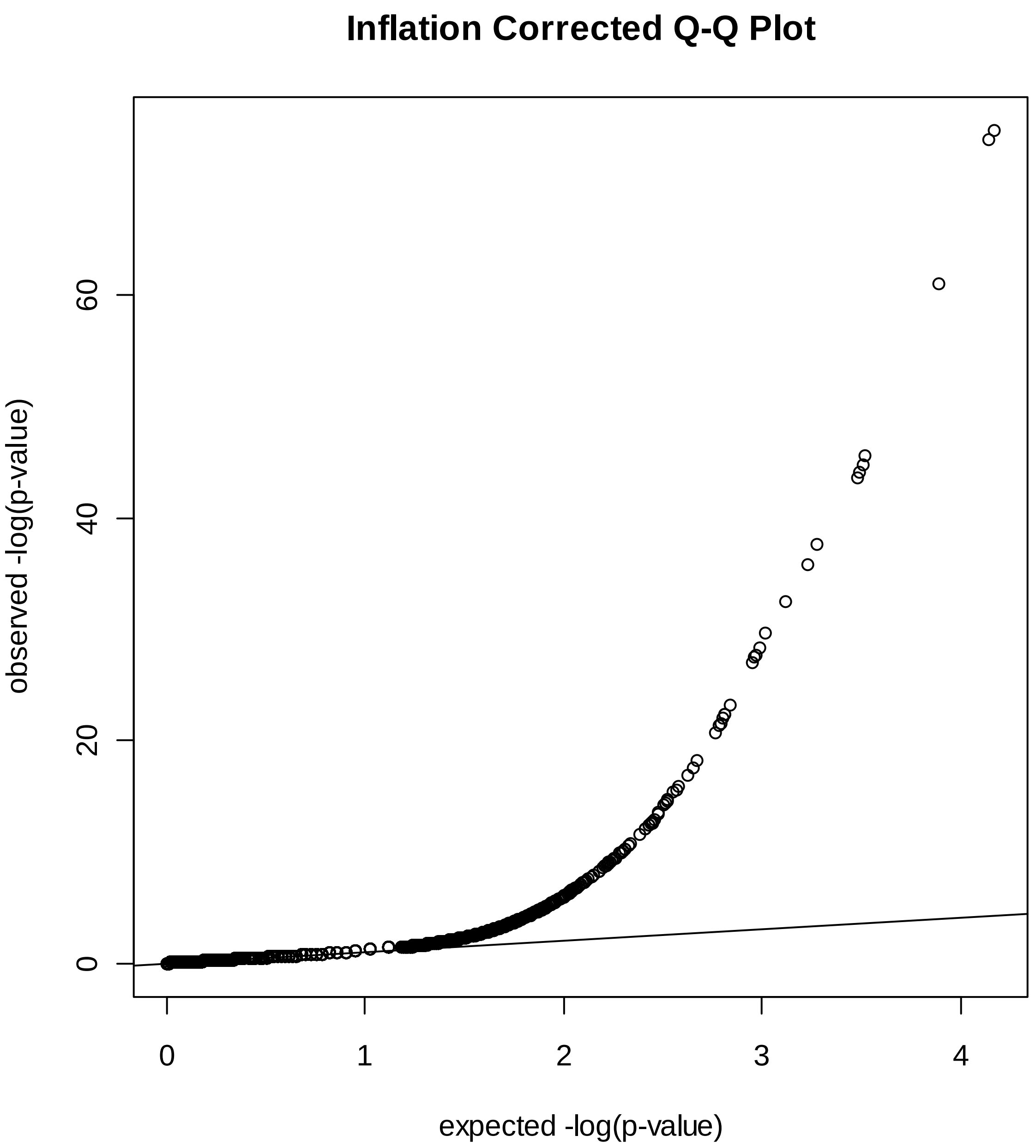

Supplementary_Figure_1d

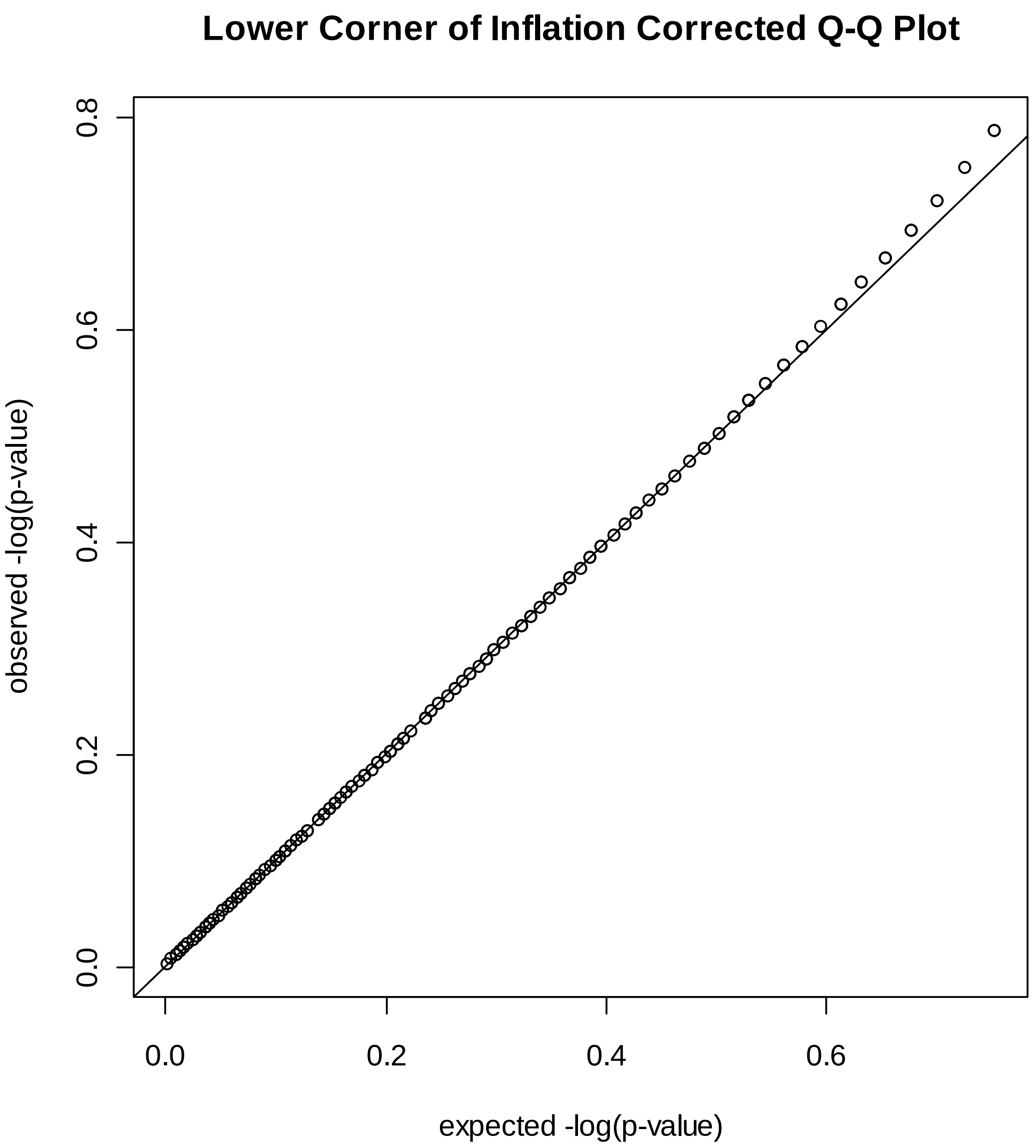

Supplement: Figure S1 — Q-Q-Plots: Q-Q plots of observed (y-axis) versus expected (X-axis) −log(p) values of association for all cisSNP/transcript associations in the combined cerebellar 374 samples obtained before (a,b) and after (c,d) inflation-adjustments. Q-Q plots for all data points (a, c), as well as those that are in the lower, left hand corner (b,d) are shown. The data in b and d account reflect the association results, where there should be no deviations from the expected (i.e. null hypothesis of no association). (PDF) [file pgen.1002707.s002.pdf]
