# Supplementary material for: Brain Expression Genome-Wide Association Study (eGWAS) Identifies Human Disease-Associated Variants
Source: PLoS Genet. 2012 Jun 7;8(6):e1002707. doi: 10.1371/journal.pgen.1002707 (PMC3369937; doi:10.1371/journal.pgen.1002707)
Supplement: Figure S2 — Venn diagram of significant cerebellar cisSNP/transcript associations: Q values<0.05 in the ADs, non–ADs and combined (All) analyses. Notably, 2,980 cis-SNP/transcript associations are significant both in the ADs and non–ADs. (PDF) [file pgen.1002707.s003.pdf]

Supplementary_Figure_2

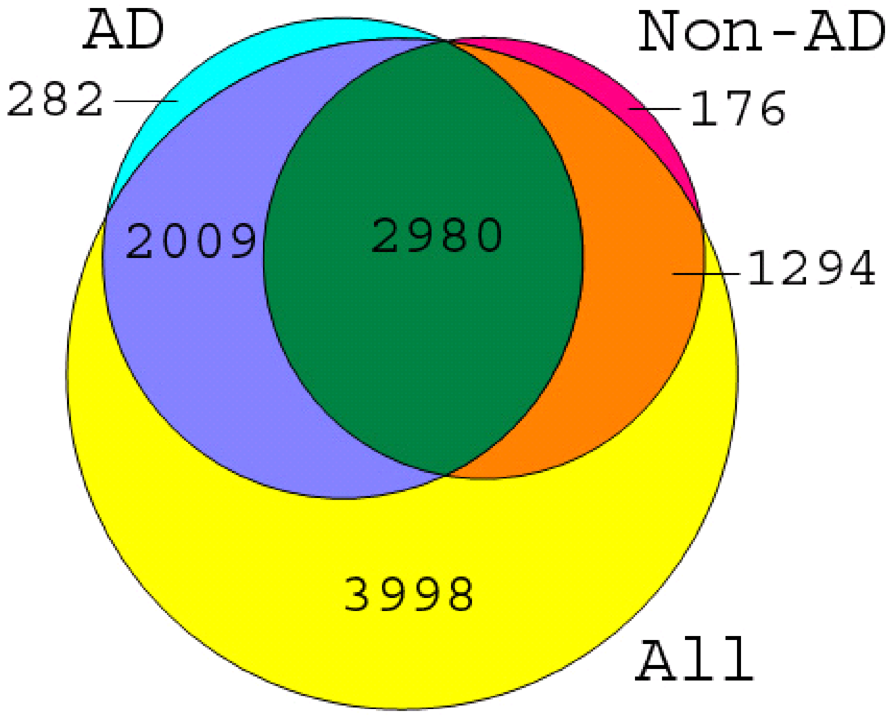

Supplementary_Figure_2_Legend: Venn diagram of significant cerebellar *cis*SNP/transcript associations: Qc values < 0.05 in the ADs, non-ADs and combined (All) analyses. Notably, 2,980 cis-SNP/transcript associations are significant both in the ADs and non-ADs.
