# Supplementary material for: Brain Expression Genome-Wide Association Study (eGWAS) Identifies Human Disease-Associated Variants
Source: PLoS Genet. 2012 Jun 7;8(6):e1002707. doi: 10.1371/journal.pgen.1002707 (PMC3369937; doi:10.1371/journal.pgen.1002707)
Supplement: Figure S3 — Box Plots of some top cisSNP/transcript associations in the non–AD (a), AD (b) and combined groups (c): The SNP genotypes are shown on the X-axis with the genotype counts in parentheses. Variance stabilizing transformed (VST) expression levels are on the Y-axis. The bottom and top of a box represent the lower and upper quartiles, respectively. The band near the middle of the box is the median. The ends of the whiskers depict the most extreme observations still within 1.5 inter quartile range of the corresponding quartile. Any data not included between the whiskers are plotted as dots. (PDF) [file pgen.1002707.s004.pdf]

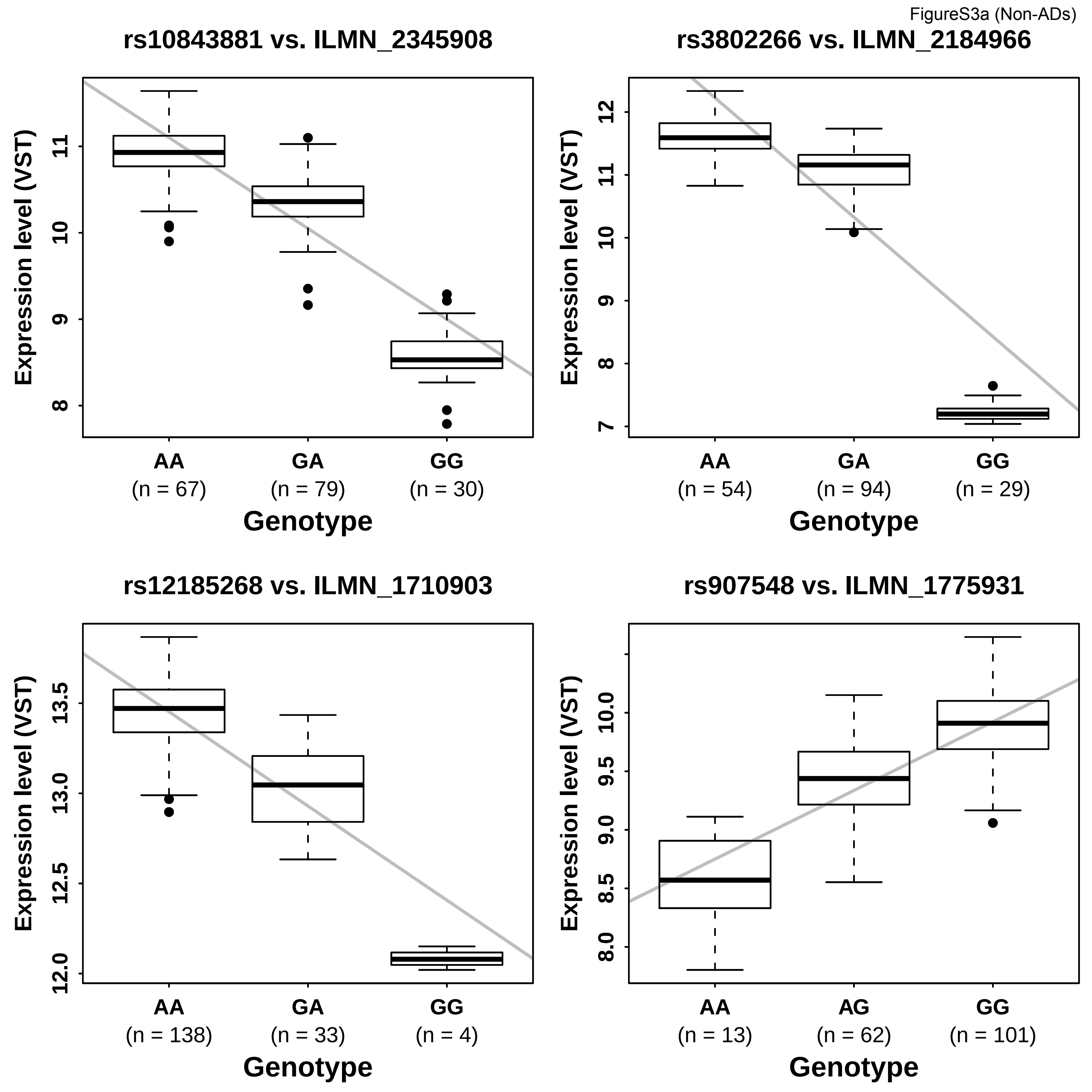
FigureS3a (Non-ADs)
rs10843881 vs. ILMN_2345908
rs3802266 vs. ILMN_2184966
rs12185268 vs. ILMN_1710903
rs907548 vs. ILMN_1775931
Expression level (VST)
Genotype
AA
(n = 67)
GA
(n = 79)
GG
(n = 30)
AA
(n = 54)
GA
(n = 94)
GG
(n = 29)
AA
(n = 138)
GA
(n = 33)
GG
(n = 4)
AA
(n = 13)
AG
(n = 62)
GG
(n = 101)

FigureS3a (Non-ADs)

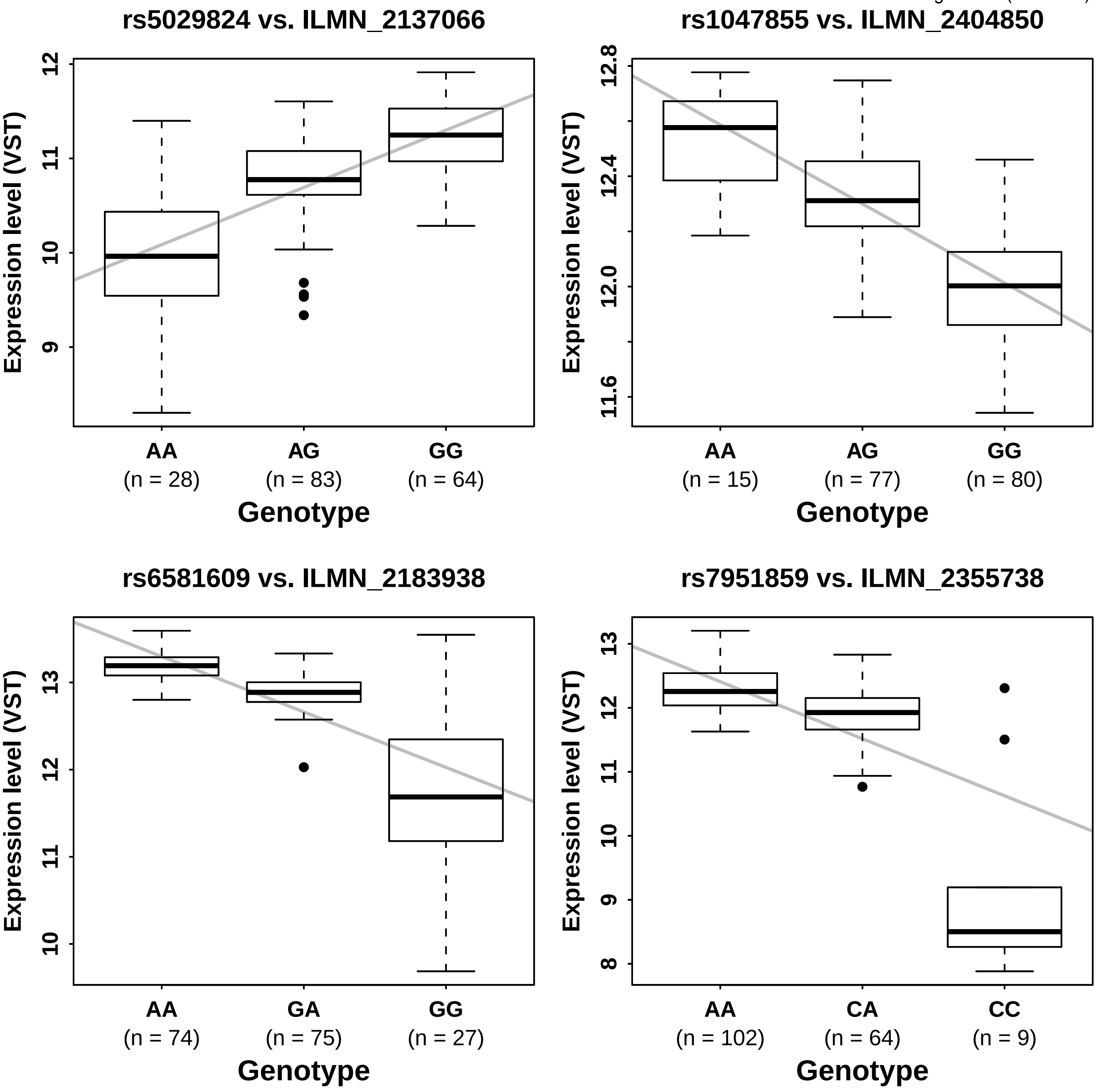

FigureS3a (Non-ADs)

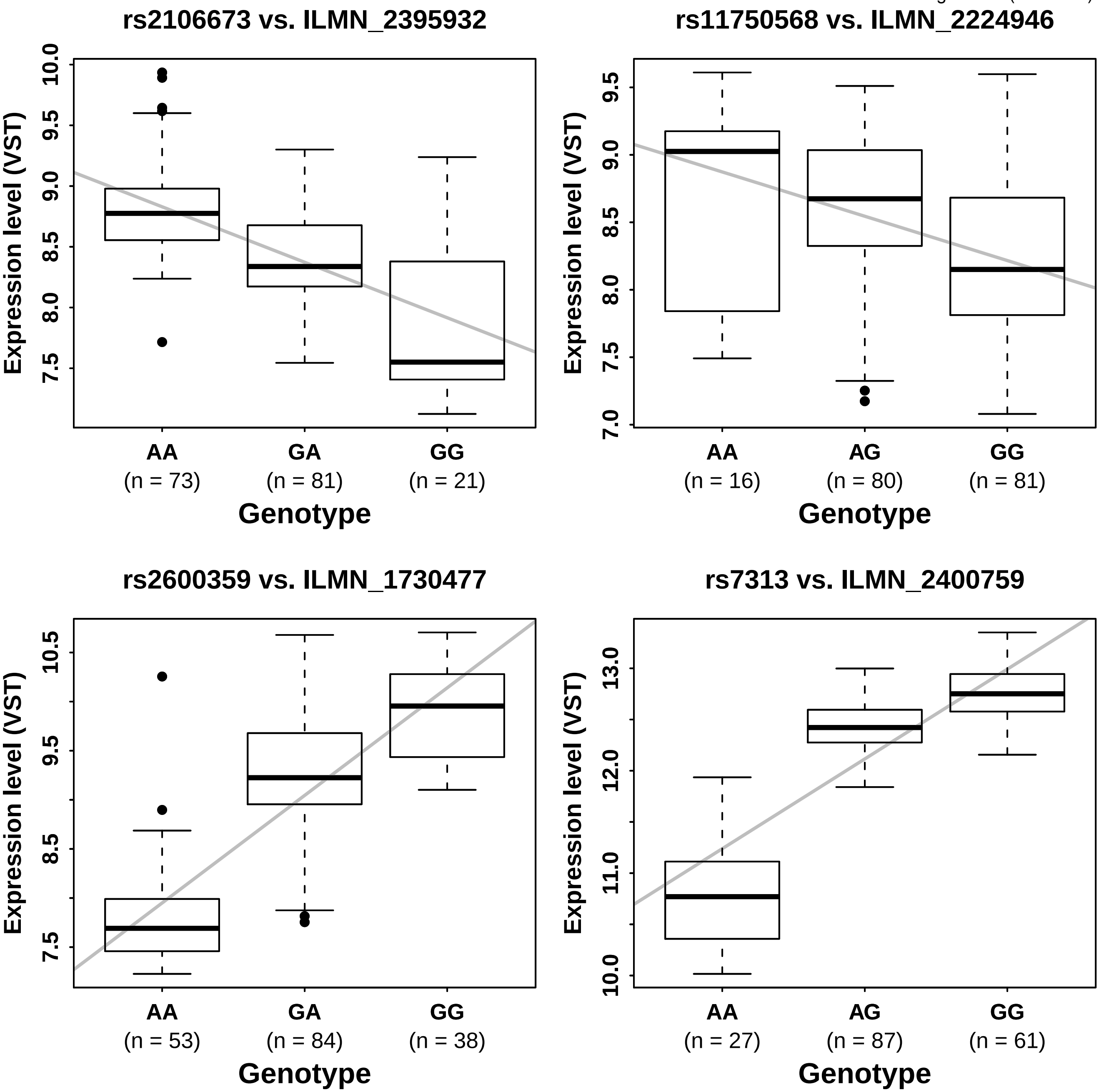

FigureS3a (Non-ADs)

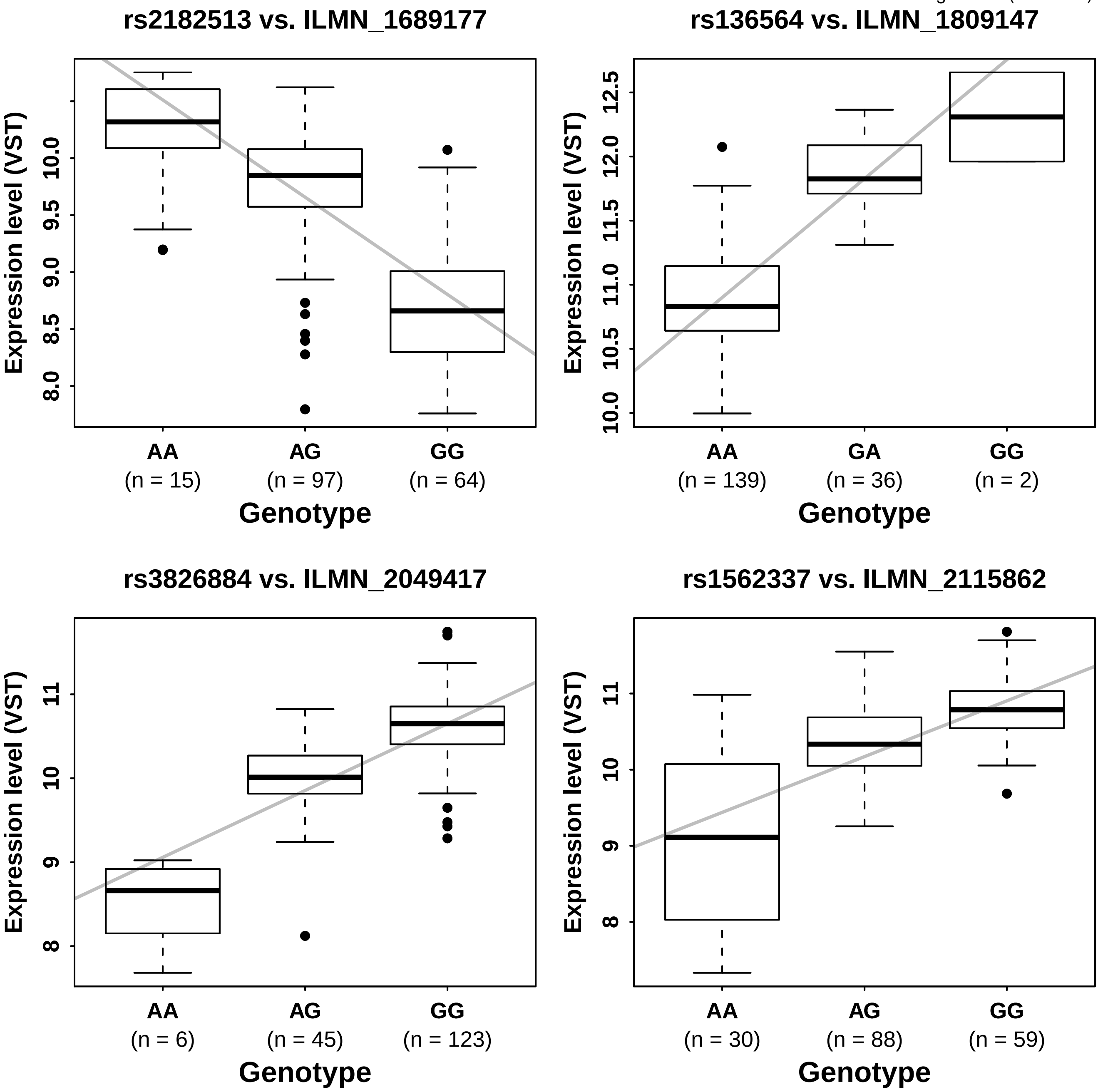

FigureS3a (Non-ADs)

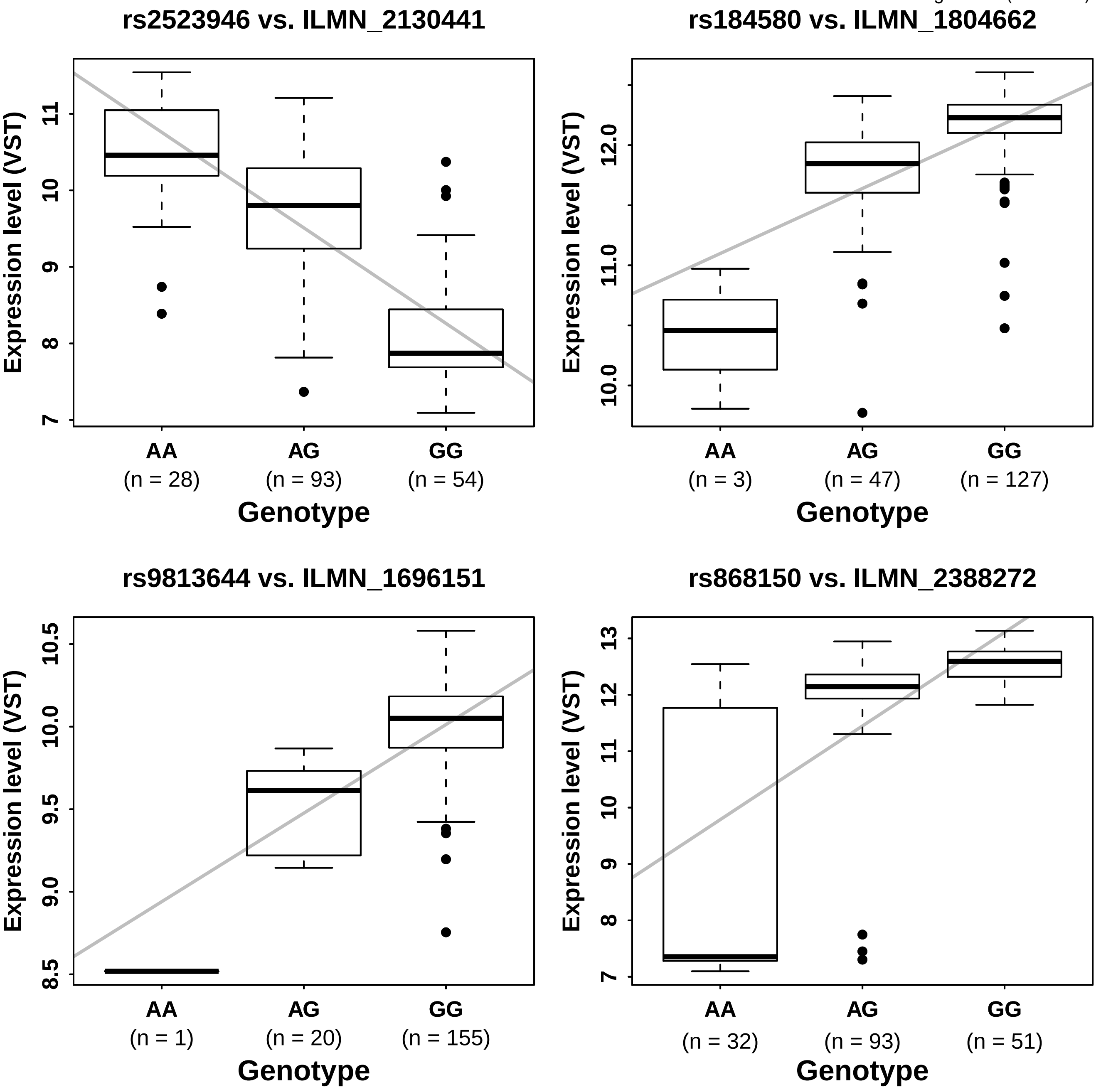

FigureS3a (Non-ADs)

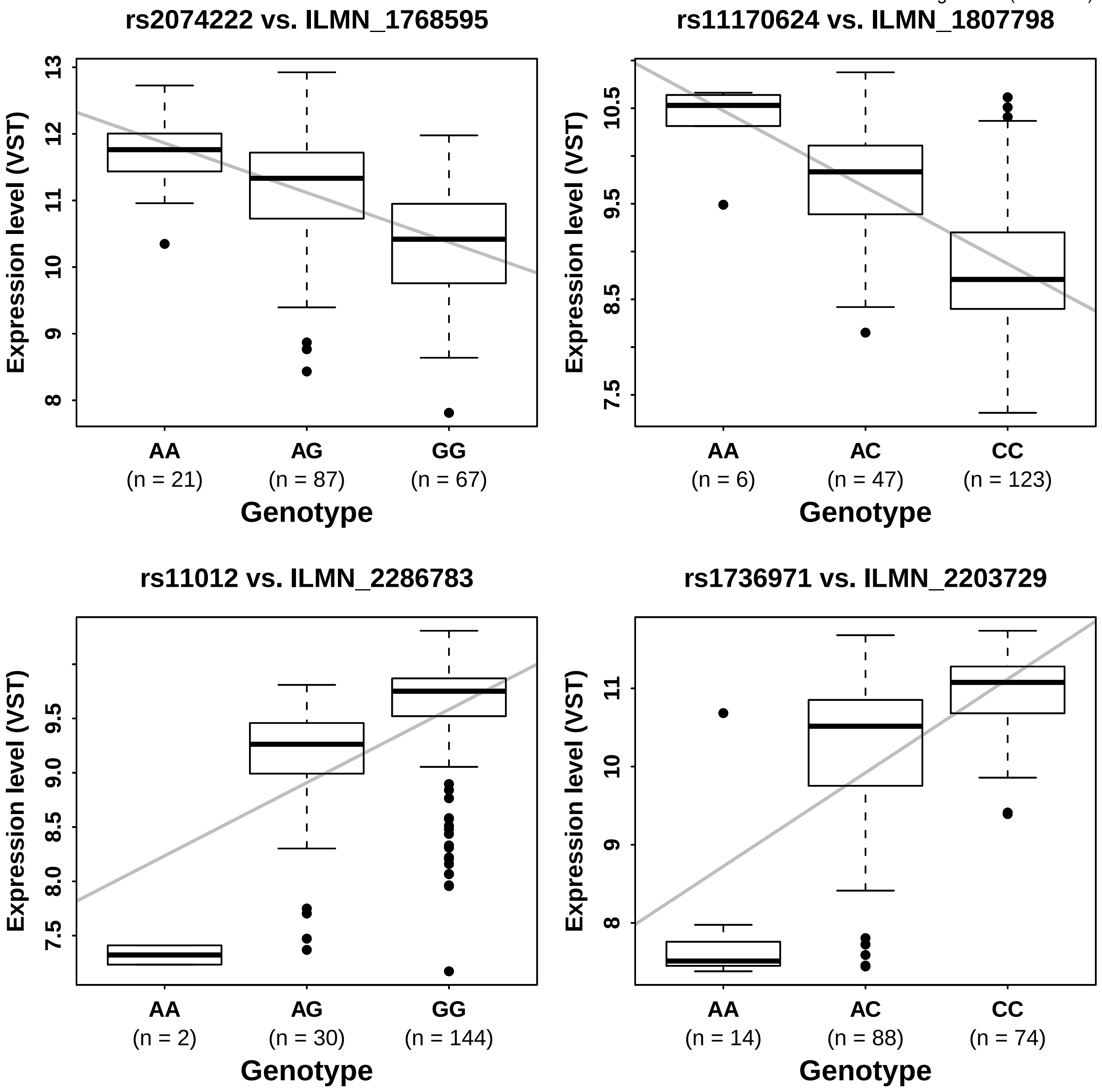

FigureS3a (Non-ADs)

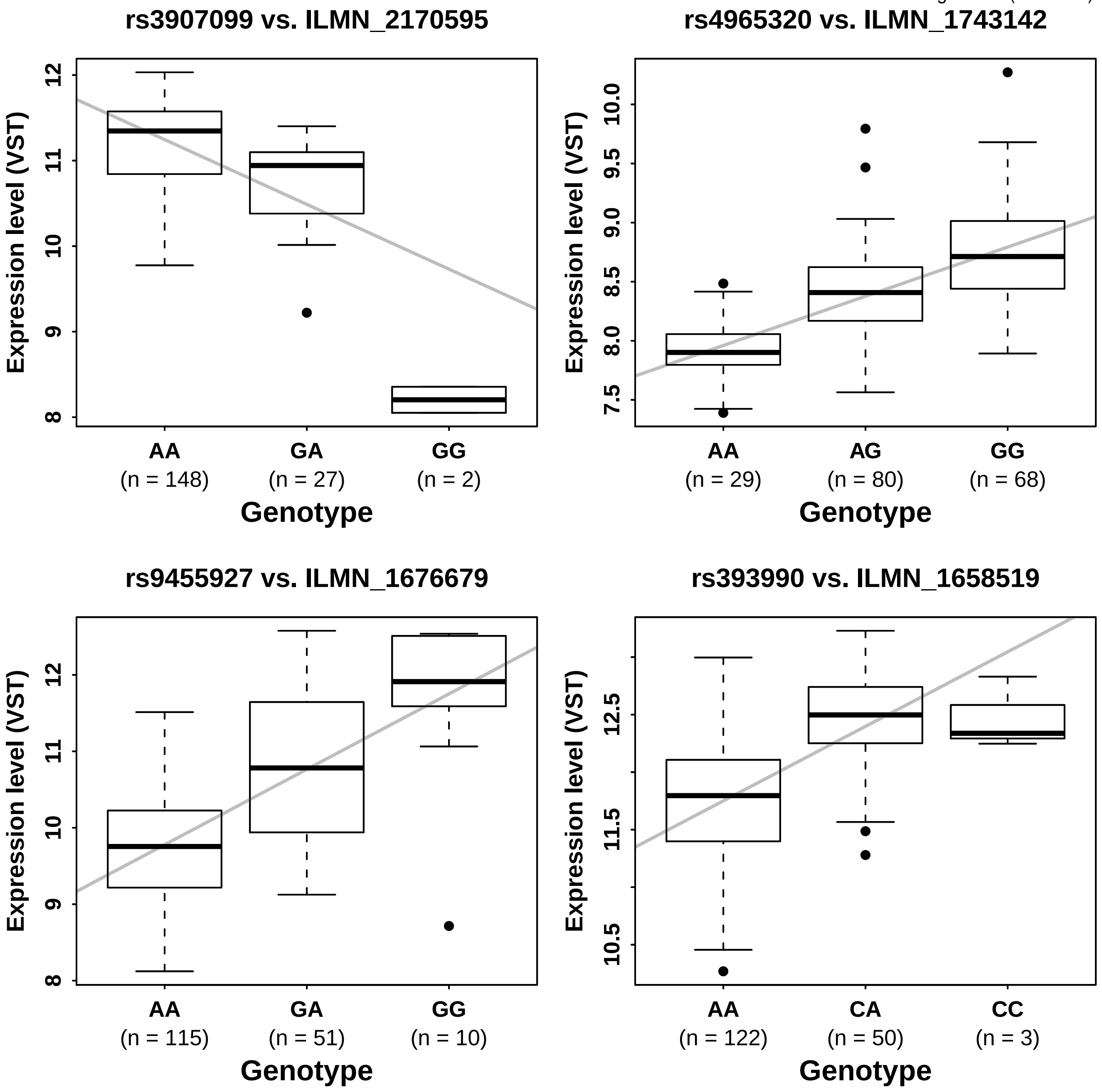

FigureS3a (Non-ADs)

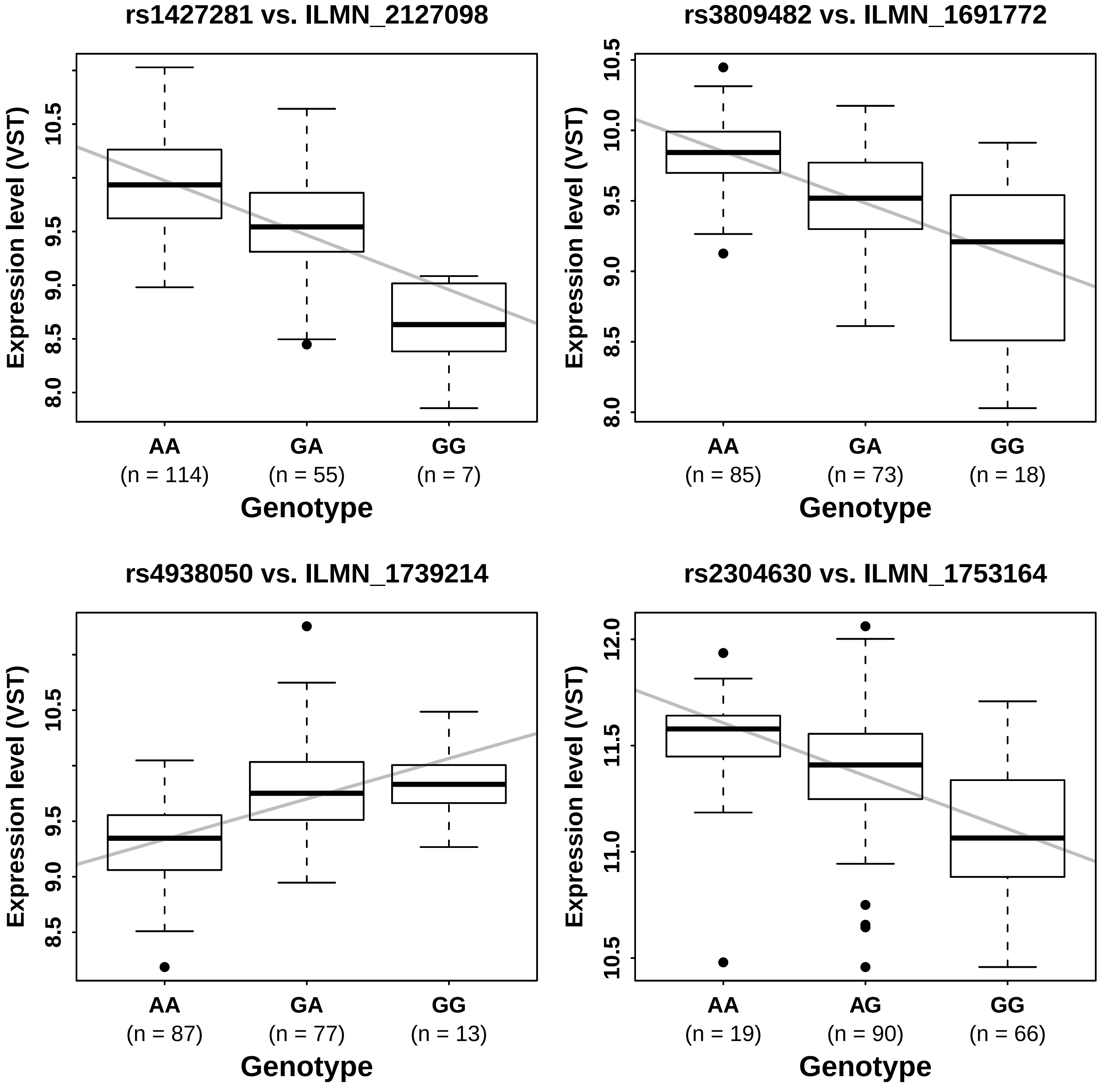

FigureS3a (Non-ADs)

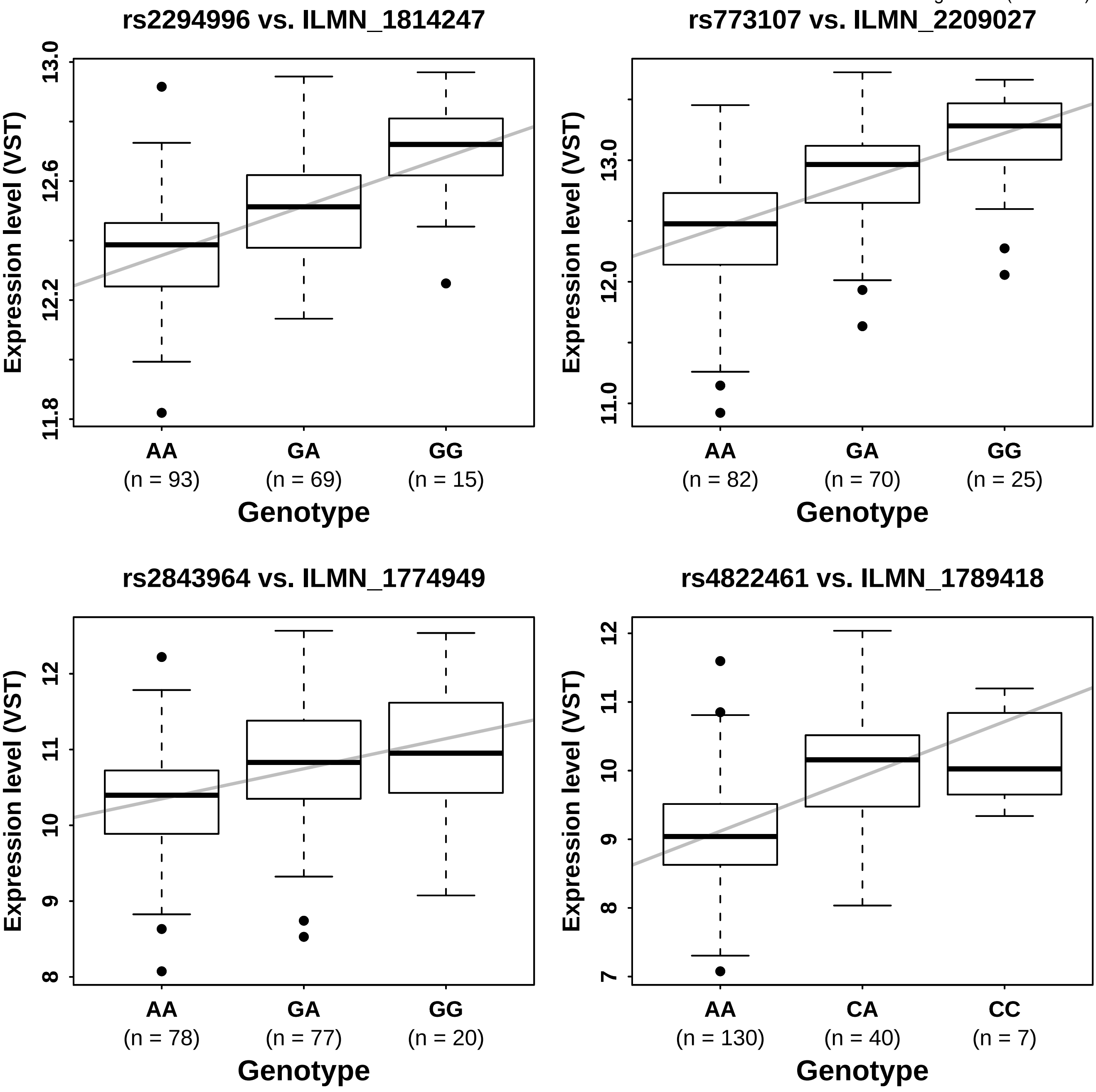

FigureS3a (Non-ADs)

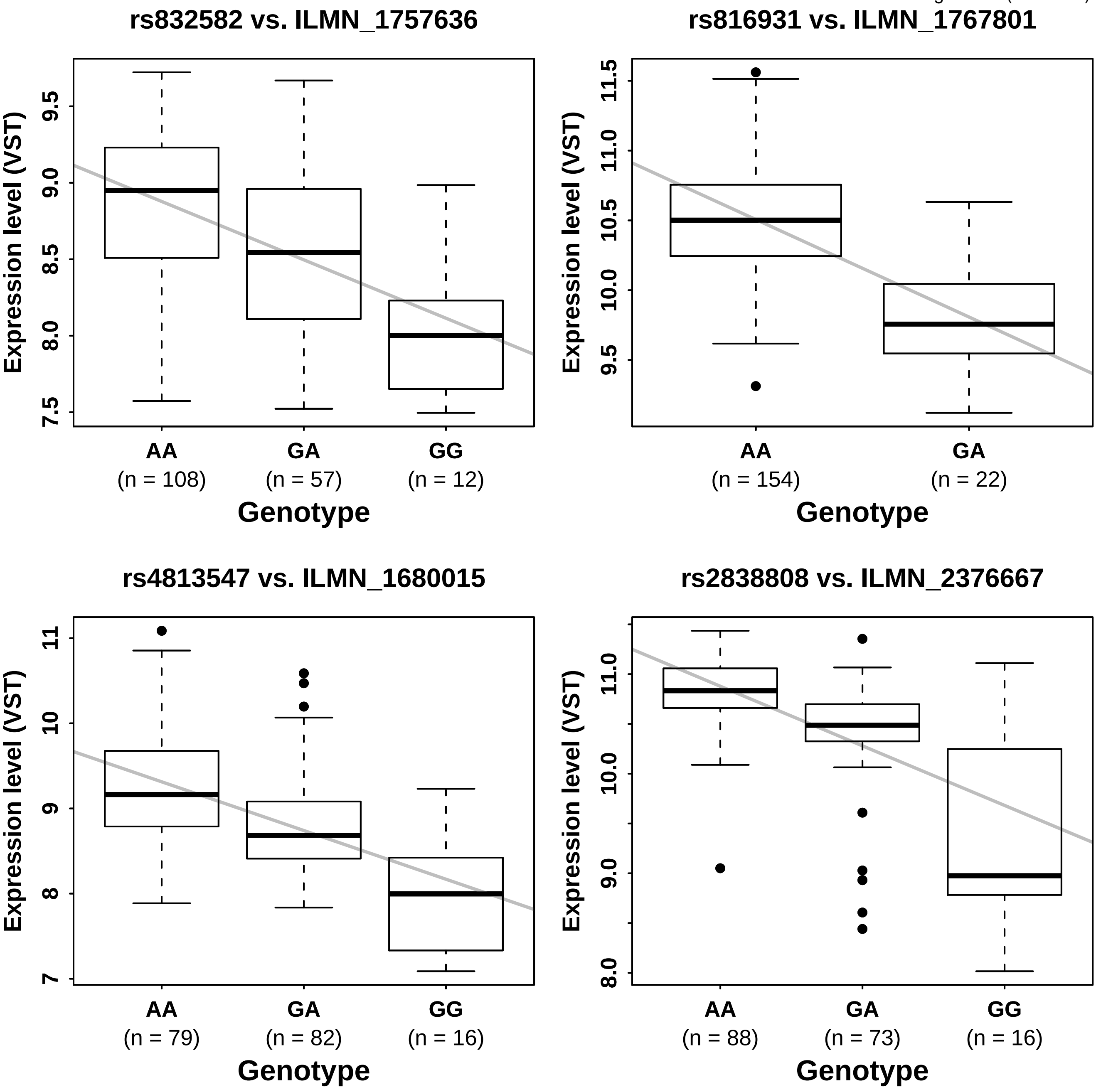

FigureS3a (Non-ADs)

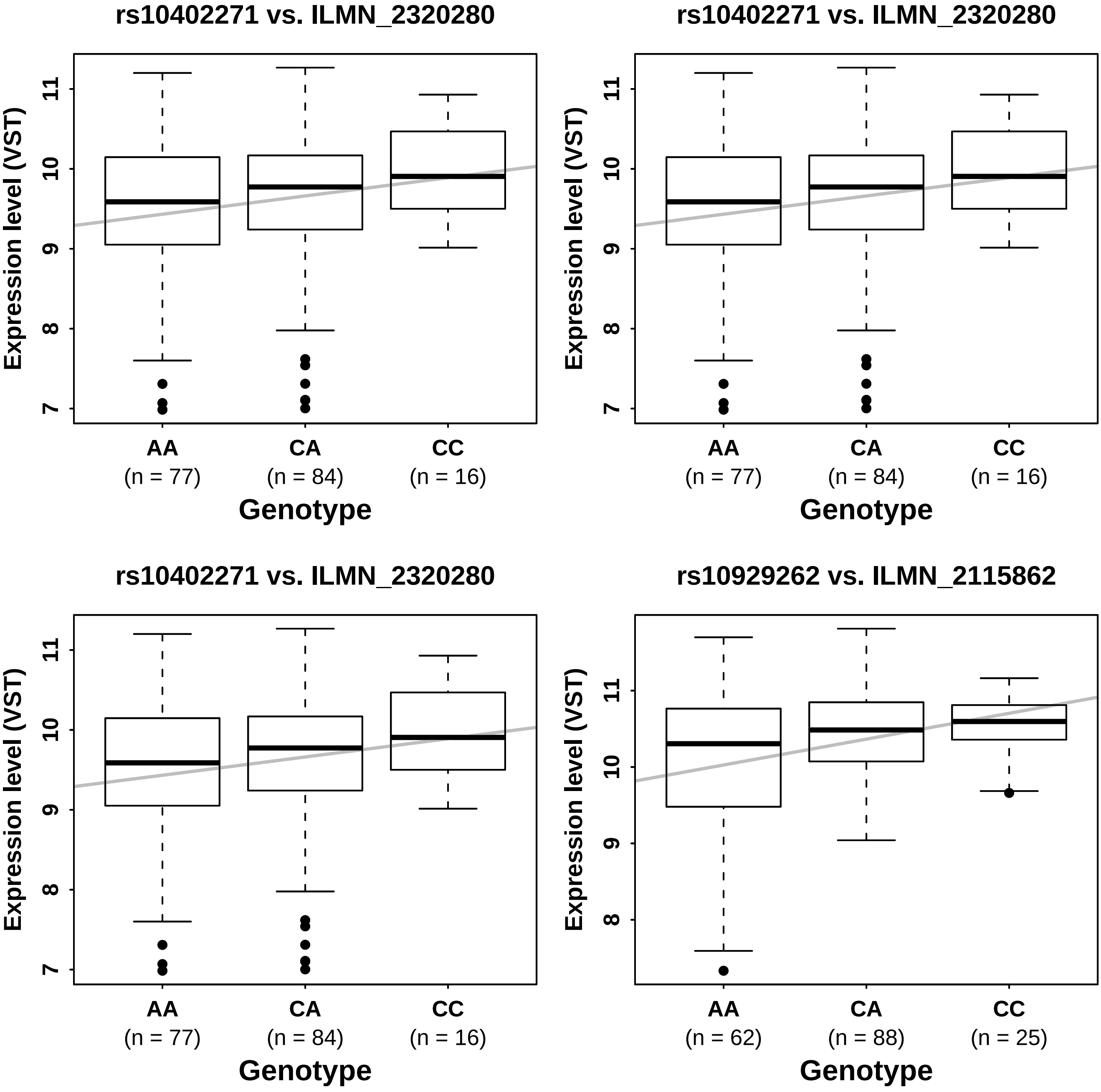

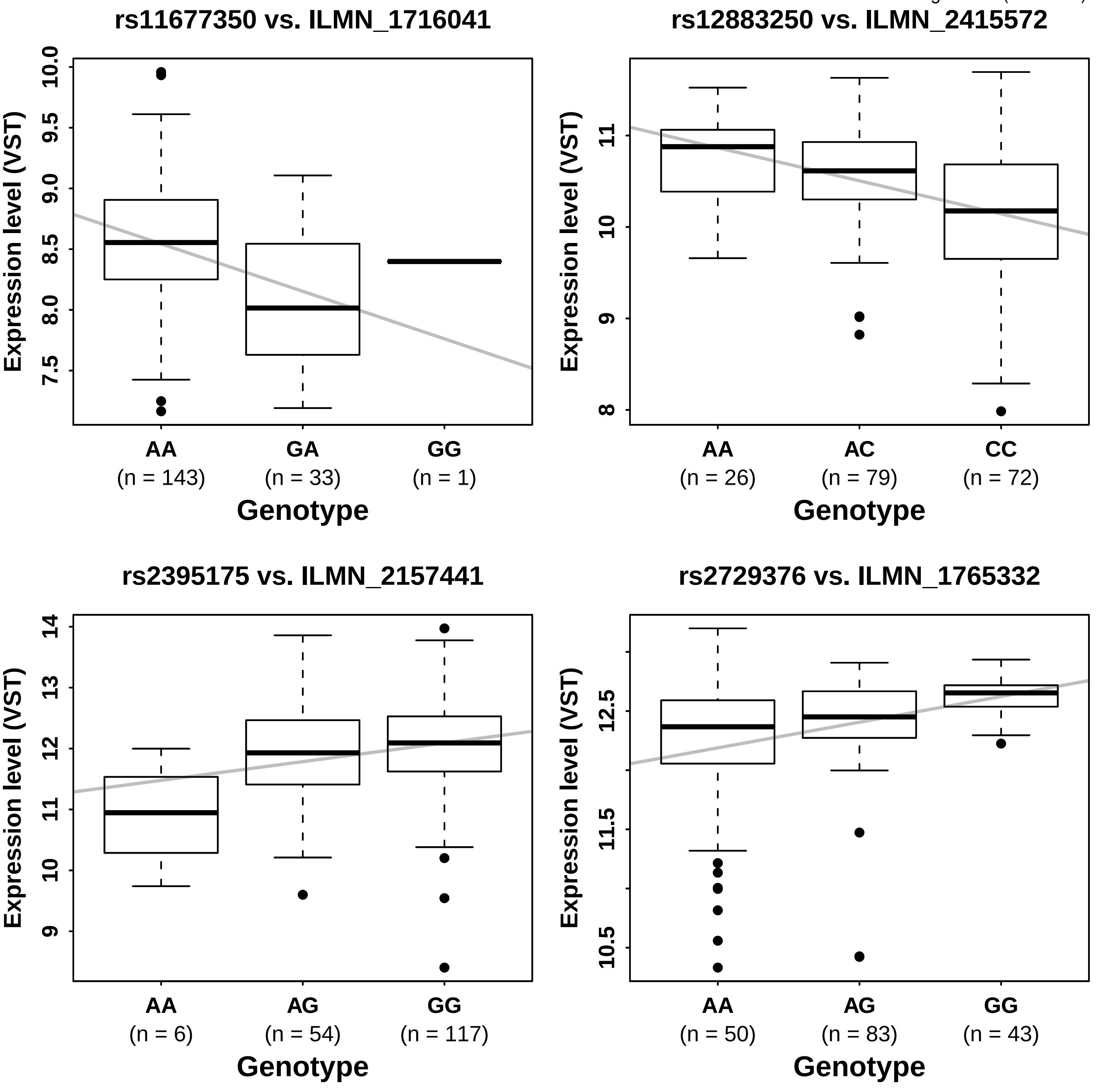
rs11677350 vs. ILMN_1716041
Expression level (VST)
10.0
9.5
9.0
8.5
8.0
7.5
AA
(n = 143)
GA
(n = 33)
GG
(n = 1)
Genotype
rs12883250 vs. ILMN_2415572
Expression level (VST)
11
10
9
8
AA
(n = 26)
AC
(n = 79)
CC
(n = 72)
Genotype
rs2395175 vs. ILMN_2157441
Expression level (VST)
14
13
12
11
10
9
AA
(n = 6)
AG
(n = 54)
GG
(n = 117)
Genotype
rs2729376 vs. ILMN_1765332
Expression level (VST)
12.5
11.5
10.5
AA
(n = 50)
AG
(n = 83)
GG
(n = 43)
Genotype

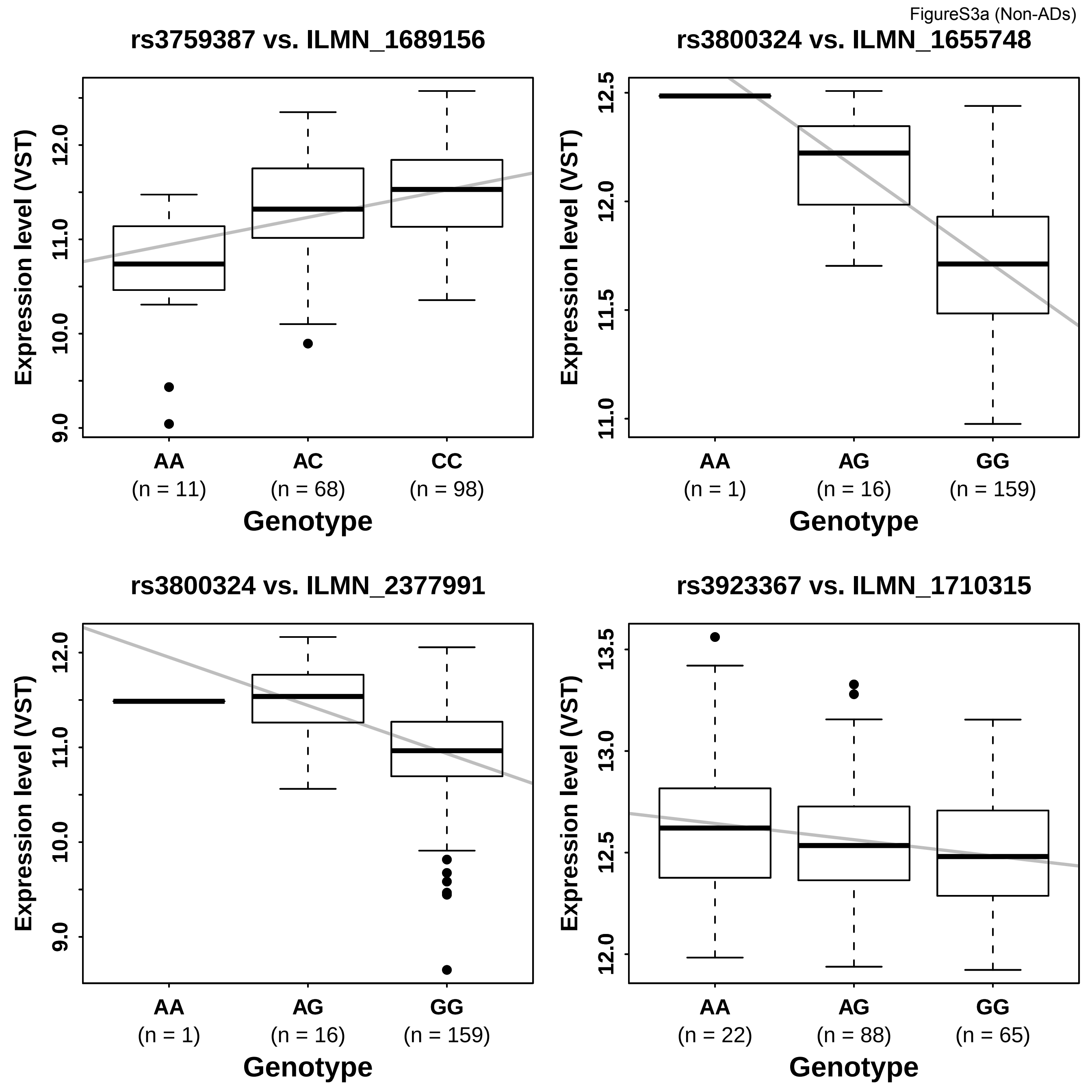
FigureS3a (Non-ADs)
rs3759387 vs. ILMN_1689156
Expression level (VST)
9.0
10.0
11.0
12.0
AA
(n = 11)
AC
(n = 68)
CC
(n = 98)
Genotype
rs3800324 vs. ILMN_1655748
Expression level (VST)
11.0
11.5
12.0
12.5
AA
(n = 1)
AG
(n = 16)
GG
(n = 159)
Genotype
rs3800324 vs. ILMN_2377991
Expression level (VST)
9.0
10.0
11.0
12.0
AA
(n = 1)
AG
(n = 16)
GG
(n = 159)
Genotype
rs3923367 vs. ILMN_1710315
Expression level (VST)
12.0
12.5
13.0
13.5
AA
(n = 22)
AG
(n = 88)
GG
(n = 65)
Genotype

FigureS3a (Non-ADs)

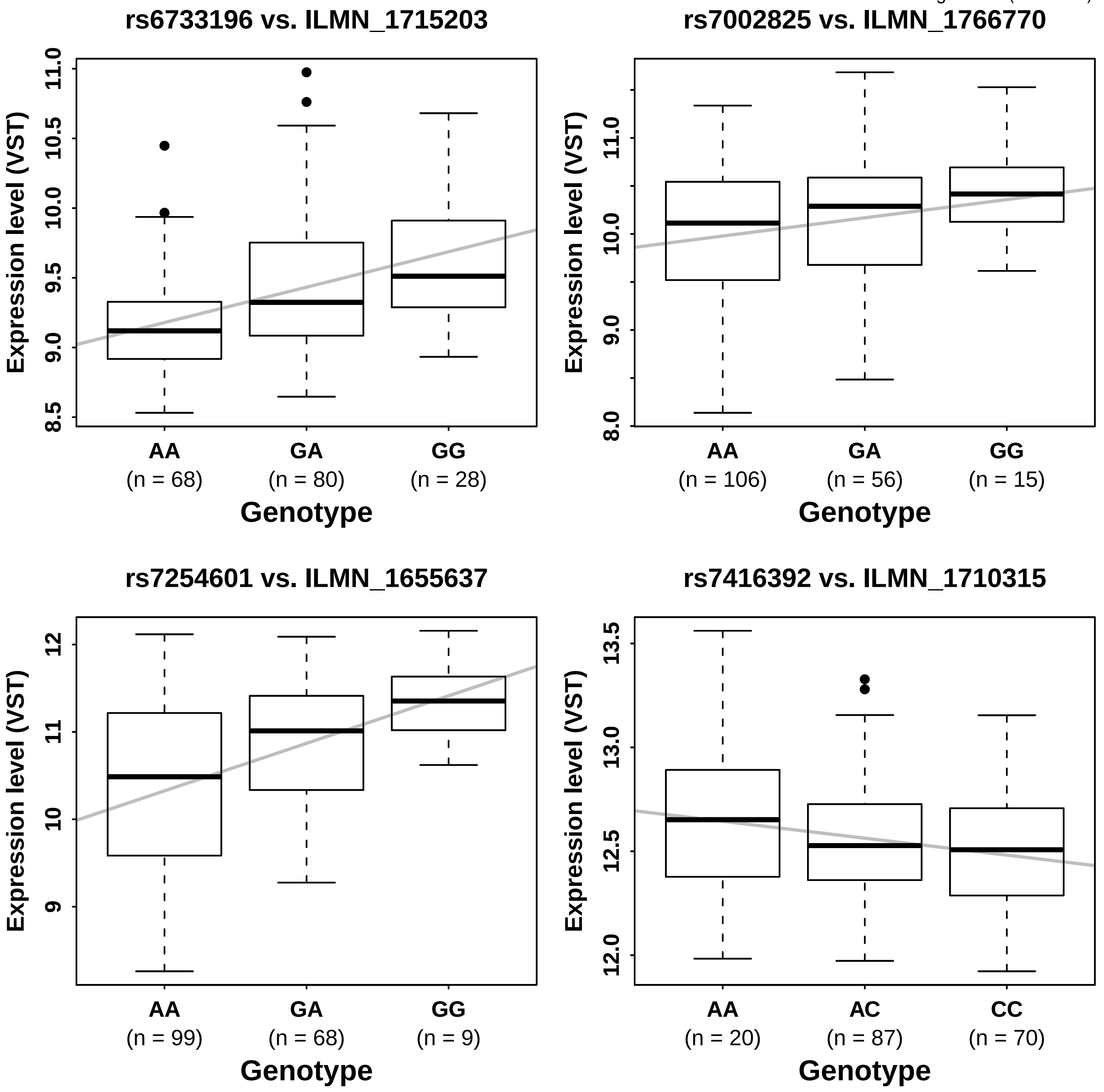

FigureS3a (Non-ADs)

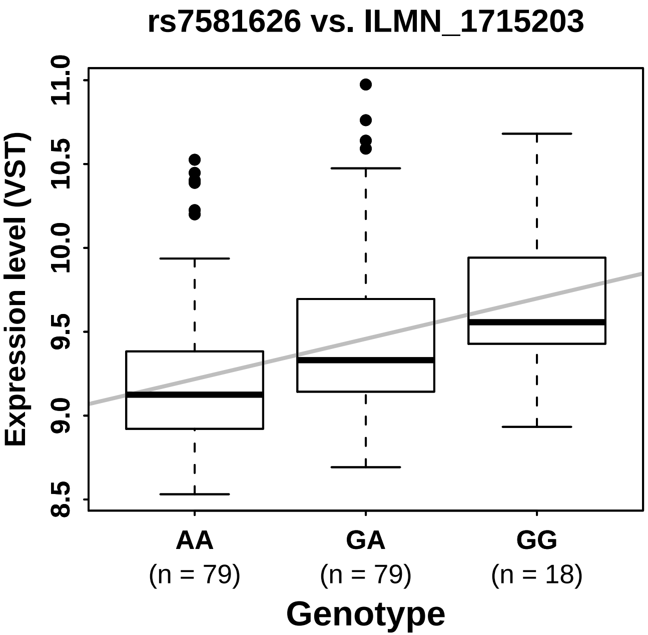

Figure S3b (ADs)

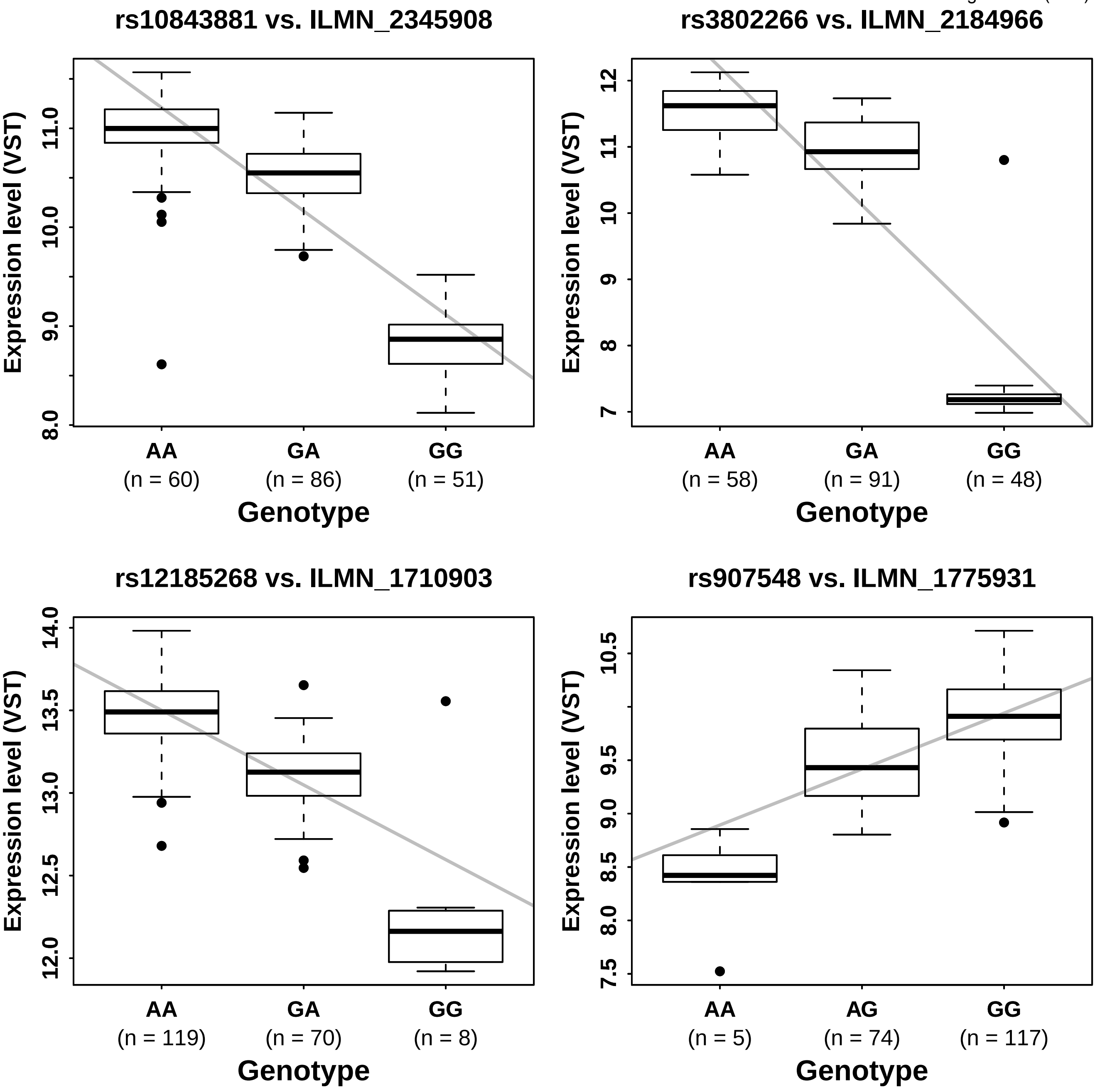

Figure S3b (ADs)

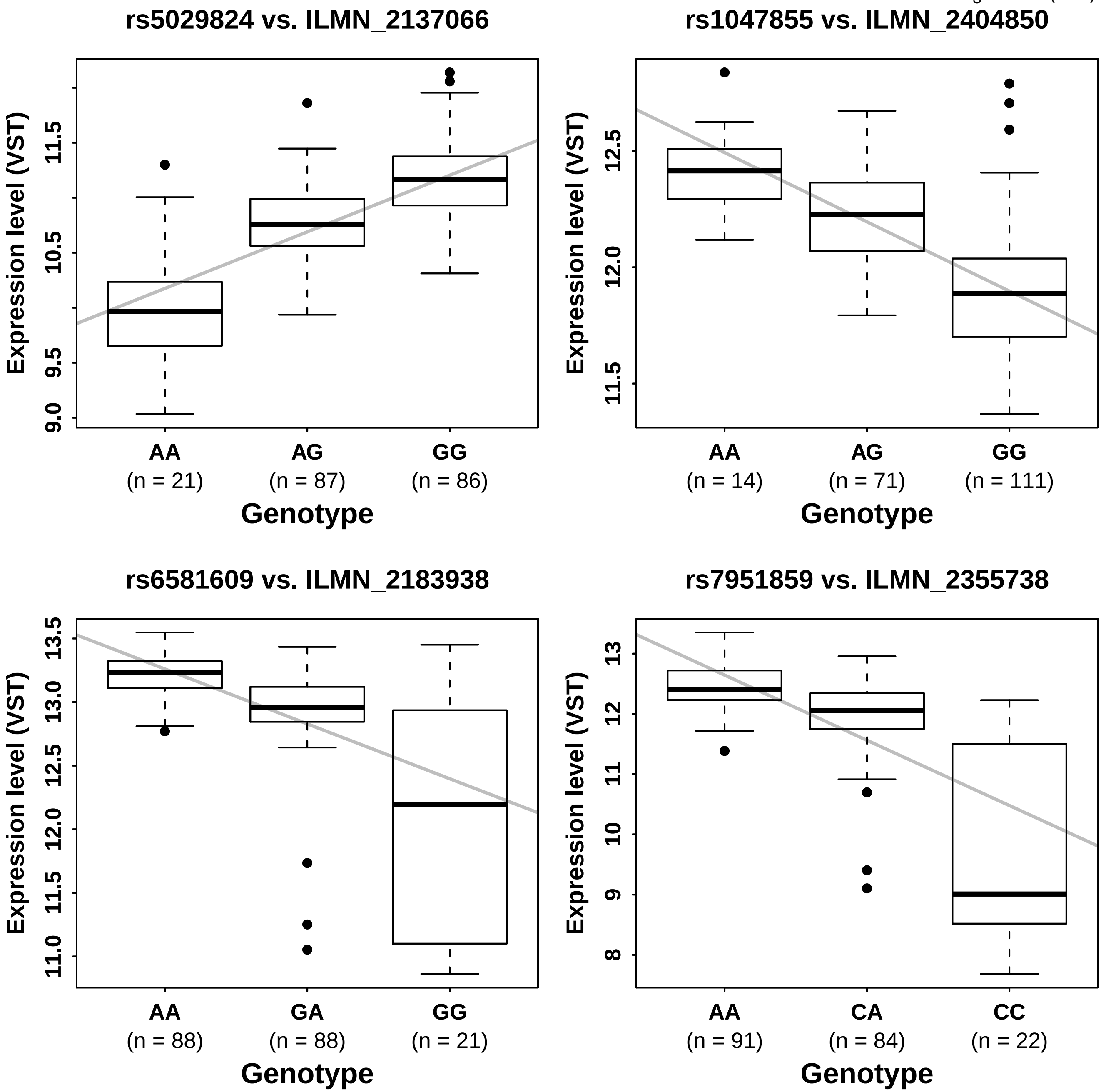

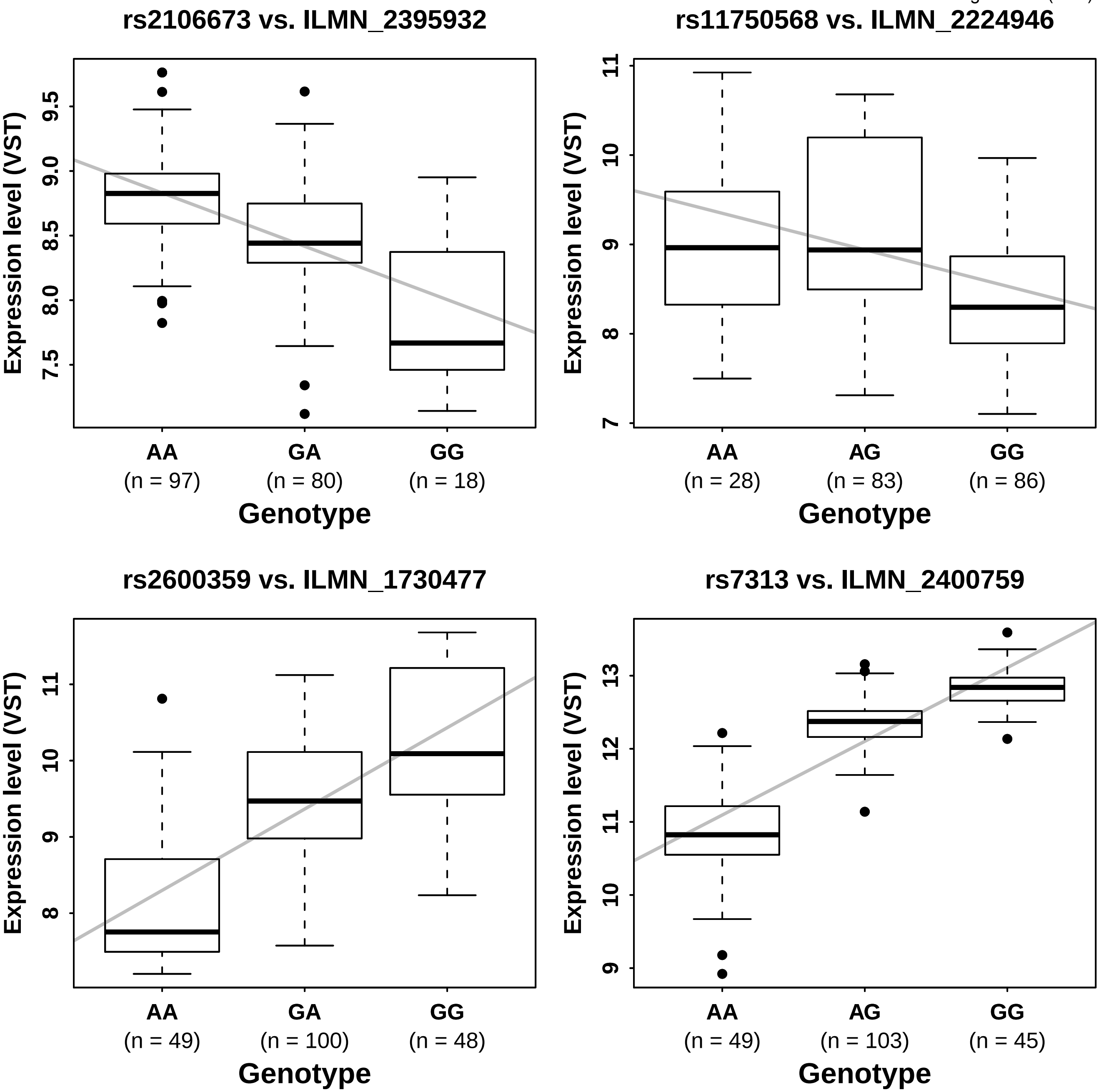
rs2106673 vs. ILMN_2395932
Expression level (VST)
9.5
9.0
8.5
8.0
7.5
AA
(n = 97)
GA
(n = 80)
GG
(n = 18)
Genotype
rs11750568 vs. ILMN_2224946
Expression level (VST)
11
10
9
8
7
AA
(n = 28)
AG
(n = 83)
GG
(n = 86)
Genotype
rs2600359 vs. ILMN_1730477
Expression level (VST)
11
10
9
8
AA
(n = 49)
GA
(n = 100)
GG
(n = 48)
Genotype
rs7313 vs. ILMN_2400759
Expression level (VST)
13
12
11
10
9
AA
(n = 49)
AG
(n = 103)
GG
(n = 45)
Genotype

Figure S3b (ADs)

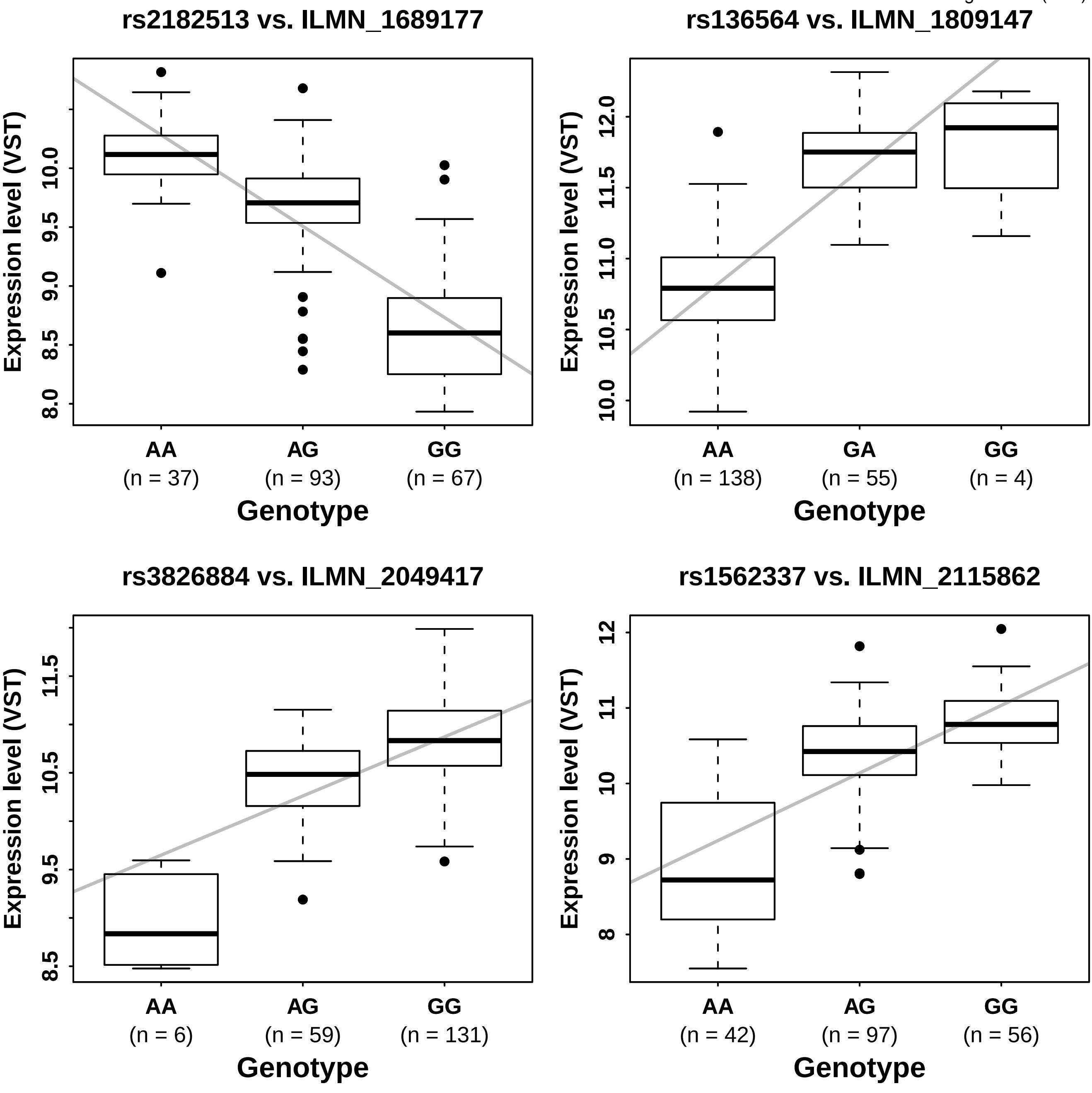

Figure S3b (ADs)

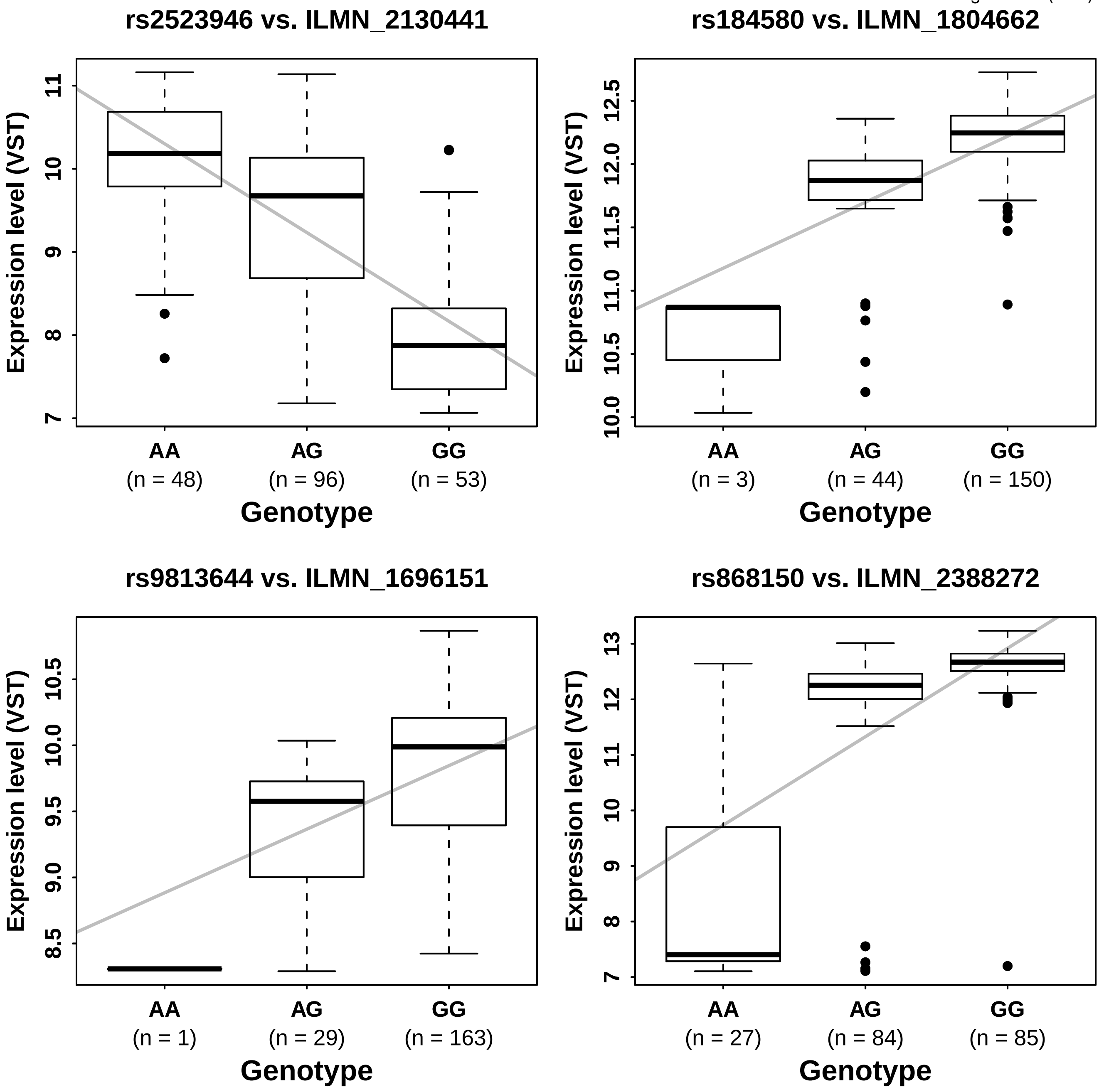

Figure S3b (ADs)

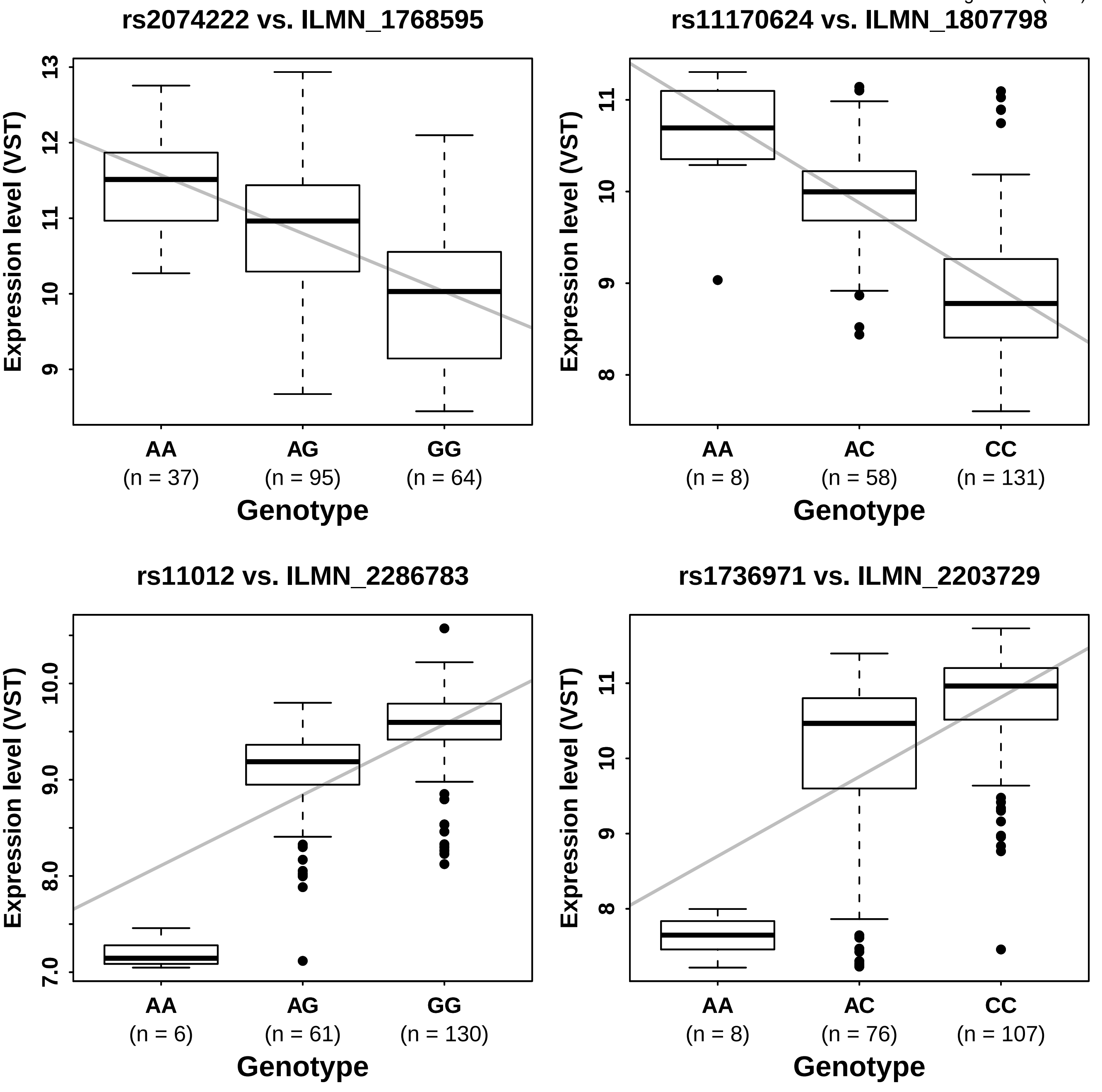

Figure S3b (ADs)

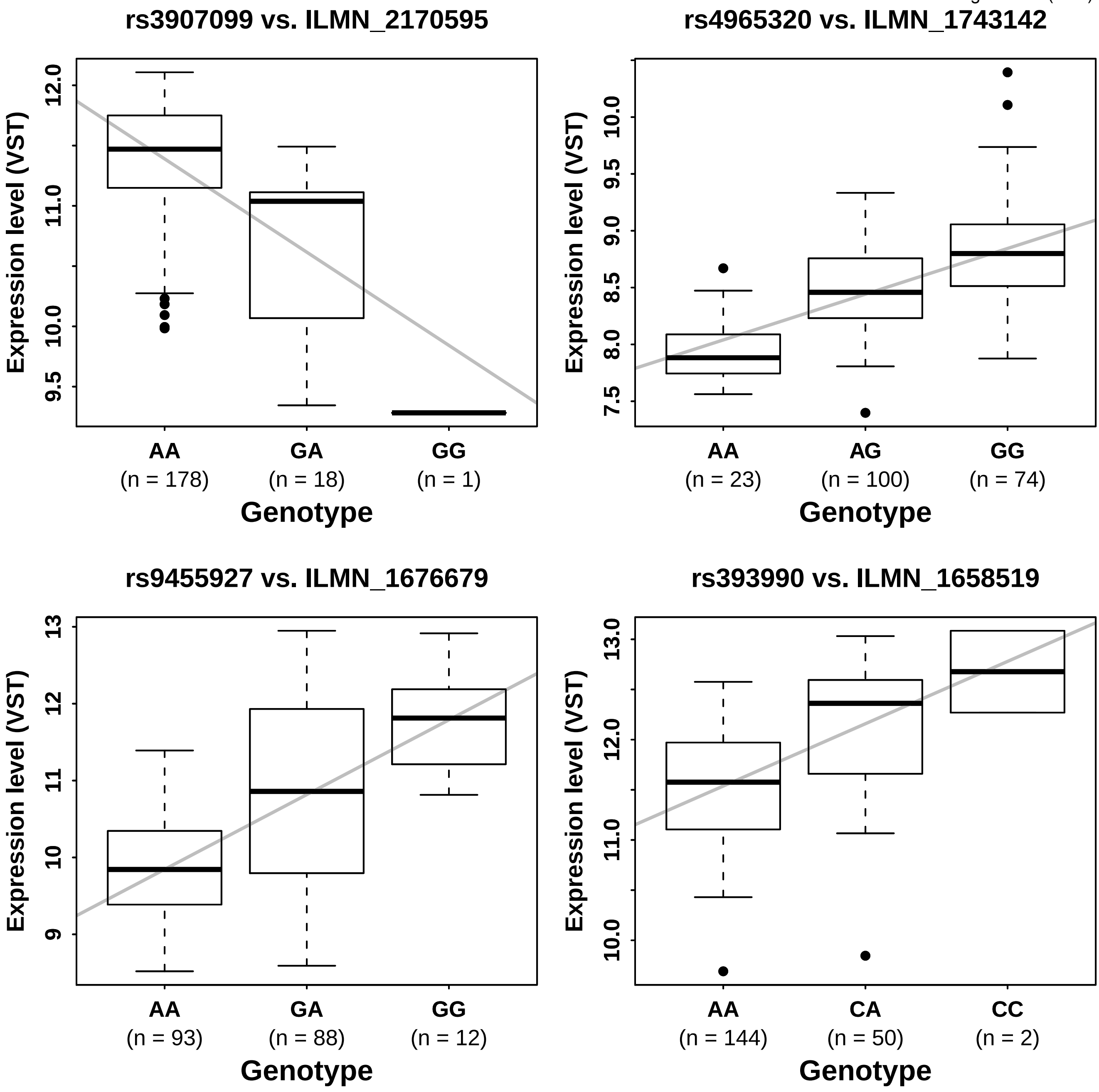

Figure S3b (ADs)

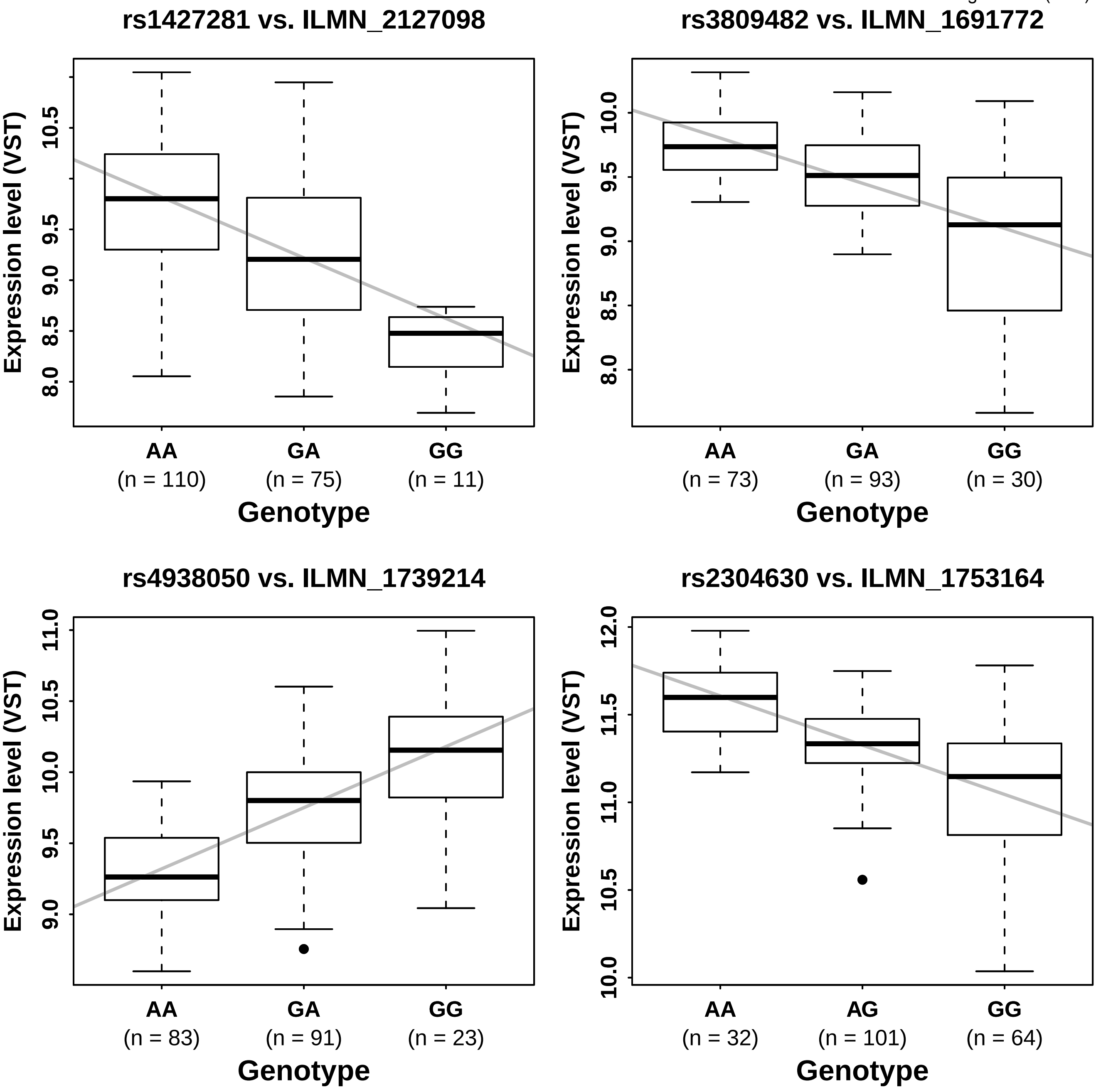

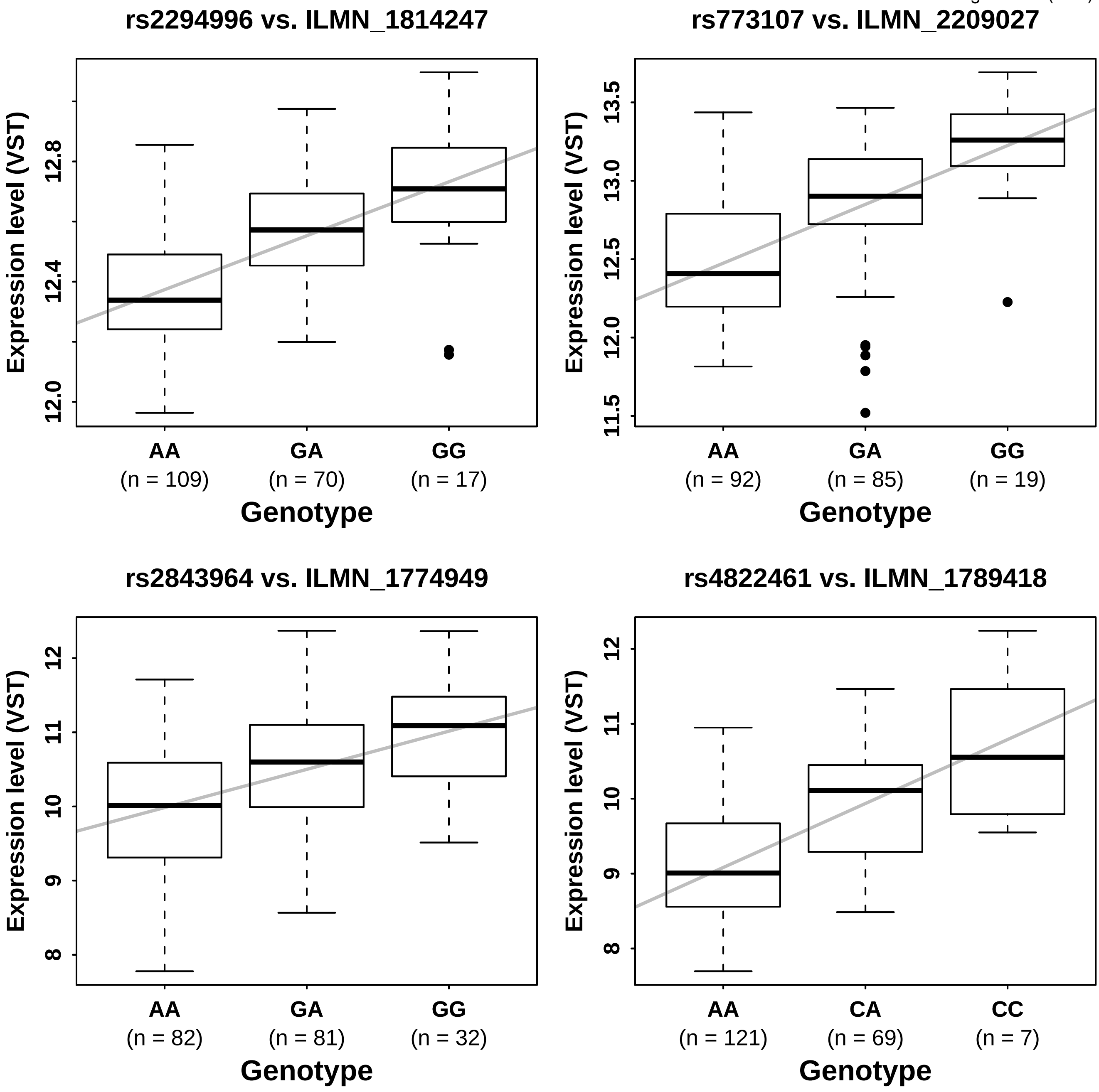
rs2294996 vs. ILMN_1814247
Expression level (VST)
12.0
12.4
12.8
AA
(n = 109)
GA
(n = 70)
GG
(n = 17)
Genotype
rs773107 vs. ILMN_2209027
Expression level (VST)
11.5
12.0
12.5
13.0
13.5
AA
(n = 92)
GA
(n = 85)
GG
(n = 19)
Genotype
rs2843964 vs. ILMN_1774949
Expression level (VST)
8
9
10
11
12
AA
(n = 82)
GA
(n = 81)
GG
(n = 32)
Genotype
rs4822461 vs. ILMN_1789418
Expression level (VST)
8
9
10
11
12
AA
(n = 121)
CA
(n = 69)
CC
(n = 7)
Genotype

Figure S3b (ADs)

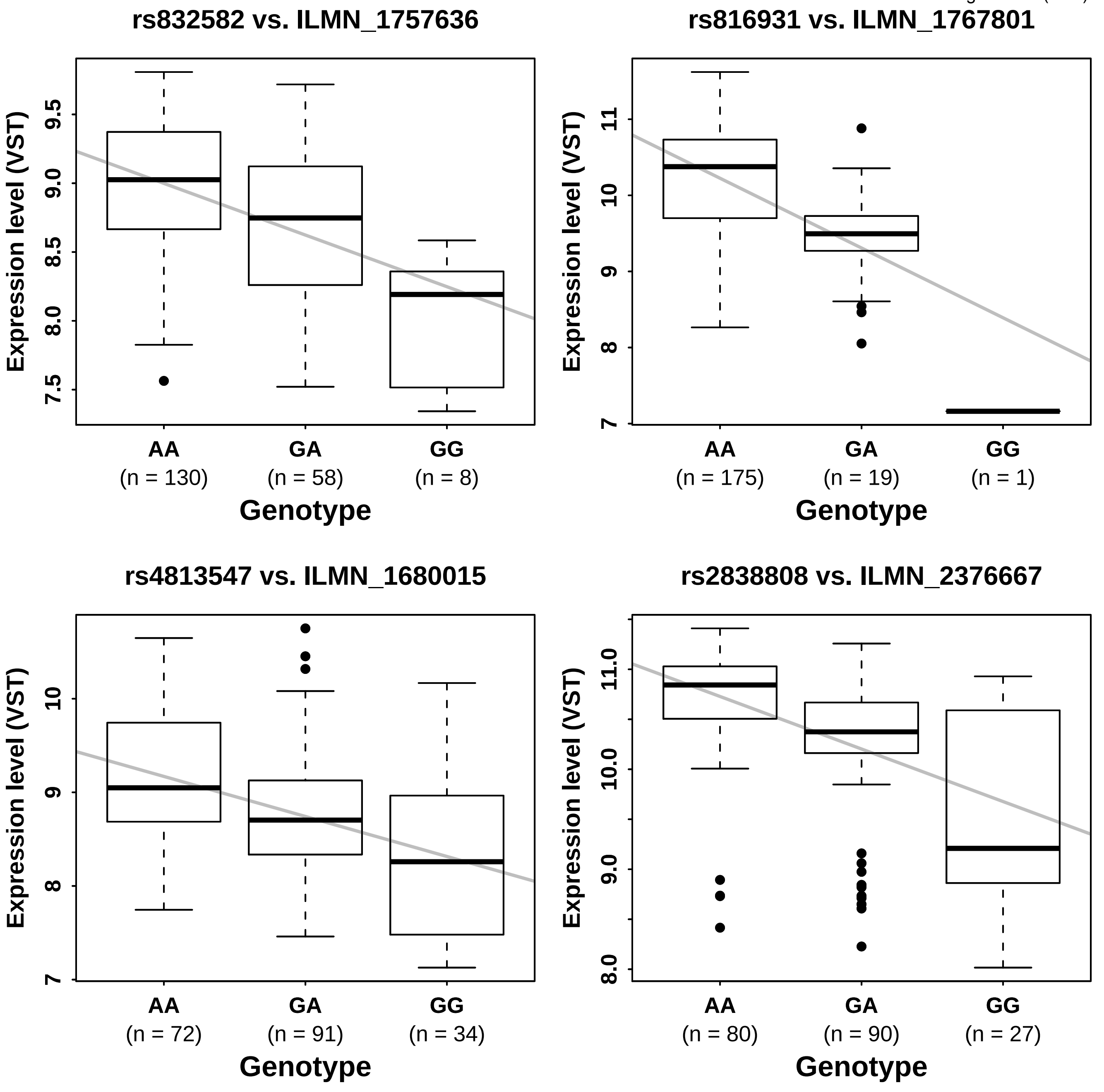

Figure S3b (ADs)

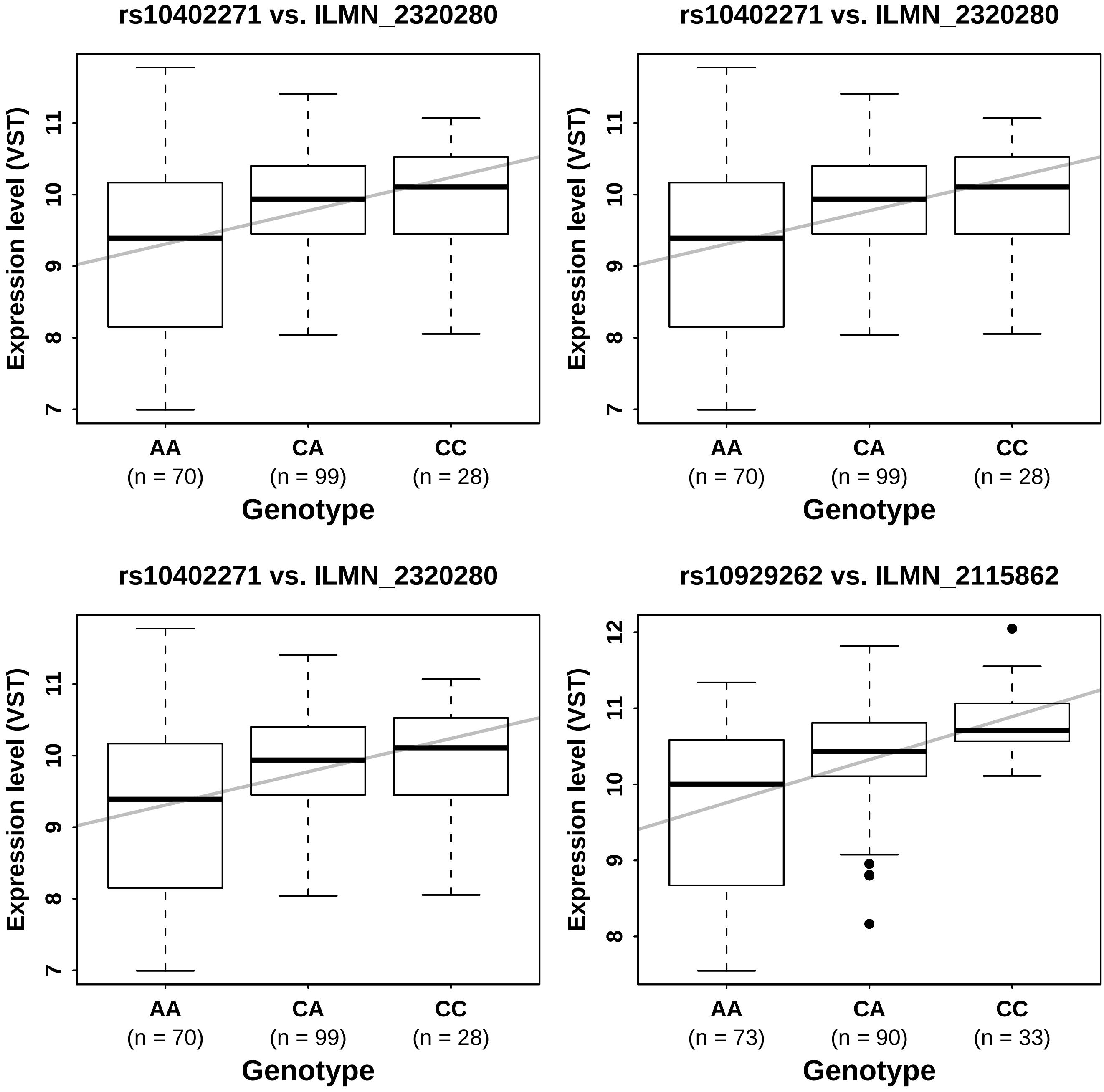

Figure S3b (ADs)

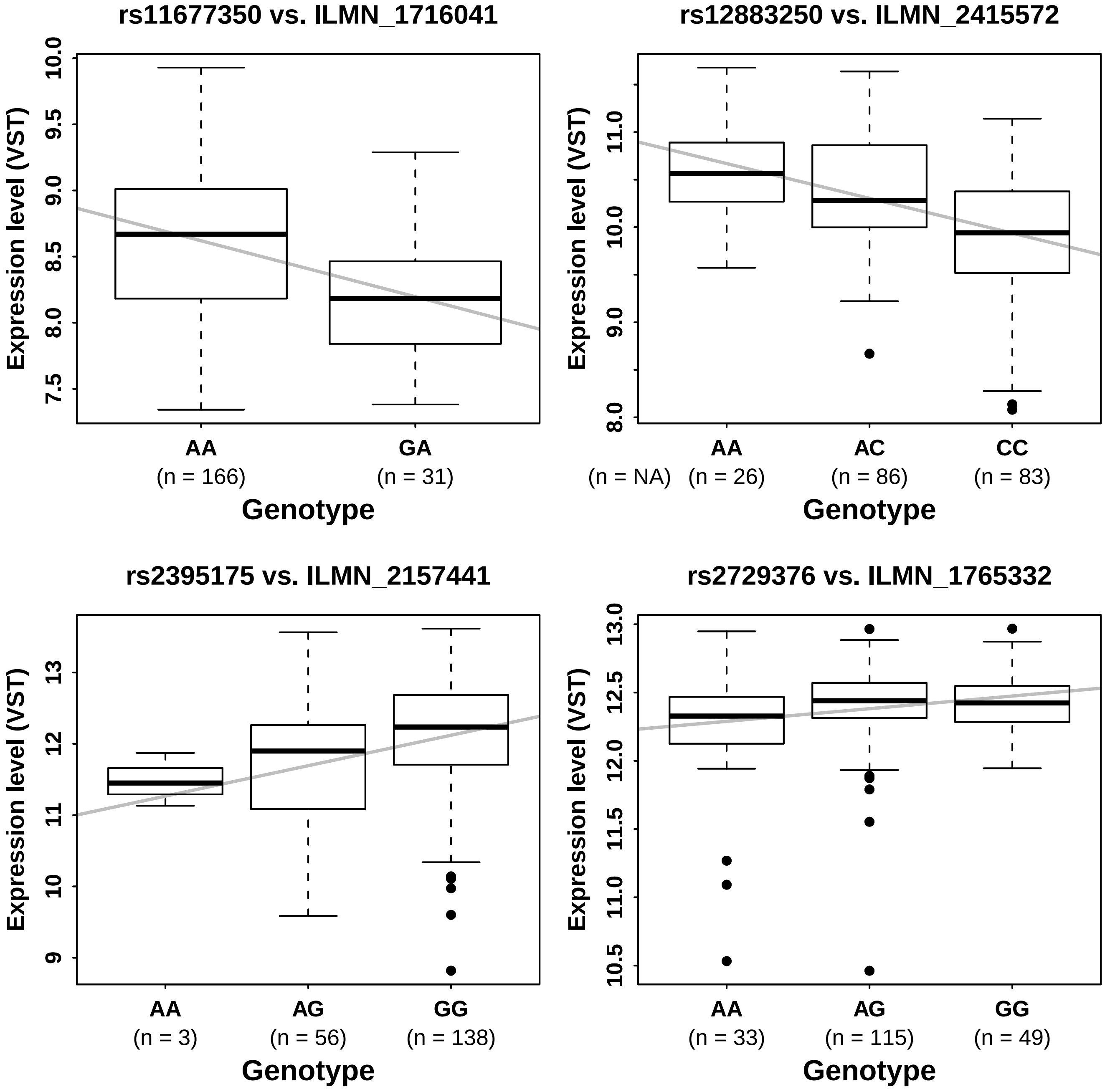

Figure S3b (ADs)

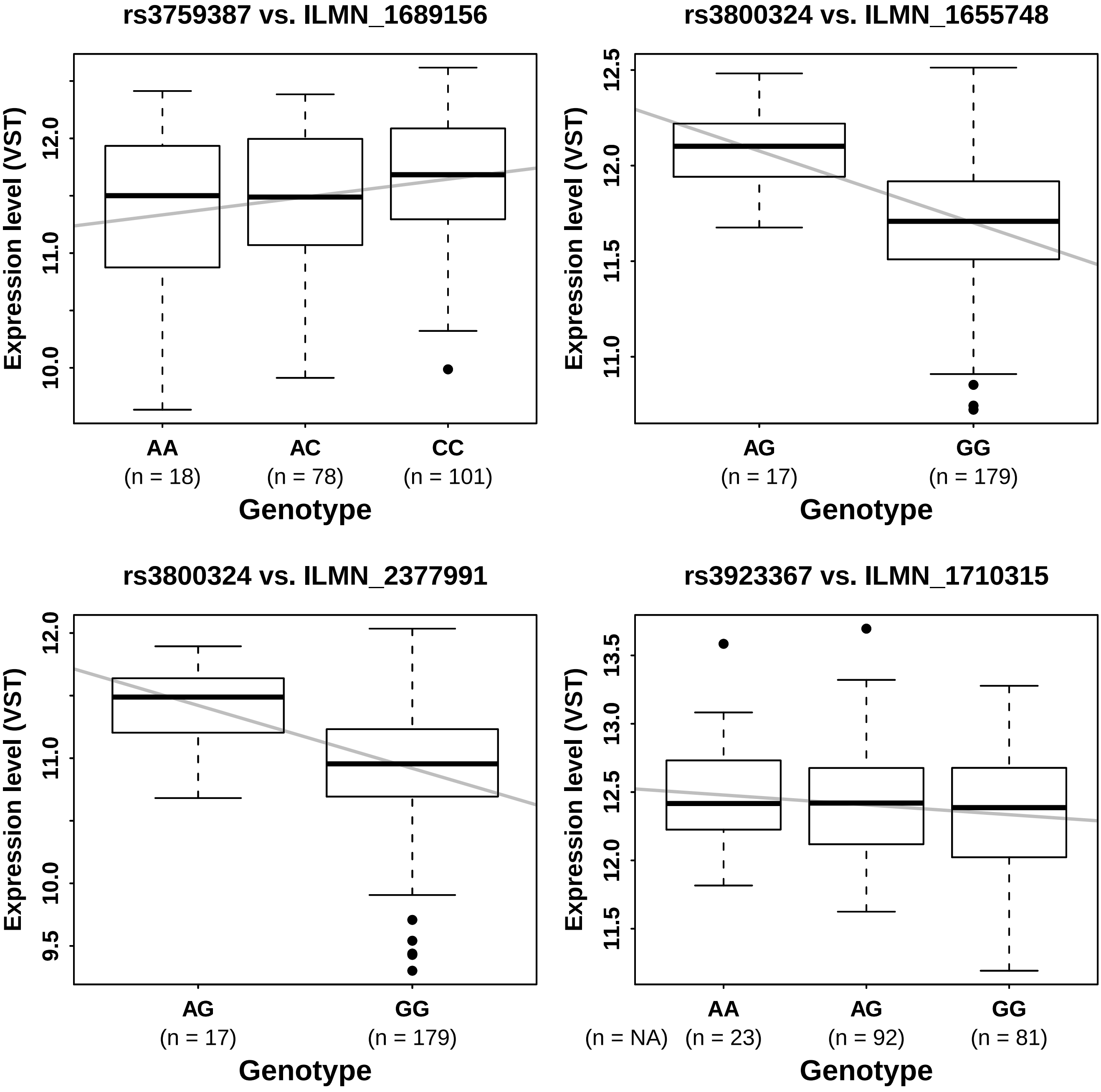

Figure S3b (ADs)

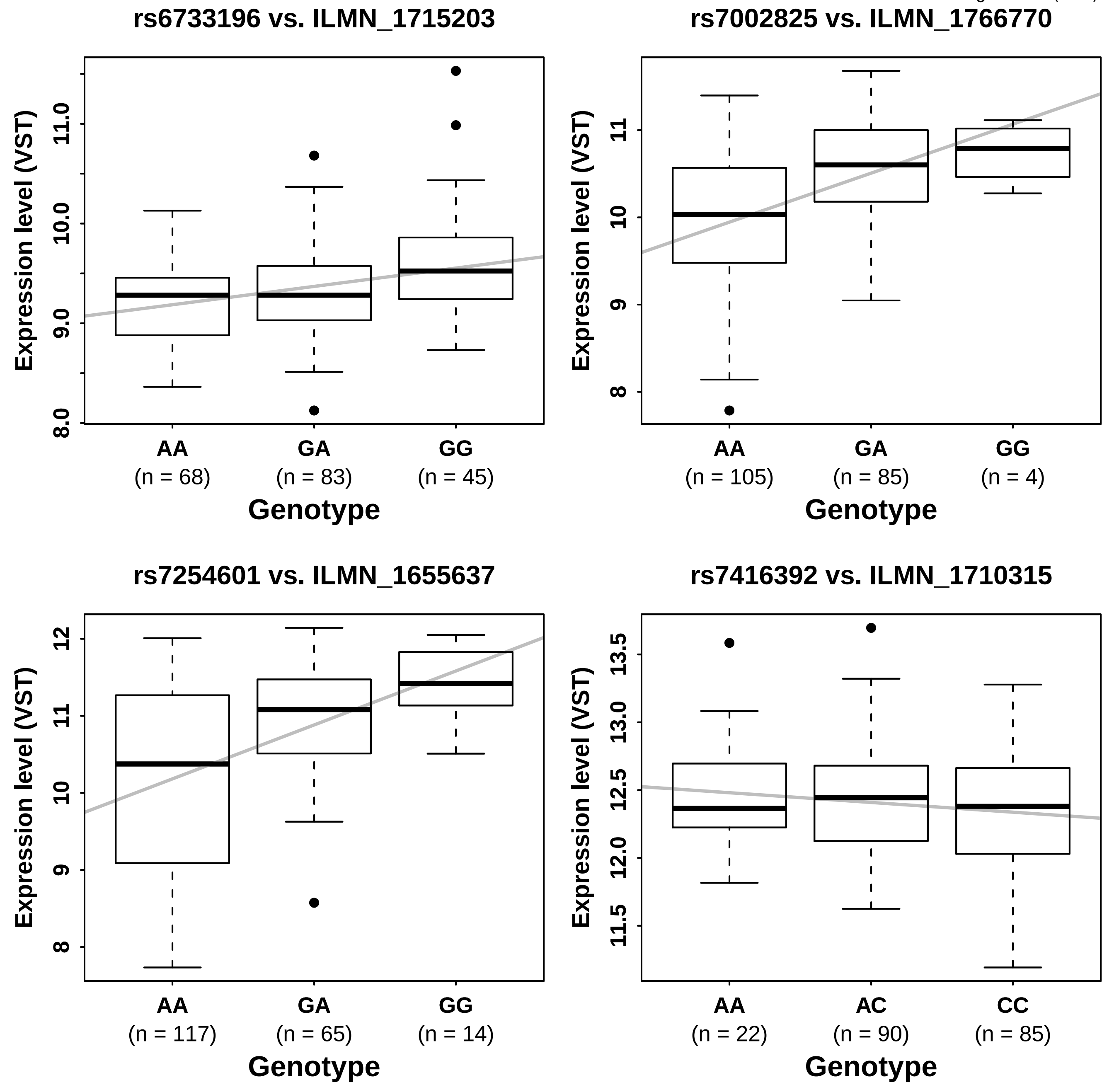

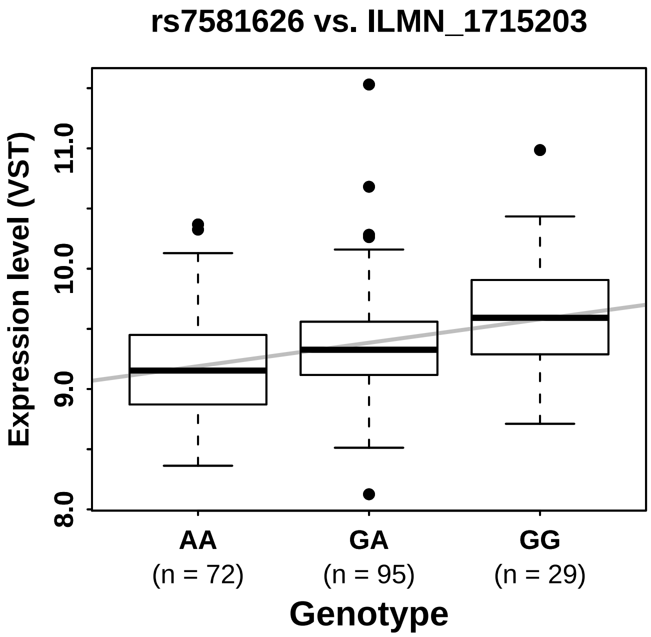
rs7581626 vs. ILMN_1715203
Expression level (VST)
8.0
9.0
10.0
11.0
AA
(n = 72)
GA
(n = 95)
GG
(n = 29)
Genotype

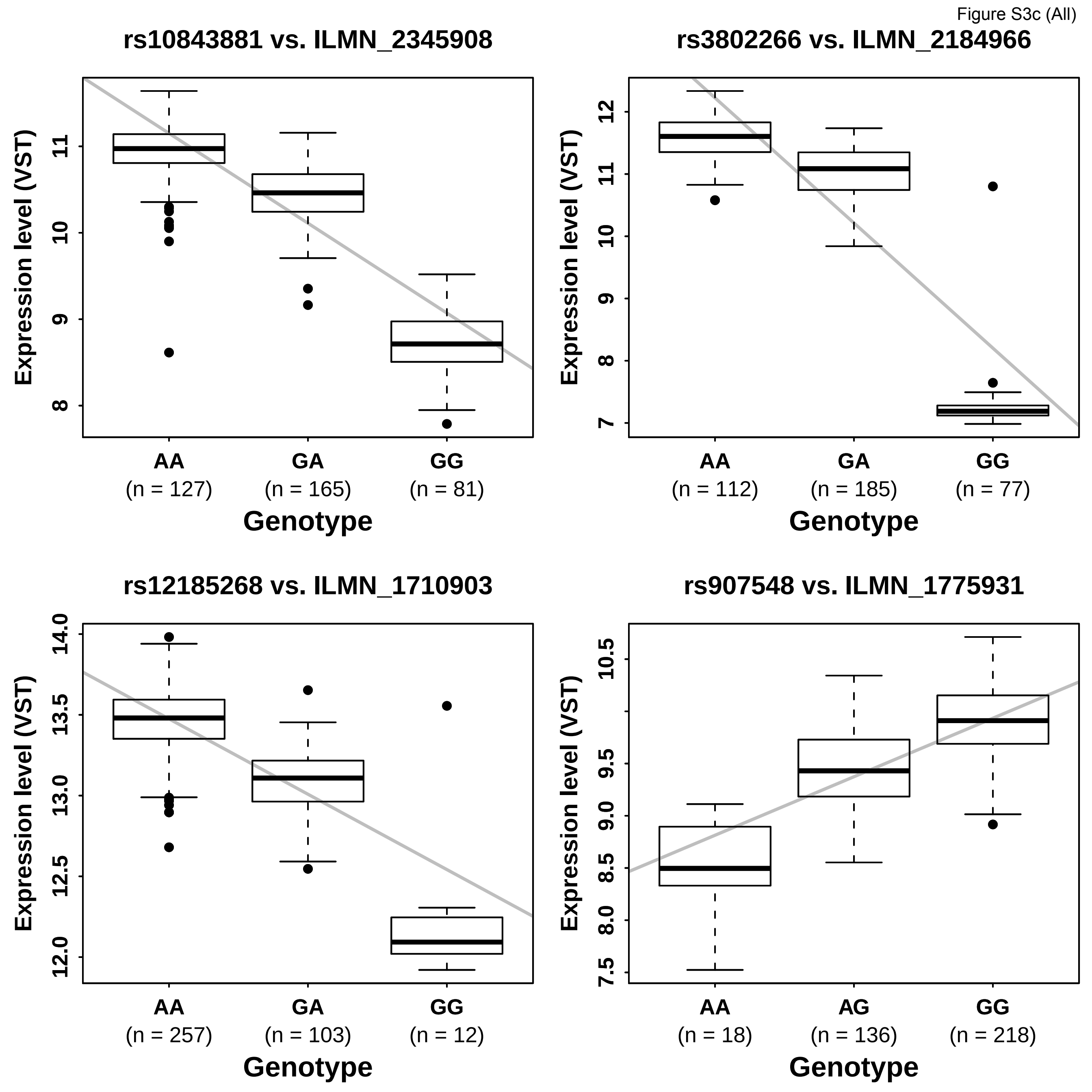
Figure S3c (All)
rs10843881 vs. ILMN_2345908
Expression level (VST)
11
10
9
8
AA
(n = 127)
GA
(n = 165)
GG
(n = 81)
Genotype
rs3802266 vs. ILMN_2184966
Expression level (VST)
12
11
10
9
8
7
AA
(n = 112)
GA
(n = 185)
GG
(n = 77)
Genotype
rs12185268 vs. ILMN_1710903
Expression level (VST)
14.0
13.5
13.0
12.5
12.0
AA
(n = 257)
GA
(n = 103)
GG
(n = 12)
Genotype
rs907548 vs. ILMN_1775931
Expression level (VST)
10.5
9.5
9.0
8.5
8.0
7.5
AA
(n = 18)
AG
(n = 136)
GG
(n = 218)
Genotype

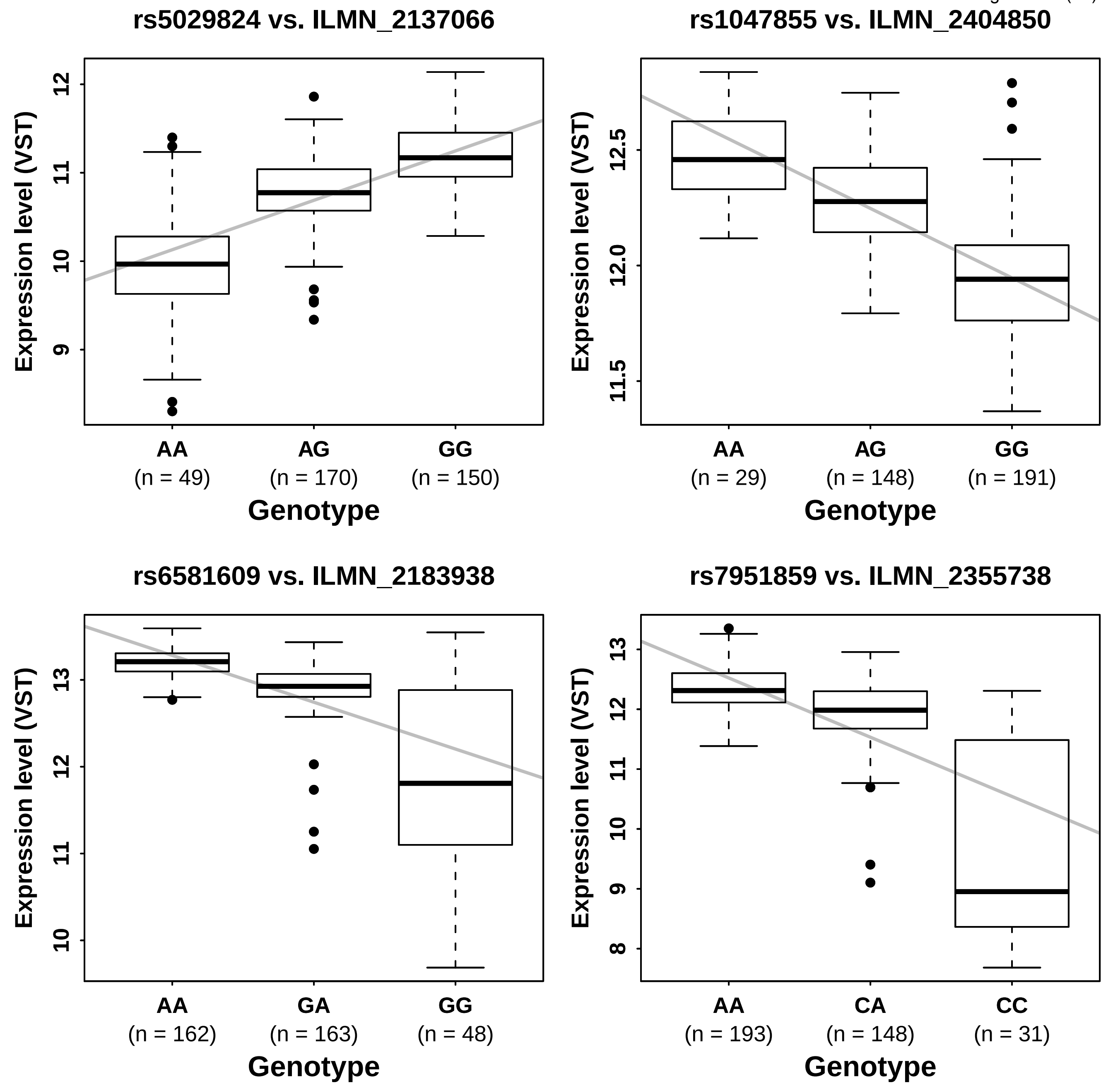
rs5029824 vs. ILMN_2137066
Expression level (VST)
9
10
11
12
AA
(n = 49)
AG
(n = 170)
GG
(n = 150)
Genotype
rs1047855 vs. ILMN_2404850
Expression level (VST)
11.5
12.0
12.5
AA
(n = 29)
AG
(n = 148)
GG
(n = 191)
Genotype
rs6581609 vs. ILMN_2183938
Expression level (VST)
10
11
12
13
AA
(n = 162)
GA
(n = 163)
GG
(n = 48)
Genotype
rs7951859 vs. ILMN_2355738
Expression level (VST)
8
9
10
11
12
13
AA
(n = 193)
CA
(n = 148)
CC
(n = 31)
Genotype

Figure S3c (All)

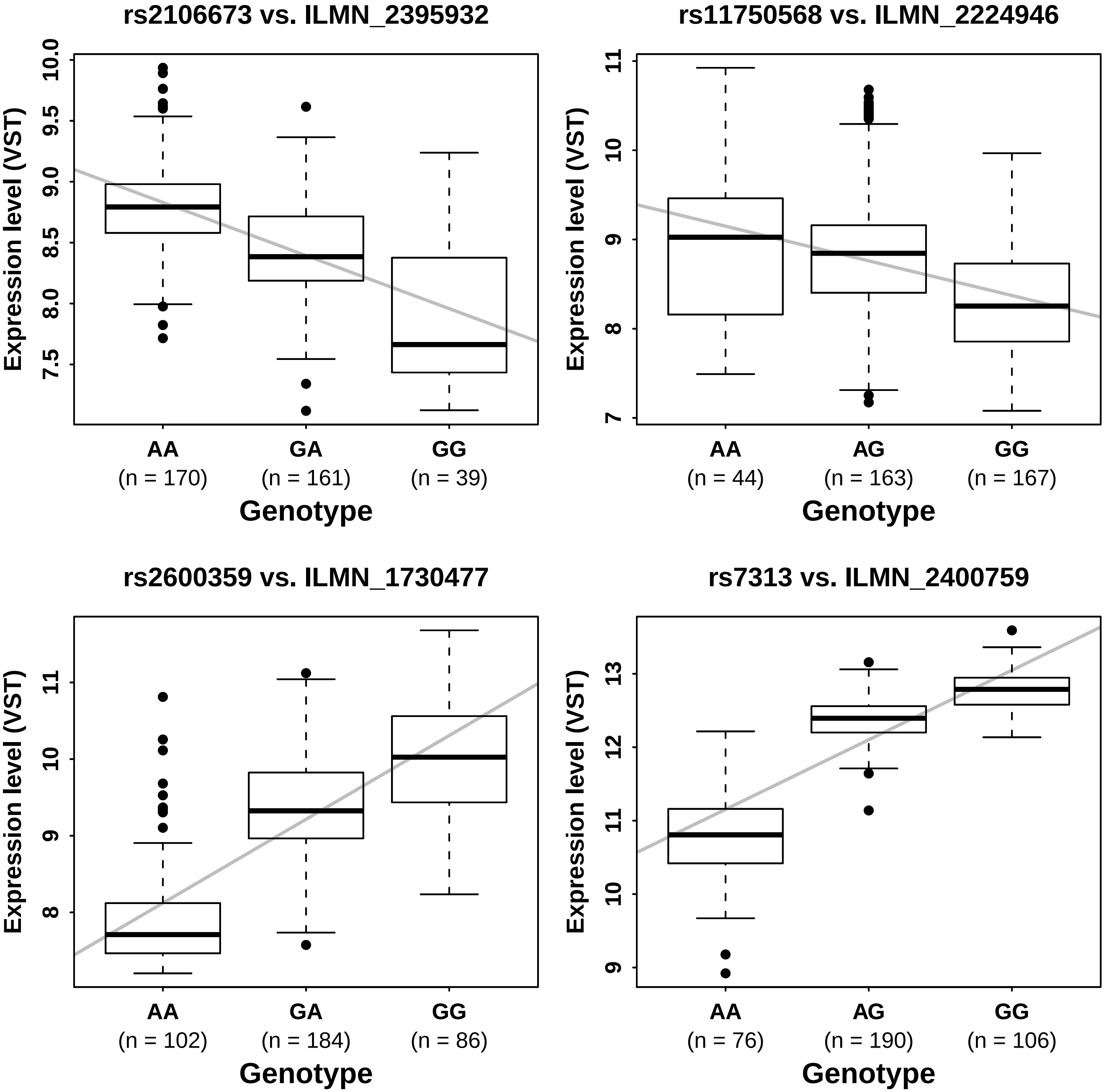

Figure S3c (All)

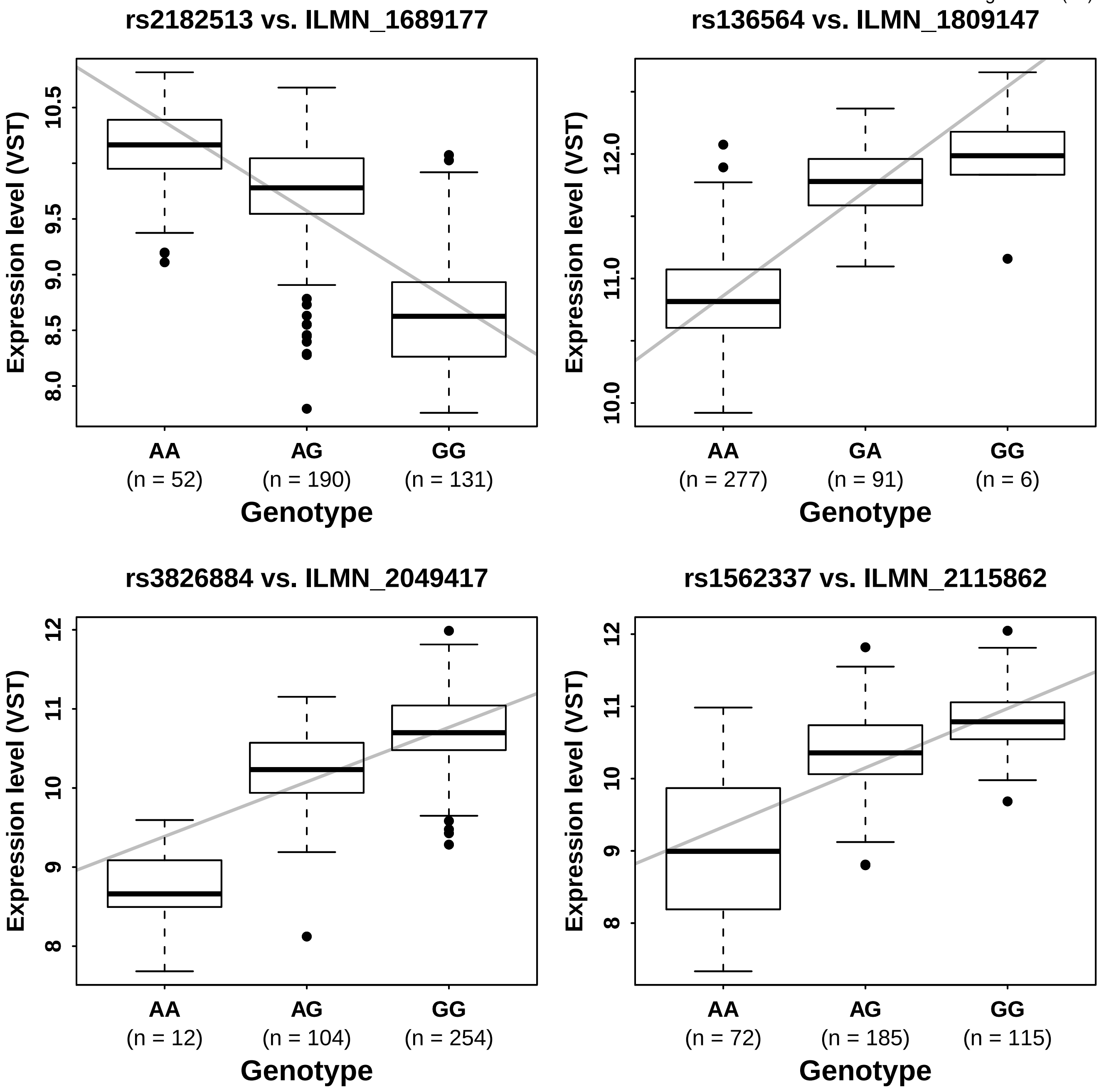

Figure S3c (All)

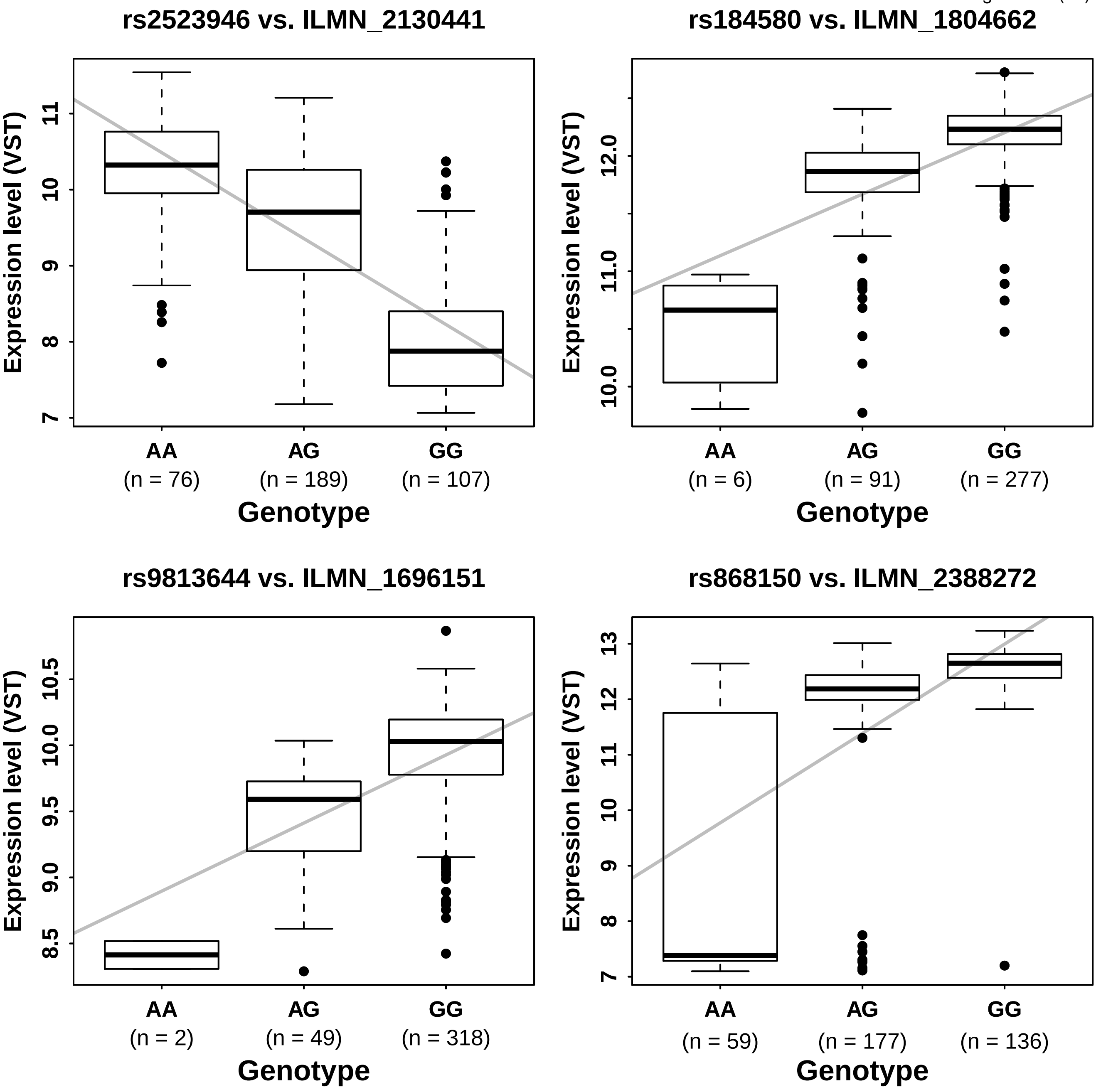

Figure S3c (All)

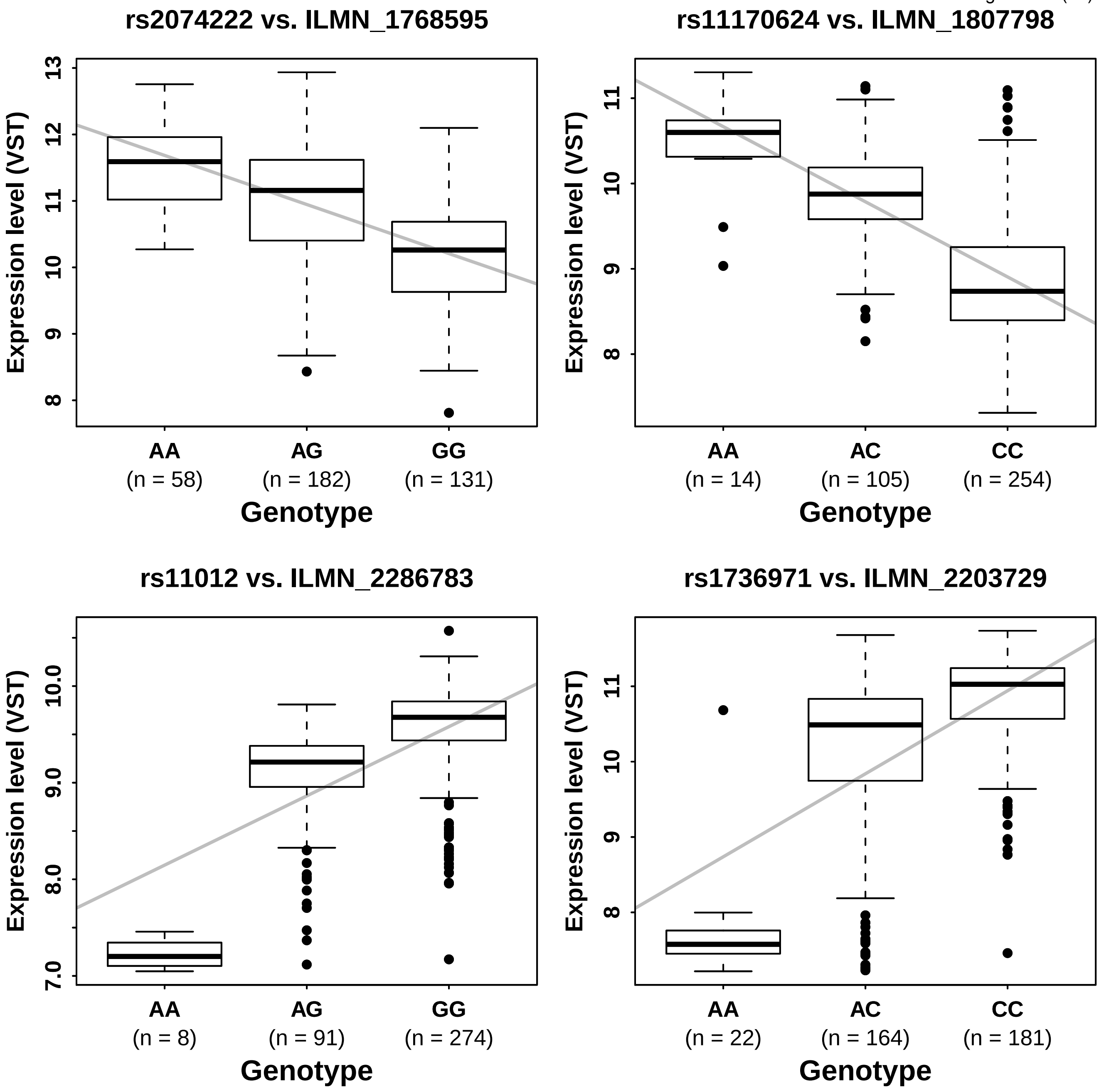

Figure S3c (All)

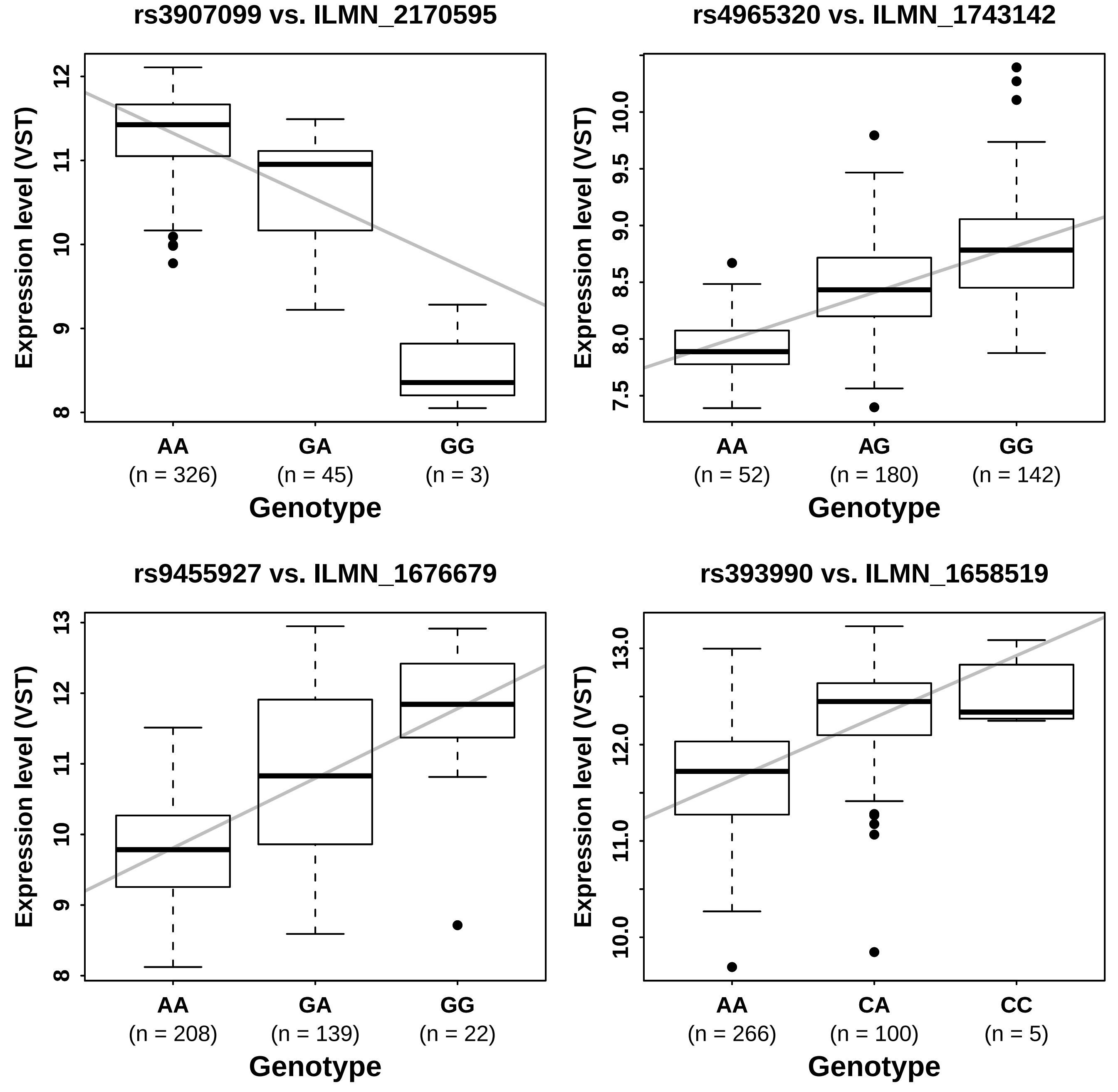

Figure S3c (All)

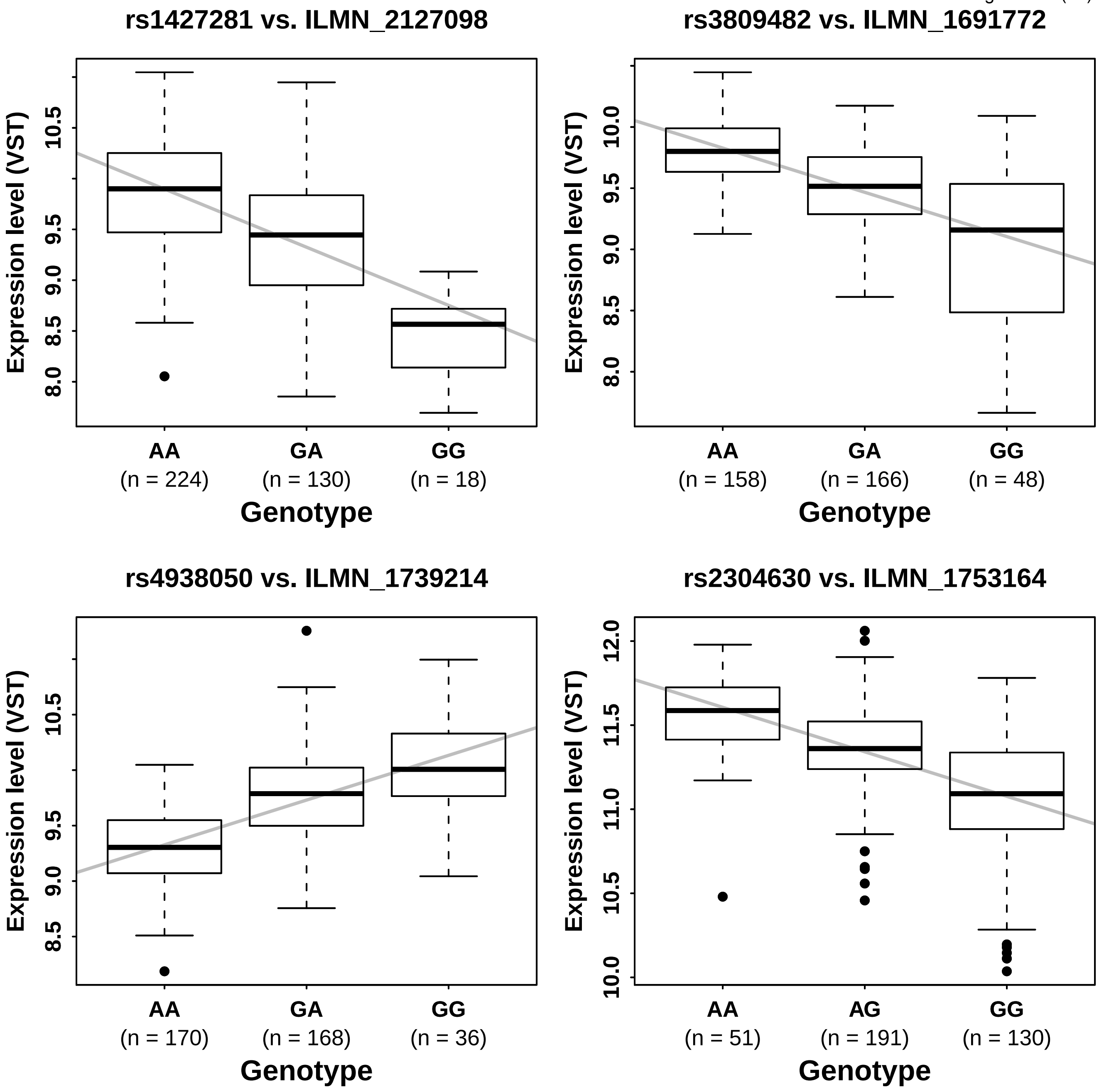

Figure S3c (All)

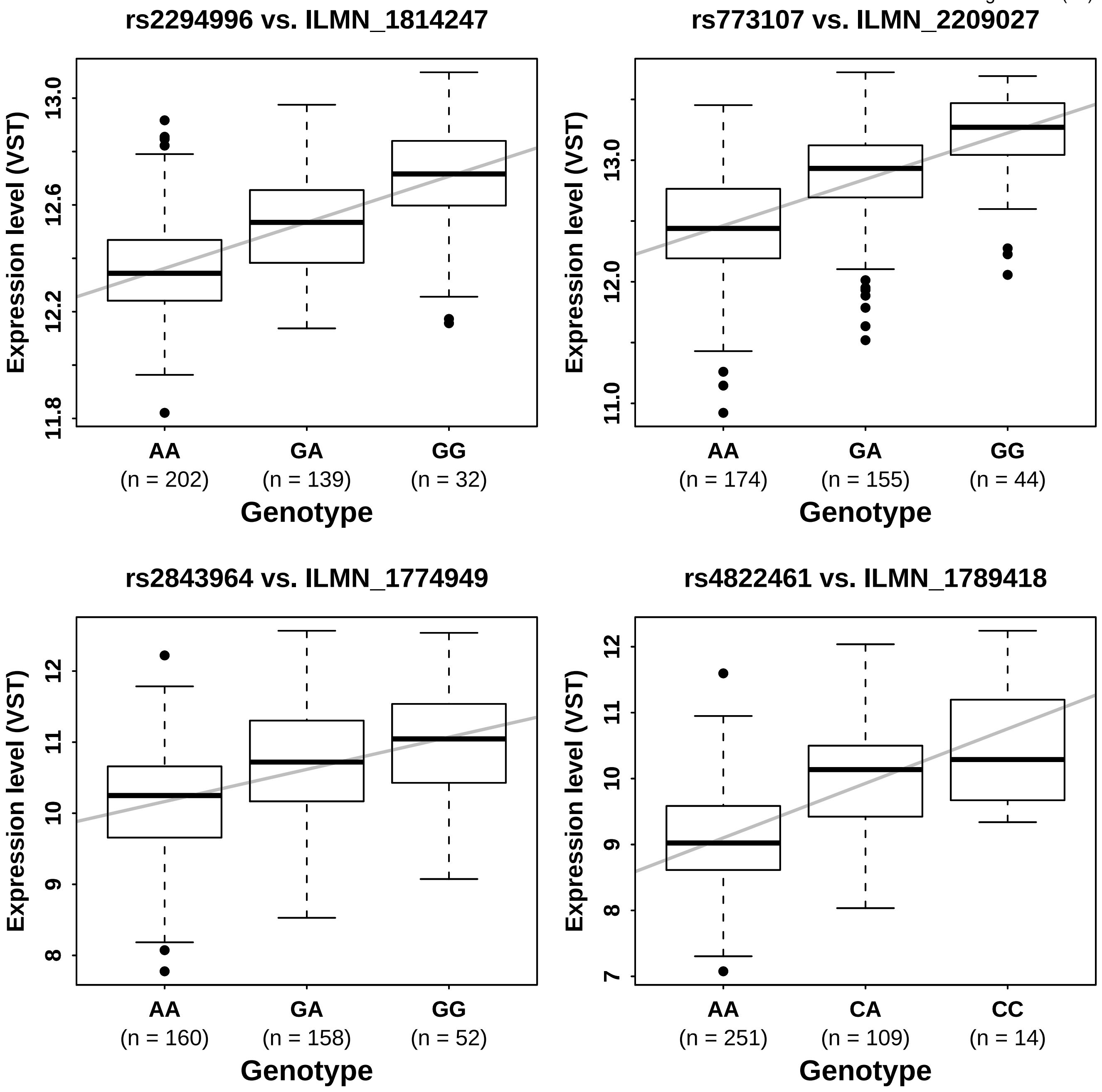

Figure S3c (All)

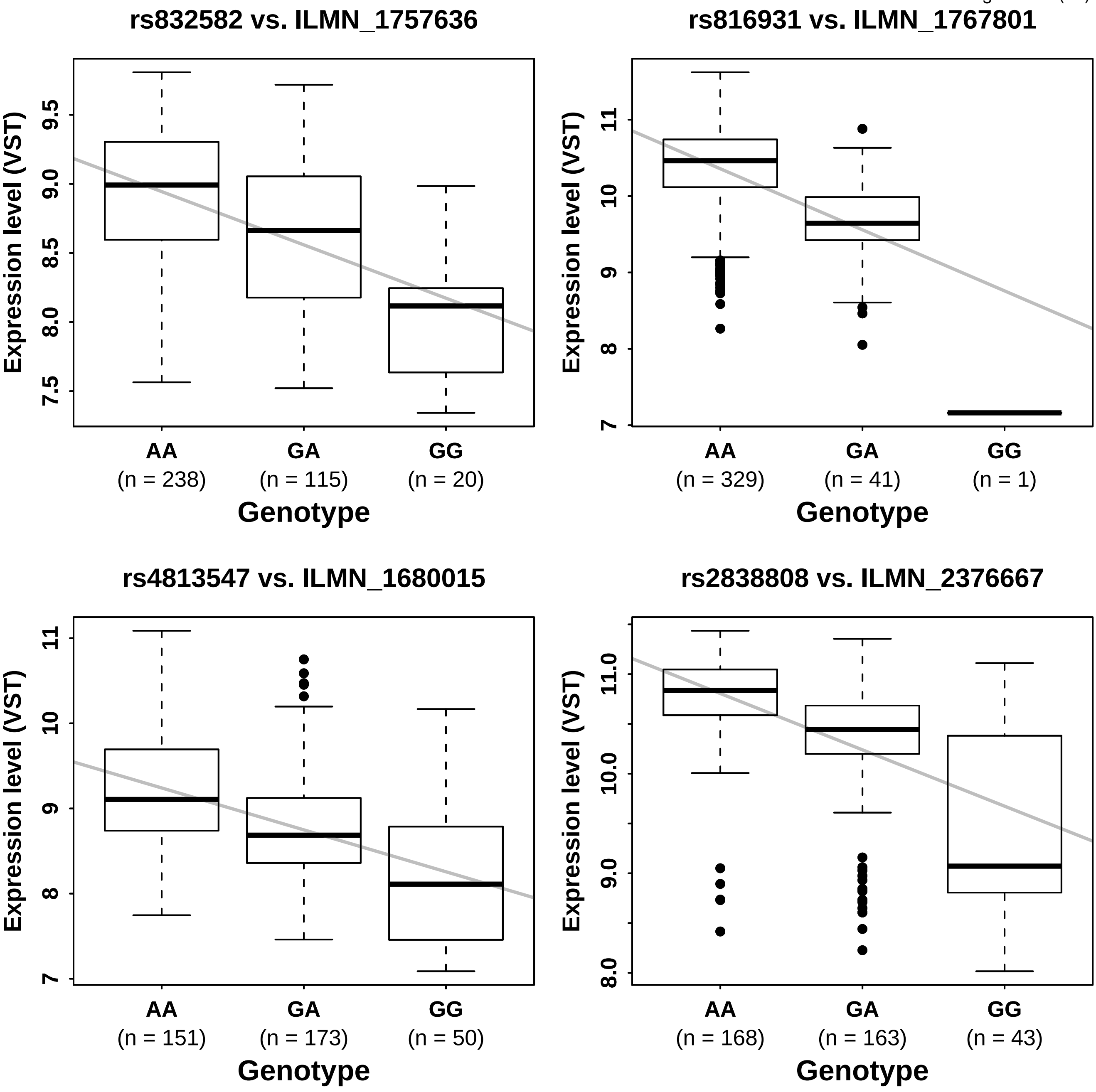

Figure S3c (All)

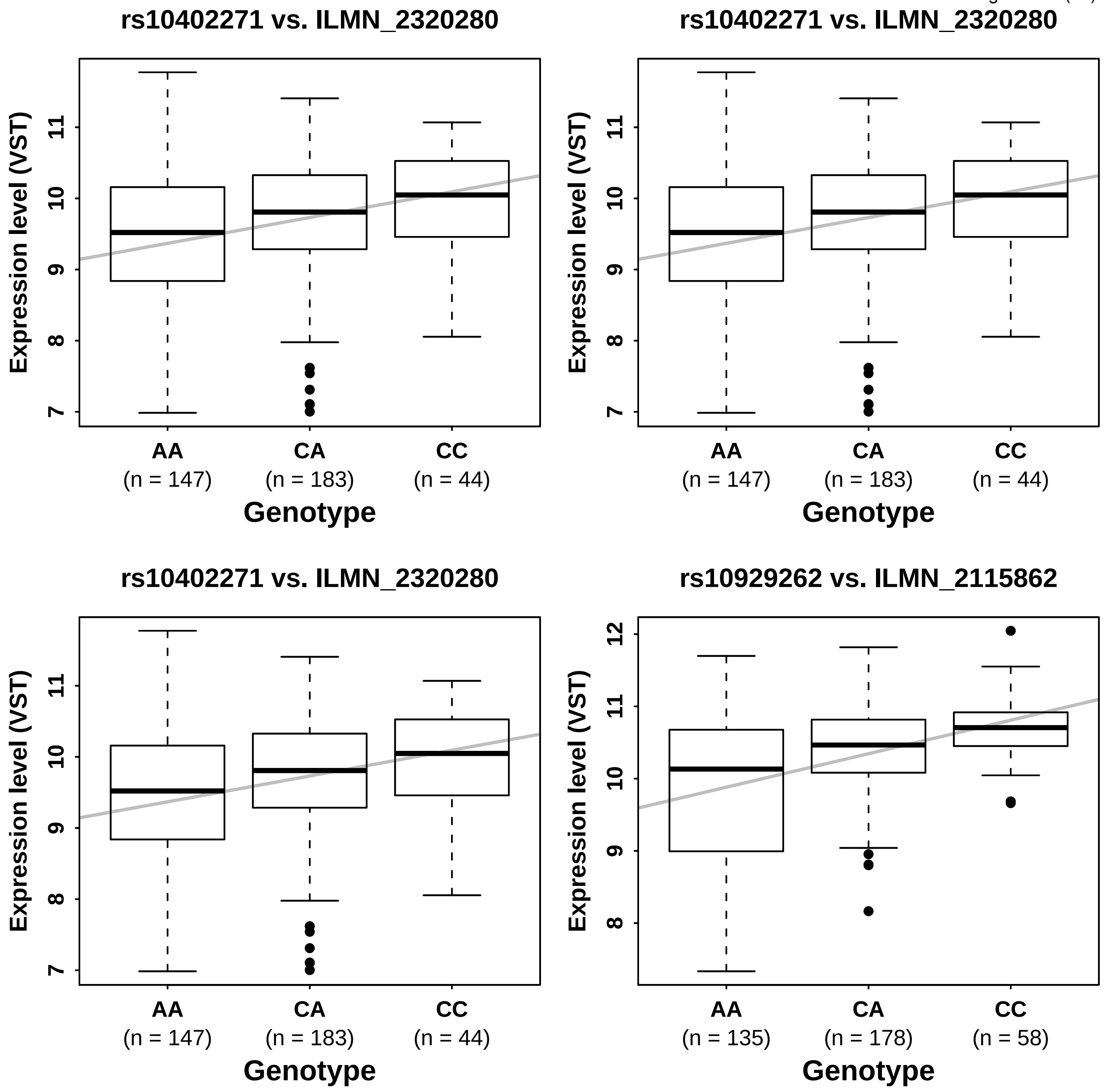

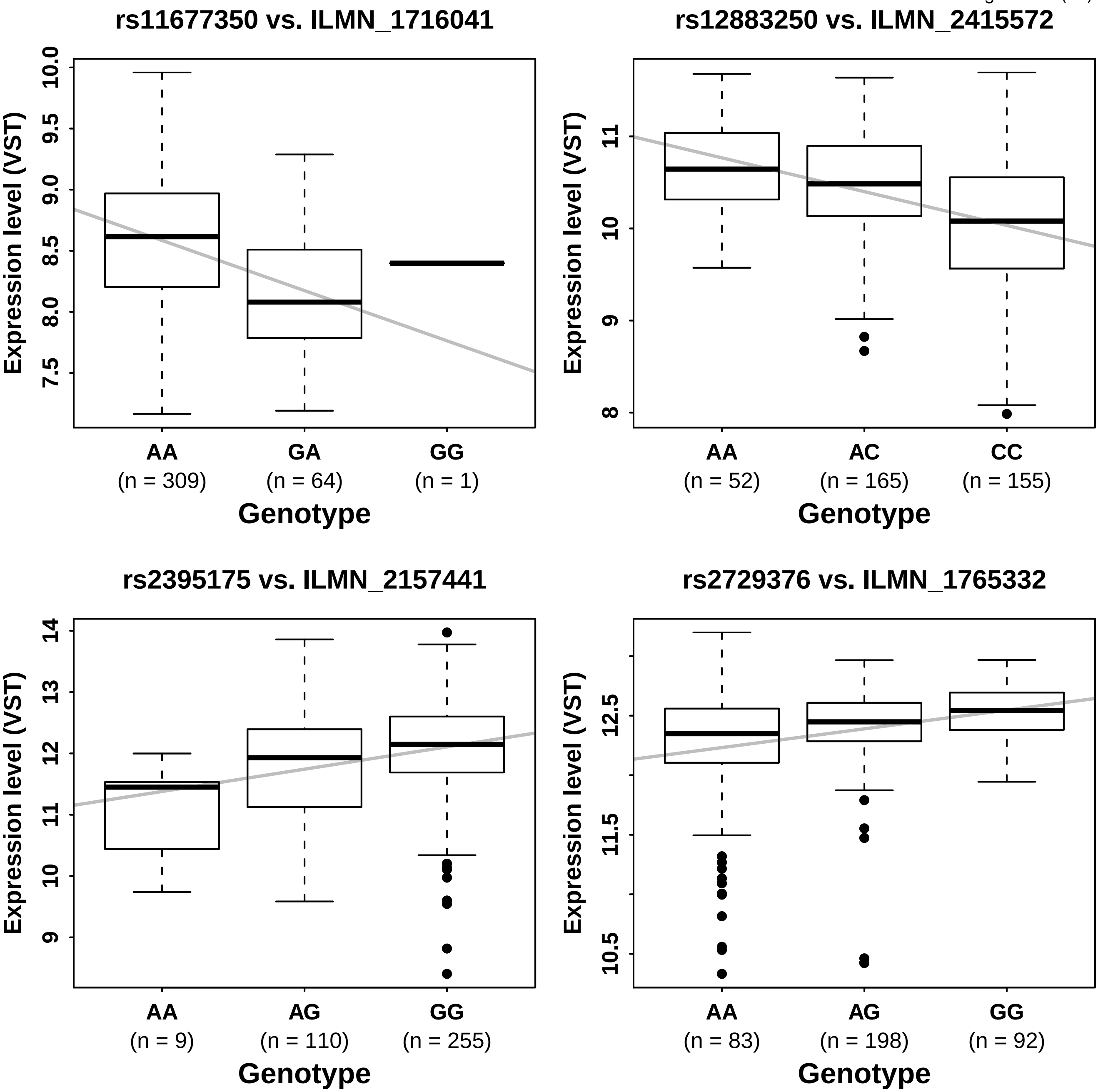
rs11677350 vs. ILMN_1716041
Expression level (VST)
10.0
9.5
9.0
8.5
8.0
7.5
AA
(n = 309)
GA
(n = 64)
GG
(n = 1)
Genotype
rs12883250 vs. ILMN_2415572
Expression level (VST)
11
10
9
8
AA
(n = 52)
AC
(n = 165)
CC
(n = 155)
Genotype
rs2395175 vs. ILMN_2157441
Expression level (VST)
14
13
12
11
10
9
AA
(n = 9)
AG
(n = 110)
GG
(n = 255)
Genotype
rs2729376 vs. ILMN_1765332
Expression level (VST)
12.5
11.5
10.5
AA
(n = 83)
AG
(n = 198)
GG
(n = 92)
Genotype

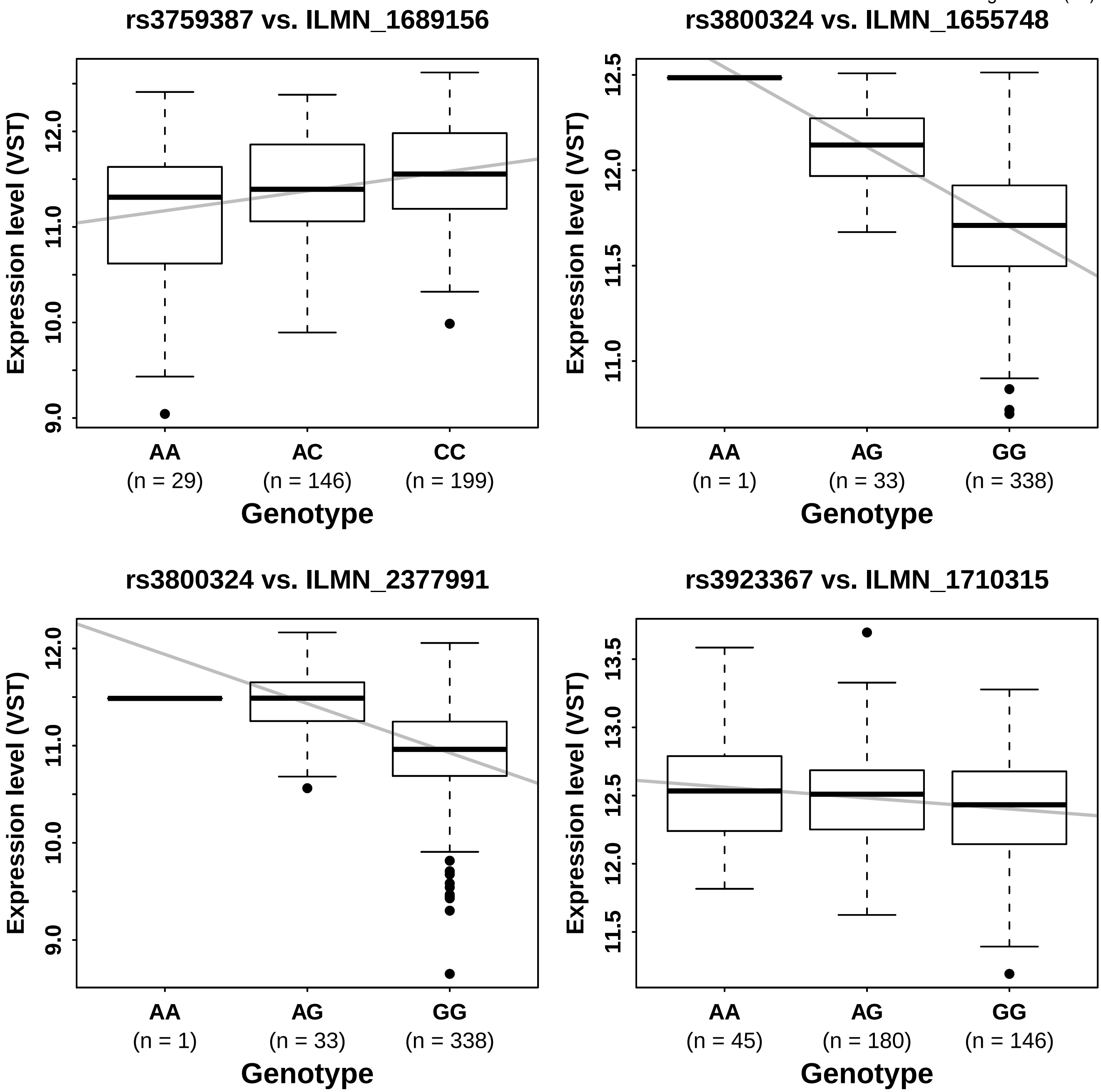
rs3759387 vs. ILMN_1689156
Expression level (VST)
9.0
10.0
11.0
12.0
AA
(n = 29)
AC
(n = 146)
CC
(n = 199)
Genotype
rs3800324 vs. ILMN_1655748
Expression level (VST)
11.0
11.5
12.0
12.5
AA
(n = 1)
AG
(n = 33)
GG
(n = 338)
Genotype
rs3800324 vs. ILMN_2377991
Expression level (VST)
9.0
10.0
11.0
12.0
AA
(n = 1)
AG
(n = 33)
GG
(n = 338)
Genotype
rs3923367 vs. ILMN_1710315
Expression level (VST)
11.5
12.0
12.5
13.0
13.5
AA
(n = 45)
AG
(n = 180)
GG
(n = 146)
Genotype

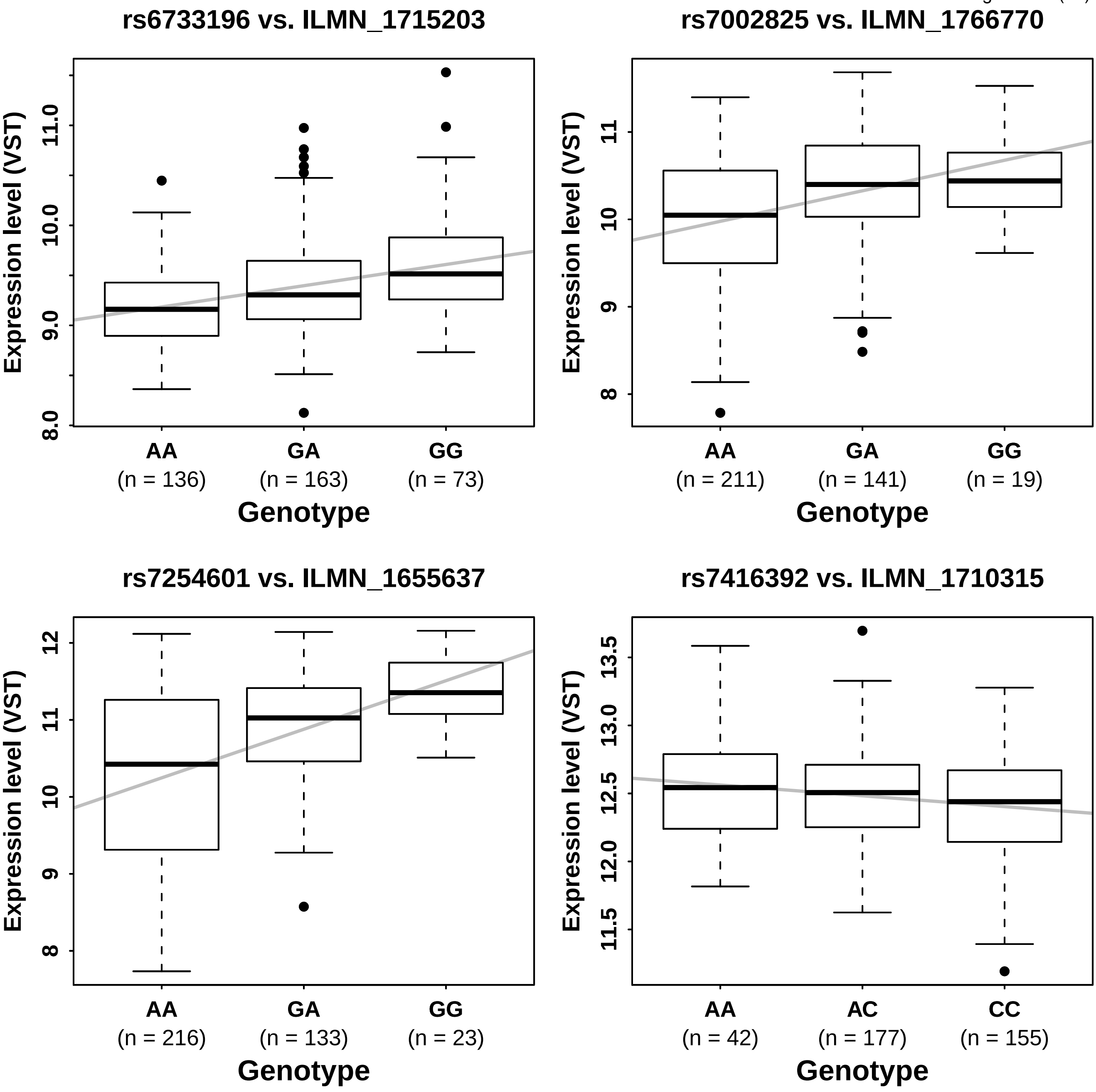
rs6733196 vs. ILMN_1715203
Expression level (VST)
8.0
9.0
10.0
11.0
AA
(n = 136)
GA
(n = 163)
GG
(n = 73)
Genotype
rs7002825 vs. ILMN_1766770
Expression level (VST)
8
9
10
11
AA
(n = 211)
GA
(n = 141)
GG
(n = 19)
Genotype
rs7254601 vs. ILMN_1655637
Expression level (VST)
8
9
10
11
12
AA
(n = 216)
GA
(n = 133)
GG
(n = 23)
Genotype
rs7416392 vs. ILMN_1710315
Expression level (VST)
11.5
12.0
12.5
13.0
13.5
AA
(n = 42)
AC
(n = 177)
CC
(n = 155)
Genotype

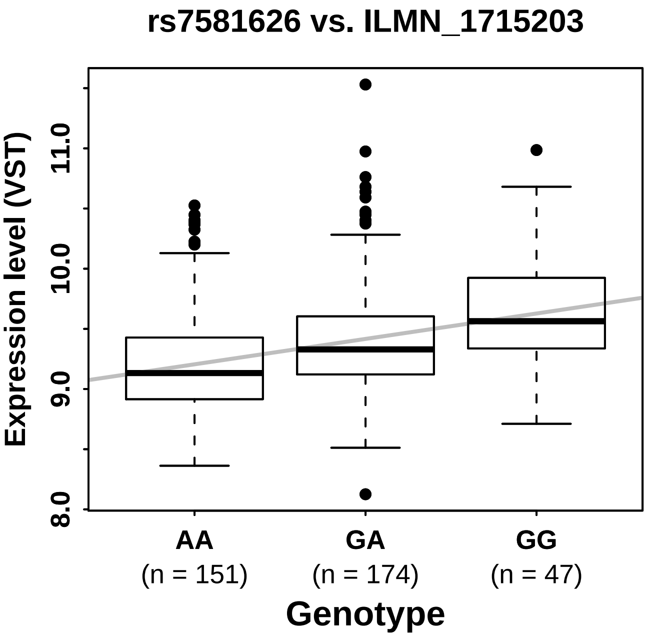
rs7581626 vs. ILMN_1715203
Expression level (VST)
8.0
9.0
10.0
11.0
AA
(n = 151)
GA
(n = 174)
GG
(n = 47)
Genotype
