# Supplementary figures and images for: Brain Expression Genome-Wide Association Study (eGWAS) Identifies Human Disease-Associated Variants
Source: PLoS Genet. 2012 Jun 7;8(6):e1002707. doi: 10.1371/journal.pgen.1002707 (PMC3369937; doi:10.1371/journal.pgen.1002707)

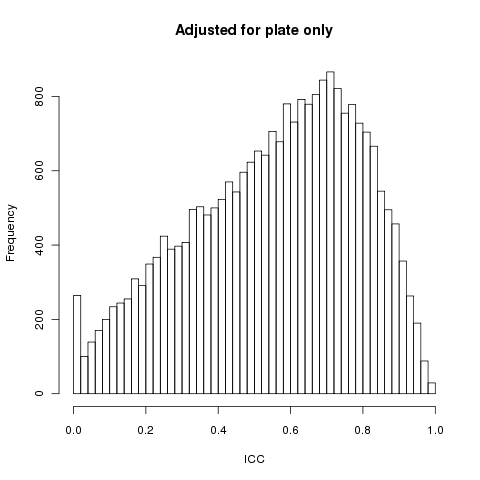

Supplement: Figure S4 — Histogram of intra-class coefficients (ICC) for the cerebellar probe expressions. Using 15 replicate samples, ICC, which is the between-subject variance, as a percentage of the total variance in probe expression, was estimated for 17,121 probes. (JPG) [file pgen.1002707.s005.jpg]

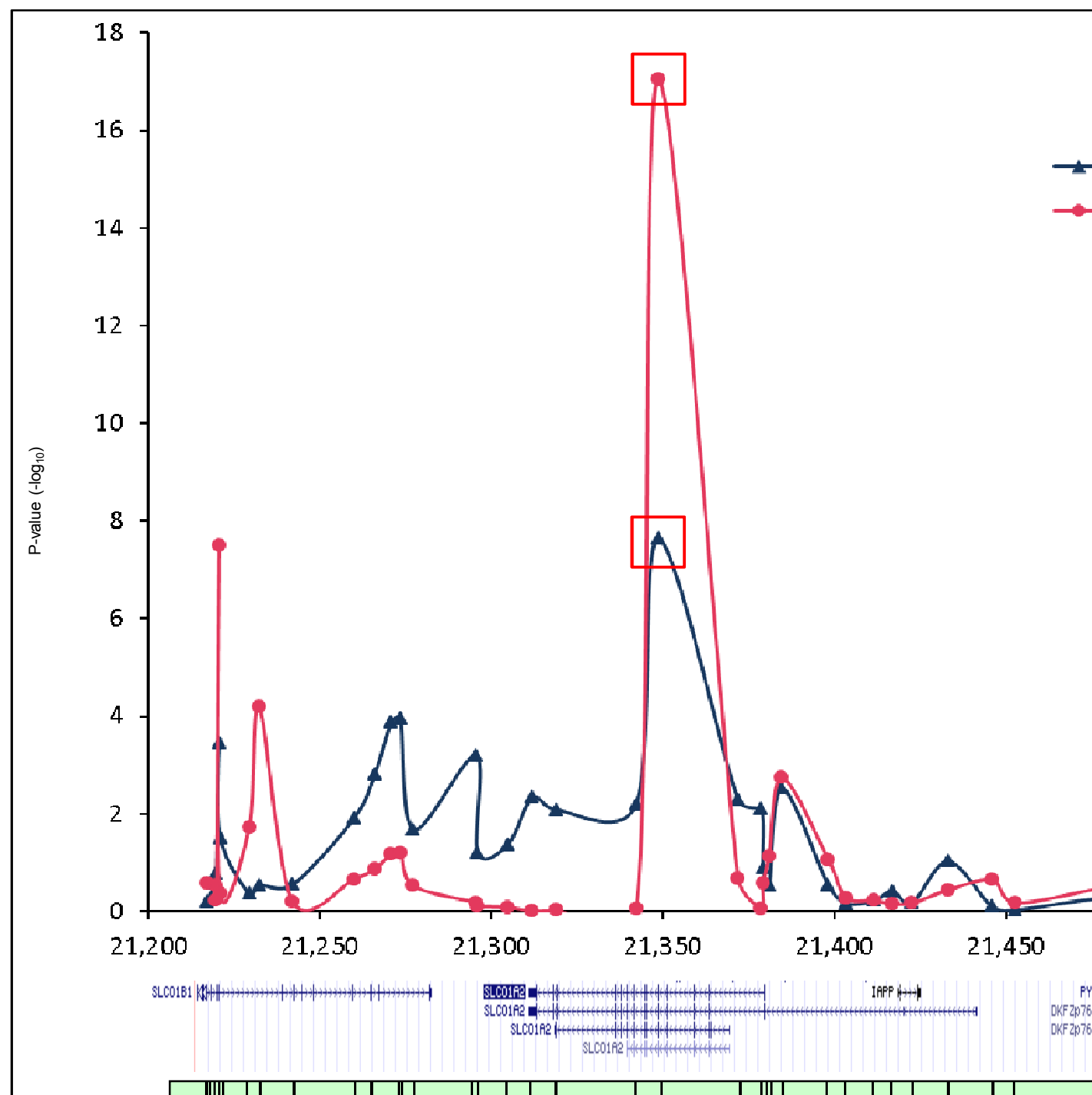

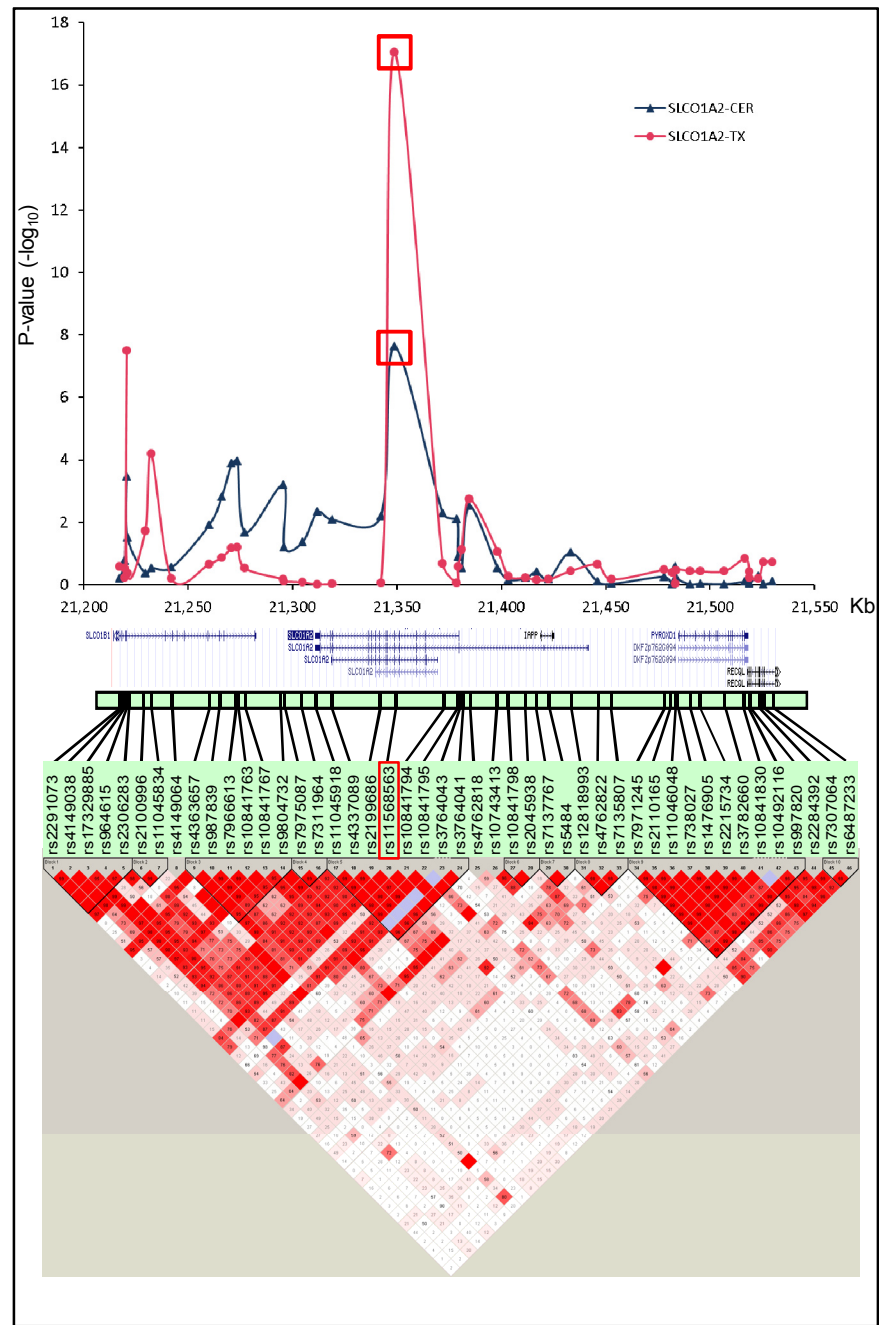

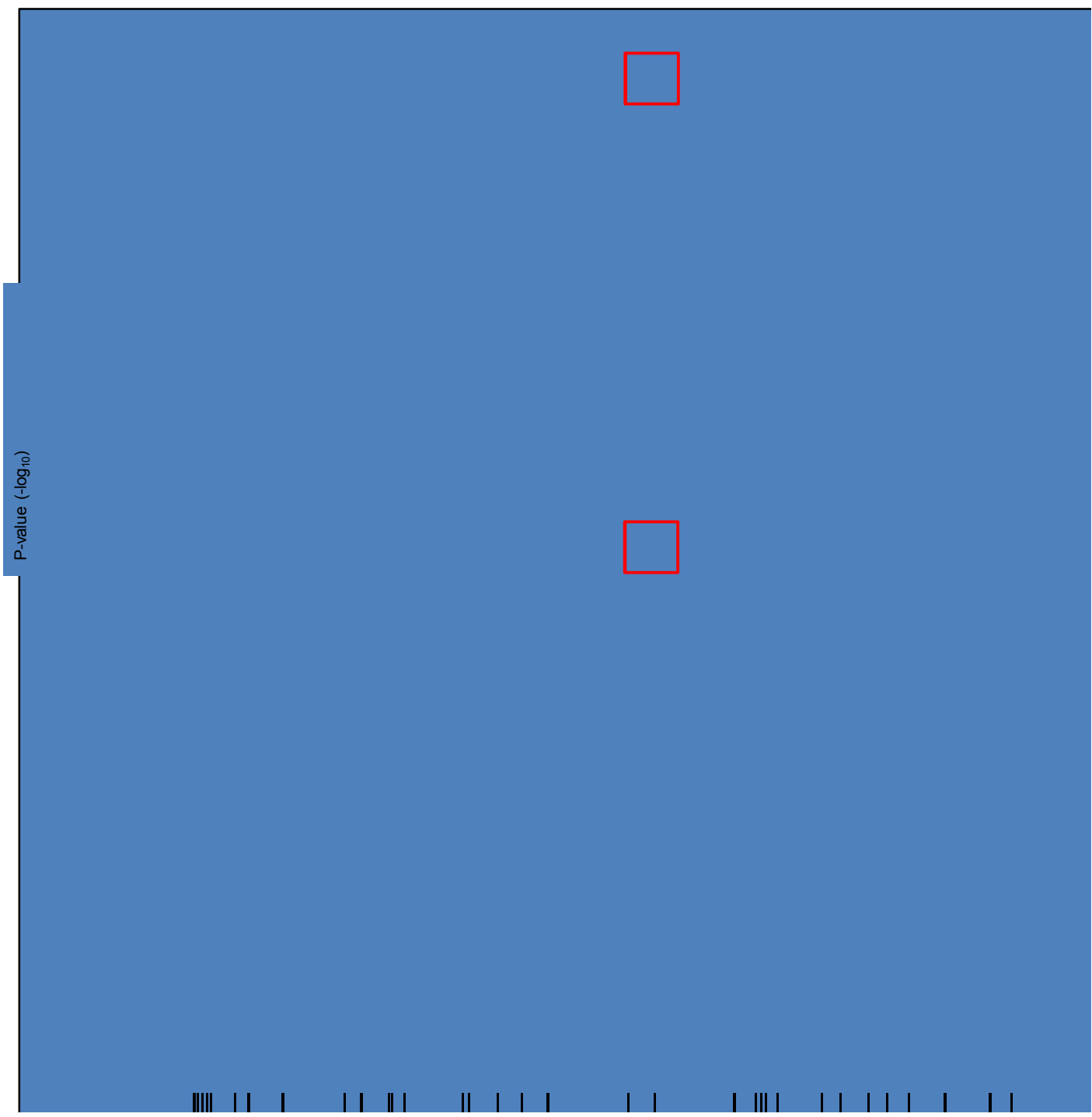

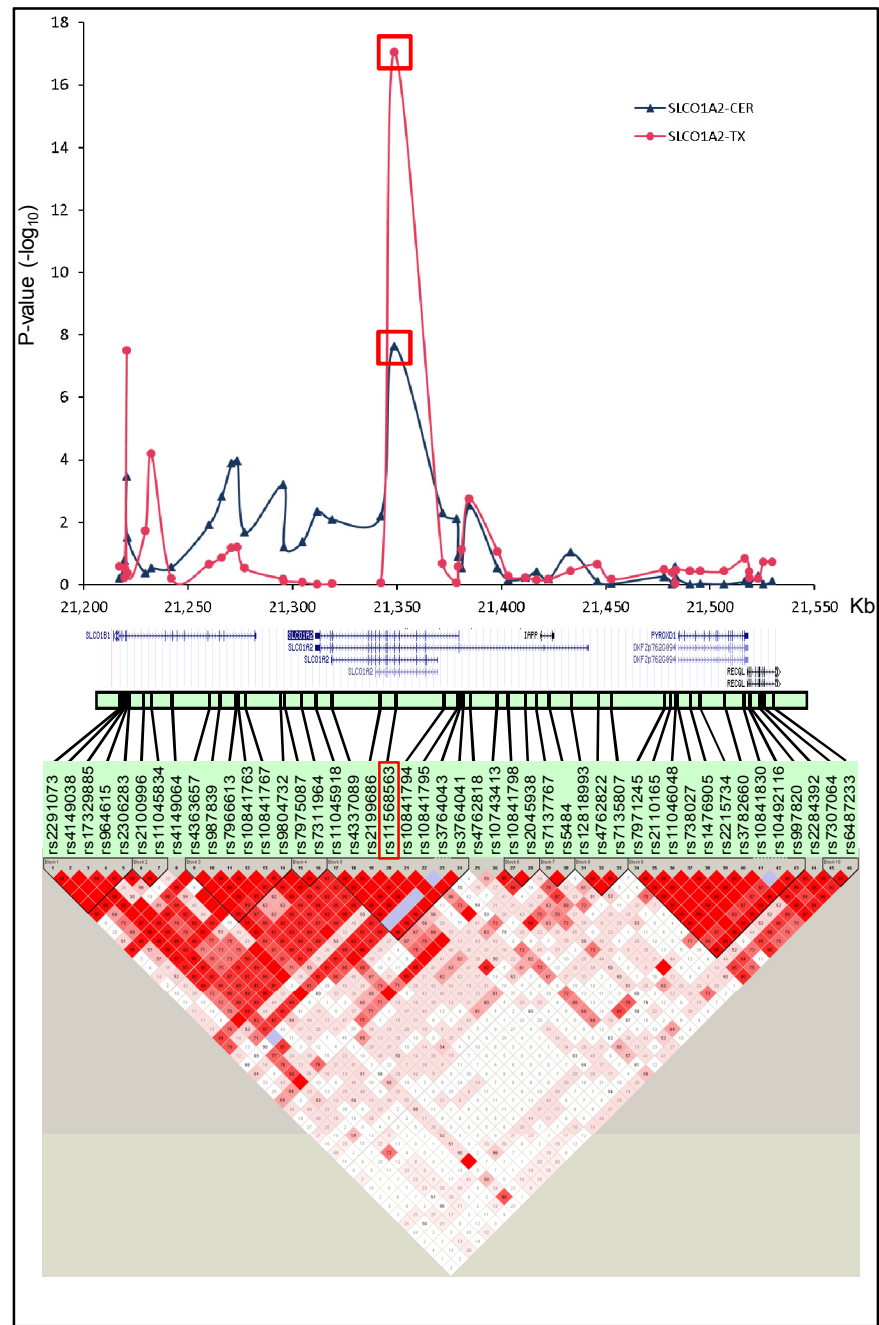

Supplement: Figure S5 — Data plots of SNPs tested for association with expression levels of SLCO1A2 in the Temporal Cortex and Cerebellum. Forty-six SNPs were tested for association of SLCO1A2 levels in the Cerebellum (Blue lines) and Temporal Cortex (Pink lines). P-values were transformed using −log10 and are plotted against the position of each SNP along the chromosome (Kbp). Genes found within the locus boundaries are shown from the UCSC genome browser (http://genome.ucsc.edu/). The LD across the locus is represented by a plot generated with Haploview, using data from the Mayo GWAS. The top eSNP in this study, rs11568563, is highlighted on the p-value plot by red squares and a red box around the SNP in the list of rs numbers. This is also the top PSP-associating SNP at this locus in Hoglinger et al. (Nat Genet, 2011) [27]. (PDF) [file pgen.1002707.s006.pdf]

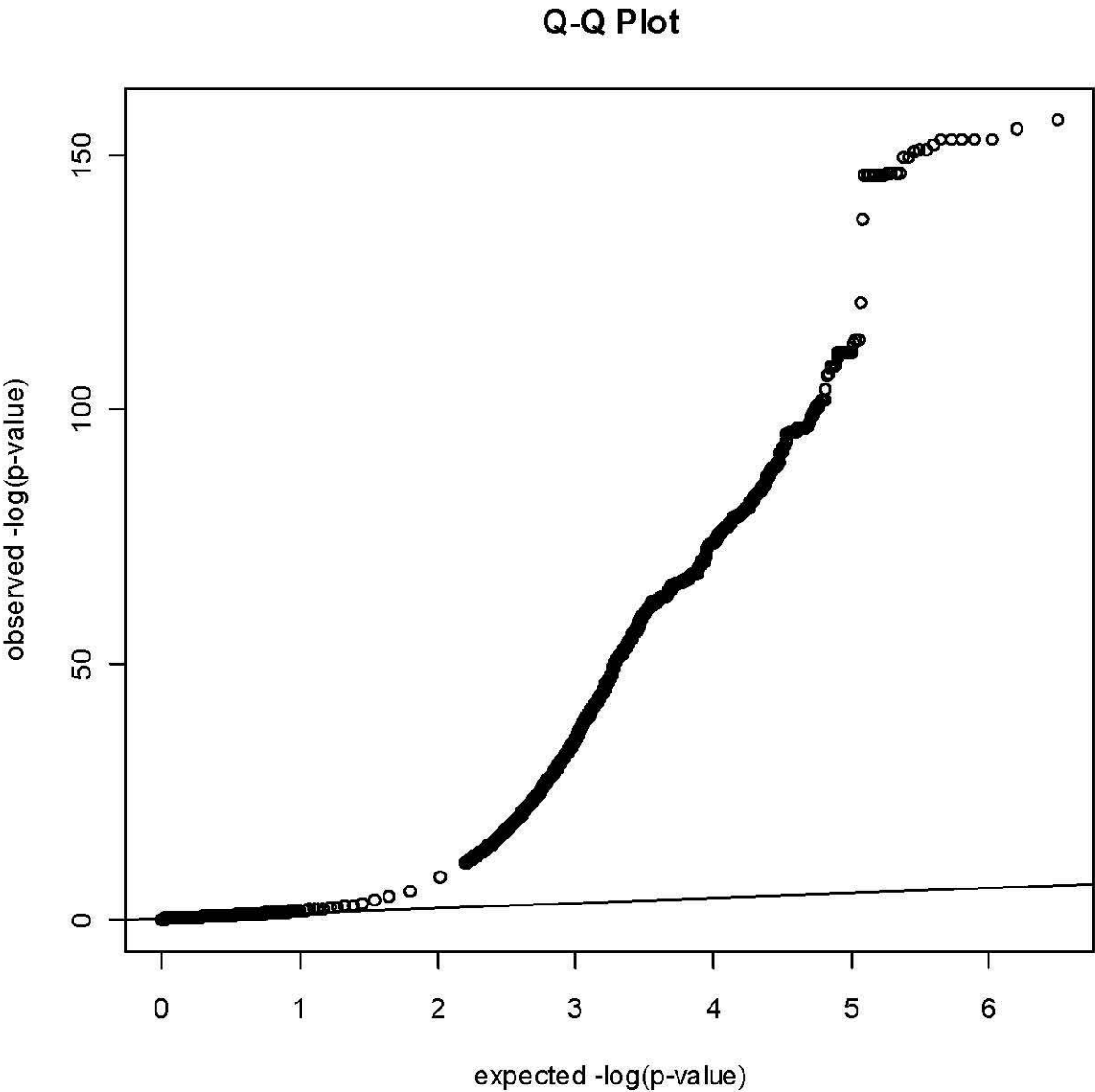

Figure S6b (CER, after genomic inflation adjustment)

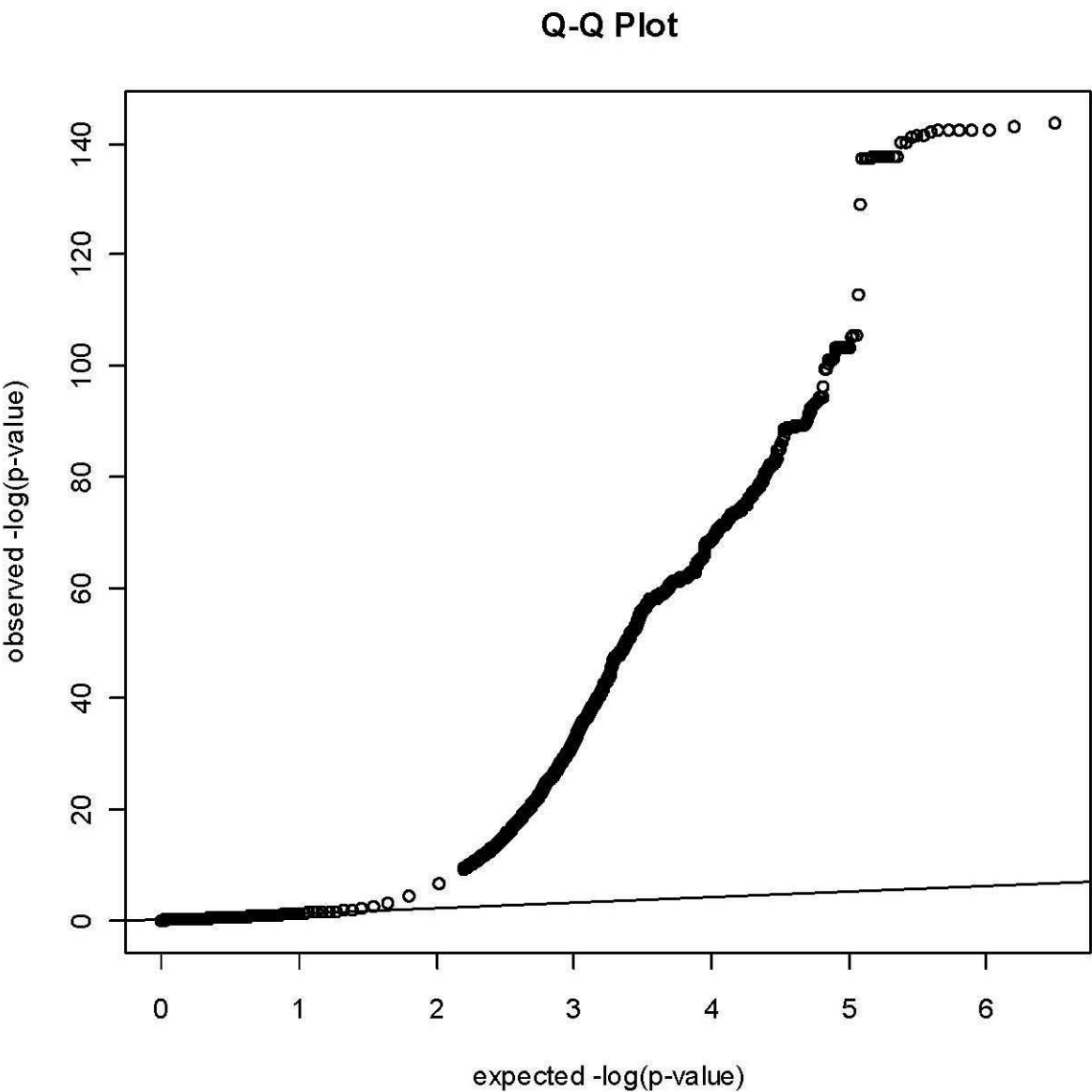

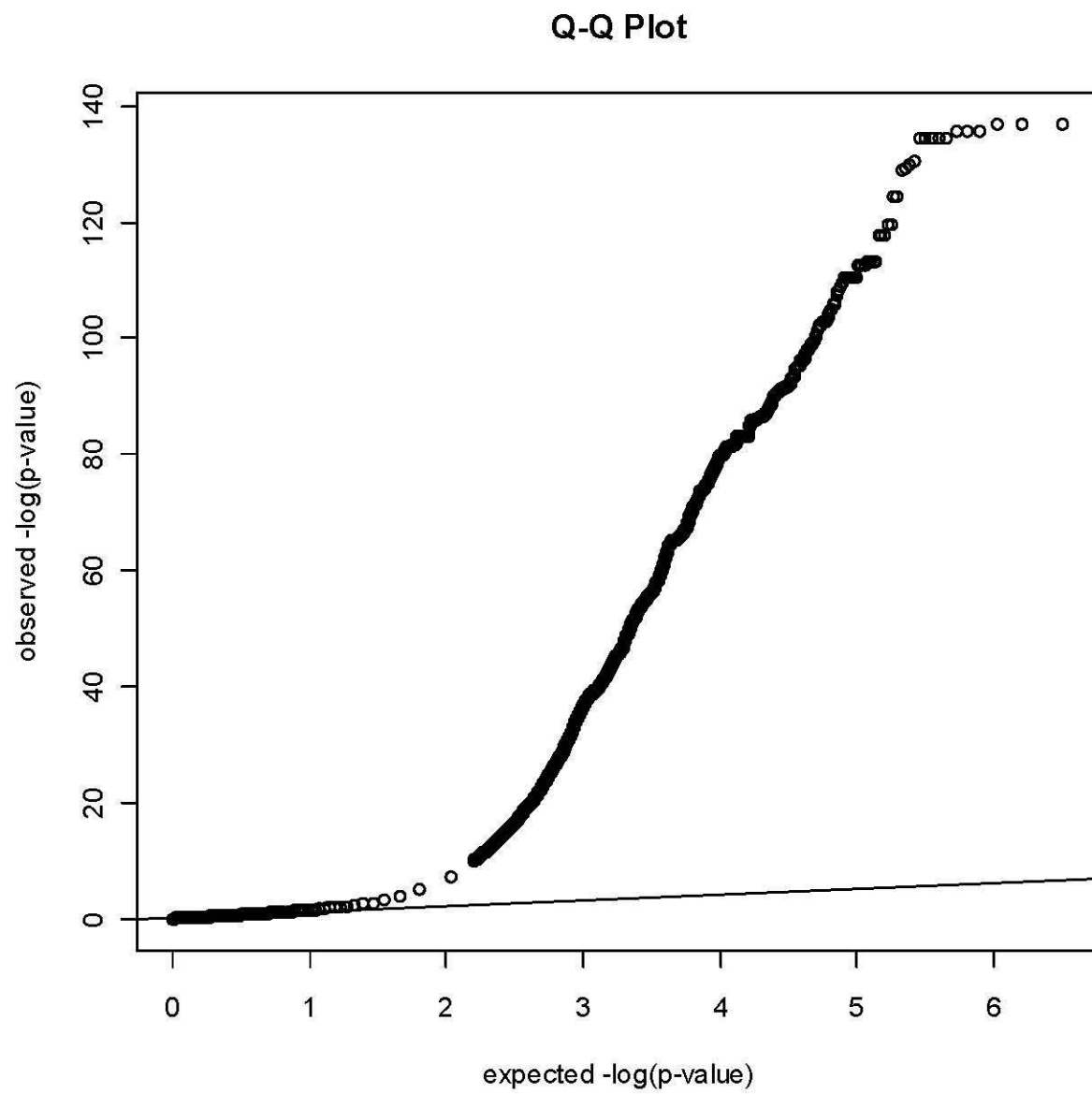

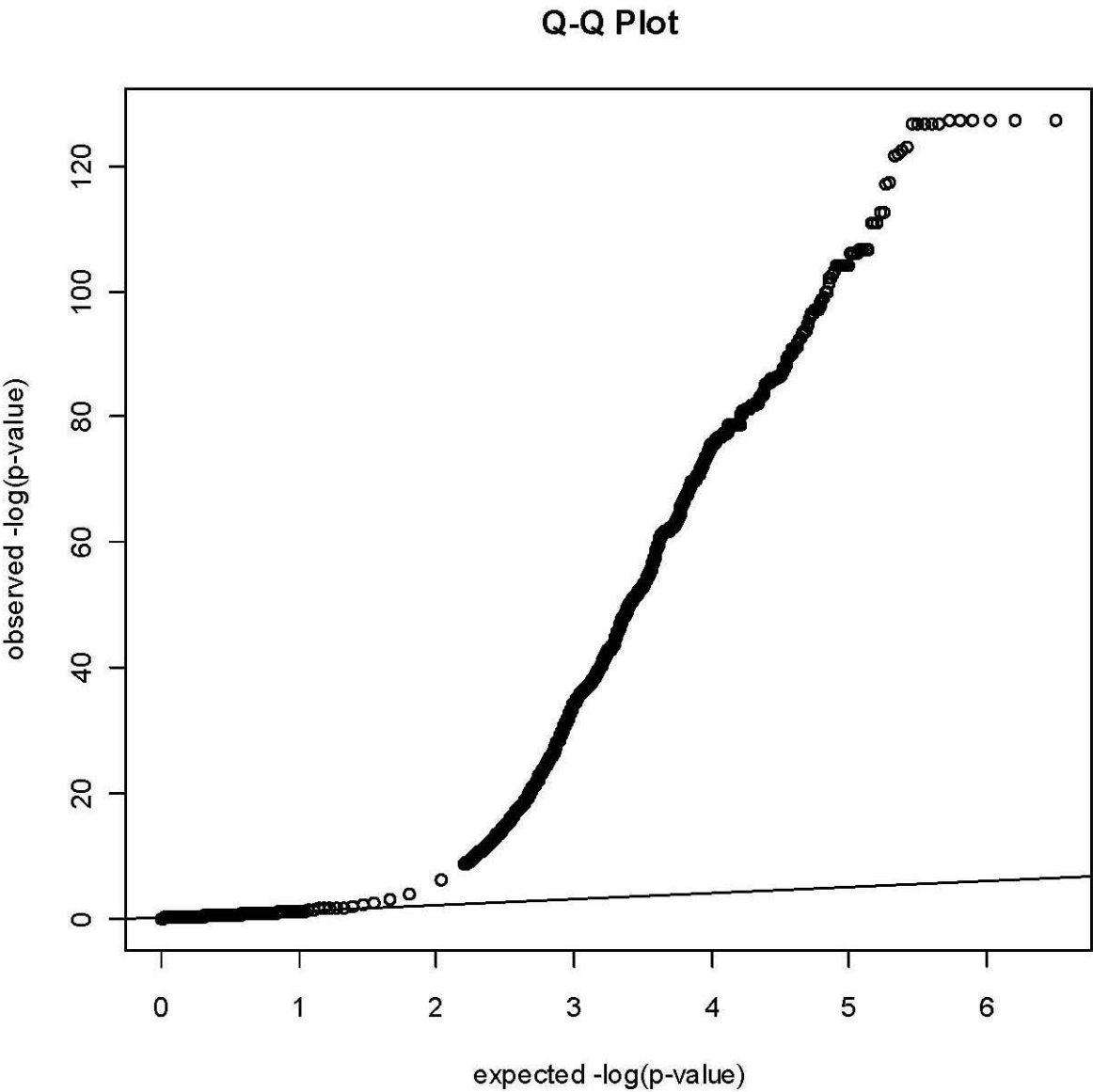

Supplement: Figure S6 — Q-Q-Plots for cerebellar and temporal cortex cisSNP/transcript associations with the HapMap phase 2 imputed genotypes: Q-Q plots of observed (y-axis) versus expected (X-axis) −log(p) values of association for all cisSNP/transcript associations in the combined dataset obtained before (a) and after (b) genomic inflation-adjustments, as discussed in the text. Also shown are the Q-Q plots for the temporal cortex associations in the combined dataset obtained obtained before (c) and after (d) inflation-adjustments. (PDF) [file pgen.1002707.s007.pdf]

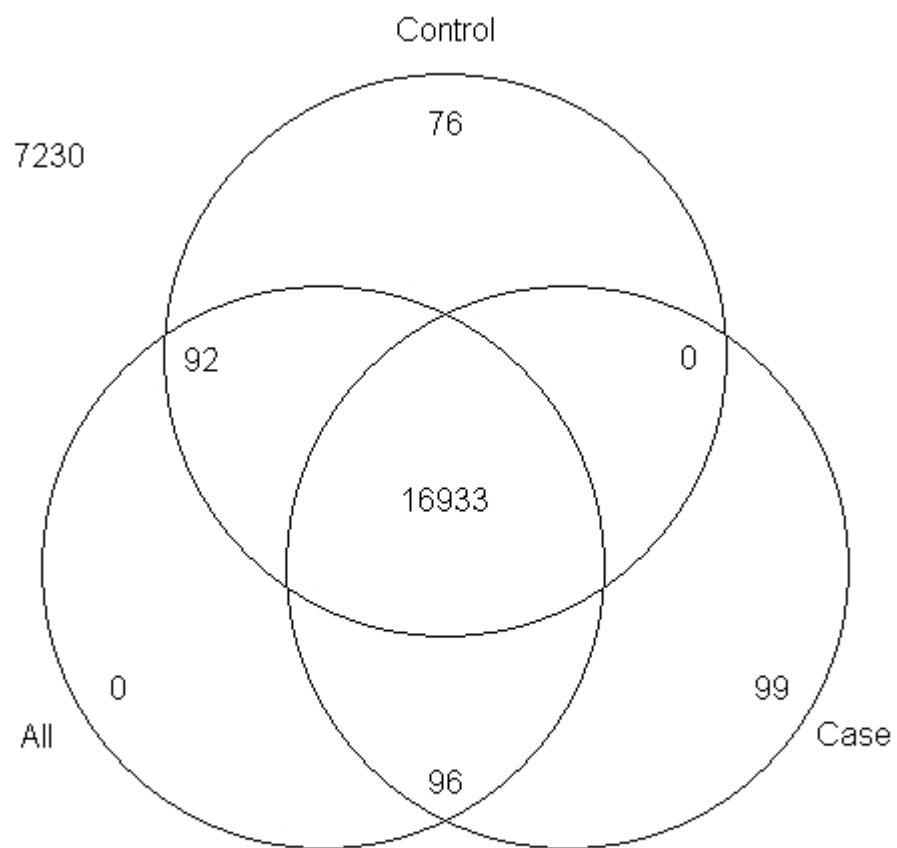

Supplement: Figure S7 — Venn diagram of detectable cerebellar probes. Venn diagram of cerebellar probes detectable in ≥75% of subjects in the AD (AD), non–AD (CON) and combined (All) analyses. Notably, 13,349 probes were detectable in all 374 subjects. (PDF) [file pgen.1002707.s008.pdf]

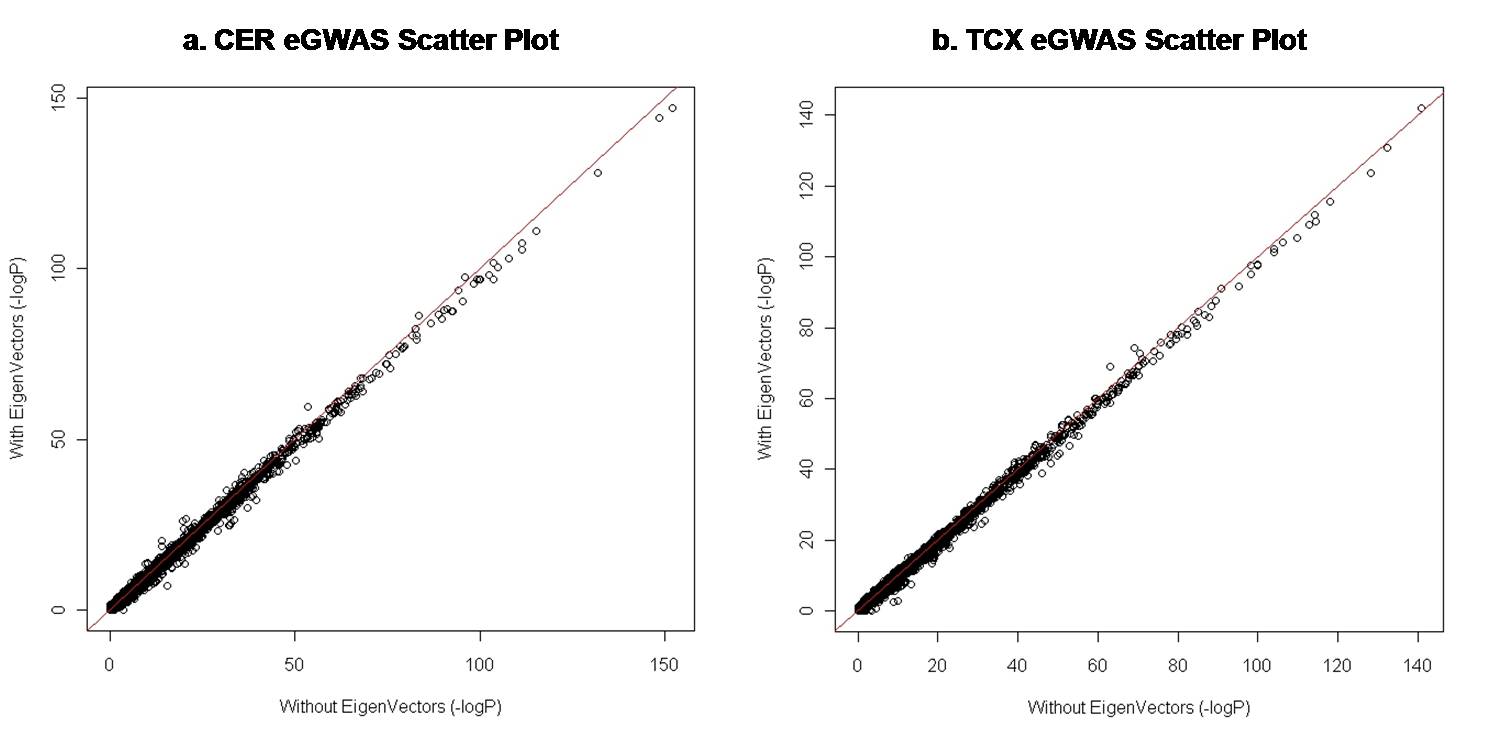

Supplement: Figure S8 — Scatterplots of −log10 p values for eGWAS associations with and without inclusion of eigenvectors. Transformed P-values of a) Cerebellar and b) Temporal Cortex eGWAS cisSNP/transcript associations from models including (y-axis) and excluding (x-axis) the top 10 eigenvectors are plotted. A linear line demonstrating the null hypothesis of no deviation of the results between the two datasets is also shown. The results are displayed for those SNPs with a Hardy-Weinberg P-value>1.0E-07 and a probe detection threshold >75%. (JPG) [file pgen.1002707.s009.jpg]
