# Supplementary material for: Brain Expression Genome-Wide Association Study (eGWAS) Identifies Human Disease-Associated Variants
Source: PLoS Genet. 2012 Jun 7;8(6):e1002707. doi: 10.1371/journal.pgen.1002707 (PMC3369937; doi:10.1371/journal.pgen.1002707)
Supplement: Text S1 — Supplementary Results, Methods and References. (DOC) [file pgen.1002707.s010.doc]

**Brain Expression Genome-Wide Association Study (eGWAS) Identifies Human Disease-Associated Variants**

Fanggeng Zou^1,*^, High Seng Chai^2,*^, Curtis S. Younkin^1,*^, Mariet Allen^1,*^, Julia Crook^3^, V. Shane Pankratz^2^, Minerva M. Carrasquillo^1^, Christopher N. Rowley^1^, Asha A. Nair^2^, Sumit Middha^2^, Sooraj Maharjan^2^, Thuy Nguyen^1^, Li Ma^1^, Kimberly G. Malphrus^1^, Ryan Palusak^1^, Sarah Lincoln^1^, Gina Bisceglio^1^, Constantin Georgescu^1^, Naomi Kouri^1^, Christopher P. Kolbert^4^, Jin Jen^4^, Jonathan L. Haines, PhD^5^, Richard Mayeux, MD^6^, Margaret A. Pericak-Vance, PhD^7^, Lindsay A. Farrer, PhD^8^, Gerard D. Schellenberg, PhD^9^, Alzheimer’s Disease Genetics Consortium@, Ronald C. Petersen^10^, Neill R. Graff-Radford^11^, Dennis W. Dickson^1^, Steven G. Younkin^1^, Nilüfer Ertekin-Taner^1,11,#^.

**Supplementary Results:**

**Transcript level detections:**

Probes that were detectable in >75% of the samples were retained for eGWAS analysis. There were 17,128, 17,101 and 17,121 such probes in the cerebellar AD, non-AD and combined eGWAS. There were 17,284, 16,854 and 17,069 detectable probes in the temporal cortex AD, non-AD and combined eGWAS. Thus, nearly 70% of the probes were detectable in each analyses. Notably, 13,349 probes (54%) detectable in 100% of all 374 cerebellar samples.

**Cerebellar eGWAS *cis*SNP/transcript associations:**

We tested association of *cis*SNPs residing within or ± 100,000 bp flanking genes and their transcript levels for the AD, non-AD and combined datasets. We used three different statistical corrections for multiple testing: the conservative Bonferroni correction (p_Bonf_), FDR-based q values corrected for genomic inflation (q) and 10,000 permutations using the Westfall-Young approach (p_perm-WY_). After Bonferroni correction, there were 2,308 significant *cis*SNP/transcript associations (572 unique genes) in the AD, 1,836 (479 unique genes) in the non-AD and 4,870 (1,013 unique genes) in the combined datasets. There were 5,271 significant *cis*SNP/transcript associations (1,156 unique genes) at q<0.05 in the AD, 4,450 (1,022 unique genes) in the non-AD and 10,281 (1,875 unique genes) in the combined datasets. After 10,000 permutations in the combined dataset, there were 4569 (952 unique genes) *cis*SNP/transcript associations significant at p_perm-WY_ <0.05.

Of the 5,271 significant *cis*SNP/transcript results with q<0.05 in the AD group, 5,179 were tested in the non-ADs (>98%). Similarly, 4,407 of 4,450 significant *cis*SNP/transcript associations from the non-ADs were tested in the ADs (>99%).

There were 2,980 *cis*SNP/transcript associations with q<0.05 in both the AD and non-AD groups (Table 1, Table S3 in Dataset S1, Figure S2). Thus, 58% of significant AD associations are also significant in the non-AD group. This is 68% for the AD group. All 2,980 top associations achieved genome-wide significance with q < 0.05 and p_Bonf_ < 0.05 in the combined (ADs+non-ADs) analysis. All, but three had p_perm-WY_ < 0.05 in the combined analysis.

The subjects of our eGWAS were part of the published Mayo AD GWAS, which was assessed for population stratification using EIGENSTRAT, following which outliers were removed. We, nonetheless, repeated the eGWAS, including the top ten axes of variation generated by EIGENSTRAT as covariates. The results for both the cerebellar and temporal cortex eGWAS p values of association in the analyses including and excluding the eigenvectors were remarkably similar, although they tended to be larger for the analyses including the EIGENSTRAT axes (Figure S8). This did not significantly affect the order, the betas or the p-values of association for the top 2,980 cerebellar eGWAS *cis*SNP/transcript results (Table S27 in Dataset S1), which showed significant correlations in the Spearman-Rank analyses of findings between the eigenvectors-excluded and -included analyses (correlation coefficient~99% and p<0.0001 for all three comparisons).

**Temporal cortex eGWAS *cis*SNP/transcript associations and cerebellar validation:**

Although we used the cerebellar eGWAS as the discovery and temporal cortex as validation in our main analyses; we also utilized temporal cortex as the discovery set and cerebellum as the validation set in order to determine the influence of brain region in the identification of significant *cis*SNPs,. In these analyses, we used HapMap2-imputed genotypes for the eGWAS to allow downstream comparisons with the “Catalog of Published GWAS” . In the temporal cortex eGWAS, there were 68,166 significant *cis*SNP/transcript associations with Q<0.05, 63,844 of which could be tested in the cerebellar eGWAS. Of those, 27,991 were also significant in the cerebellar eGWAS after Bonferroni corrections (24,711 unique *cis*SNPs, 847 unique probes, 782 unique genes) (Table S7 in Dataset S1). When cerebellar eGWAS was used as discovery, there were 77,126 significant *cis*SNP/transcript associations, 70,137 of which could be tested in the temporal cortex eGWAS. Of these, 22,823 were also significant in the temporal cortex after Bonferroni corrections (24,408 unique cisSNPs, 834 unique probes, 767 unique genes) (Table S8 in Dataset S1). There was substantial overlap between the *cis*SNP/transcript associations that were identified in the temporal cortex and validated in the cerebellum, and those identified in the cerebellum and validated in the temporal cortex (660 genes common to both analyses, i.e. 84% of the temporal cortex identified-cerebellar validated and 86% of the cerebellum identified-temporal cortex validated set).

**Variance of human cerebellar gene expression:**

We also calculated in the combined dataset, the total (Raw_variance) and technical variance (R^2^_technical_) of expression for 15,283 probes with at least one *cis*SNP, as well as the adjusted proportion of variation due to biological covariates (adjR2_covariates_) and the “best” *cis*SNP (adjR2_best-SNP_). Of all the 15,283 probes assessed, there were 56 (0.4%), 285 (2%) and 1328 (9%) probes with adjR^2^_best-SNP_ value ≥50%, 20-50% and 5-20%, respectively. Additionally, a substantial amount of expression variation is attributed to technical effects (median: 43%, range: 1-94%).

**Enrichment of brain *cis*SNPs amongst human disease-associated SNPs:**

Using HapMap2 imputed genotypes, no transcript detection level restrictions, p value threshold<1.0E-4 and separate analysis of ADs and non-ADs, we identified 307-383 *cis*SNP/transcript associations (201-241 unique *cis*SNPs) which corresponded to 126-141 unique human disease/trait associations (Tables S12-S15 in Dataset S1). There were 106 human diseases/traits which associated with *cis*SNPs identified in the cerebellar eGWAS of both ADs and non-ADs; and 109 in the temporal cortex eGWAS. Although 17-35 diseases/traits were unique to each eGWAS region and diagnostic group, comparisons with the combined AD+non-AD results revealed that almost all of these could be captured in the larger combined group. This is either because the *cis*SNP/transcript associations are sufficiently strong to be detected in a larger dataset, even if unique to only about half of the group; or because the same *cis*SNPs also influence transcripts similarly in the other diagnostic group, but not strong enough to achieve the p value cutoff.

**Comparison of cerebellar eGWAS results with other published eGWAS:**

Comparison of the top 2,980 *cis*SNP/transcript associations (2,596 unique SNPs, 746 unique transcripts and 686 unique genes) from our cerebellar eGWAS with a liver eGWAS[^1^](#_ENREF_1) on 427 unrelated subjects revealed 62 common *cis* associations (52 unique SNPs, 56 unique transcripts and 51 unique genes) that are significant in both studies. Thus, despite differences in subjects, tissue source, transcript and SNP platforms, ~7.5% of the top cerebellar transcripts and ~4% of the liver transcripts[^1^](#_ENREF_1) with significant *cis*SNP associations were common. However, only 15 significant *cis*SNP/transcript associations (9 unique SNPs, 11 unique transcripts and 7 unique genes) were common with a brain eGWAS[^2^](#_ENREF_2) on normal cortical tissue from 193 subjects, which constitutes ~1.5% of our and 11% of the normal brain eGWAS top *cis* associations. In a follow-up brain eGWAS[^3^](#_ENREF_3) of 176 ADs and 188 pathologically normal controls, 30 significant *cis*SNP/transcript associations (21 unique SNPs, 19 unique transcripts and 14 unique genes) were common with our top *cis* results. Hence, 2.5% of our and 6.7% (19 of 282) of the second brain eGWAS top *cis*SNP/transcript associations were common. All common associations between this second brain eGWAS and ours were with their “noninteraction” set, where *cis*SNPs did not show significant interactions with LOAD. Nevertheless, the common transcripts included *MAPT*, and *GSTO2*, previously implicated in AD risk.

To ensure that the differences in the common significant *cis*SNP associations between these three eGWAS[^1-3^](#_ENREF_1) and our study did not arise from the differences in reported levels of significance, we restricted the comparisons to those associations that were significant at p≤1.42x10^-7^, largest p value from the most stringent published tables. There were still more common associations with the liver eGWAS[^1^](#_ENREF_1) (n=42, unique transcripts= 40), than the “normal” brain eGWAS[^2^](#_ENREF_2) (n=13, unique transcripts = 9) and the follow-up combined AD and control brain eGWAS[^3^](#_ENREF_3) (n=30, unique transcripts=19).

The three published eGWAS[^1-3^](#_ENREF_1), assessed in these comparisons had different expression and genotyping microarray platforms than ours, which may reduce the likelihood of overlaps. Although, it is not possible to resolve the differences with the microarray platforms, we attempted to minimize any false negative results due to genotyping platform differences, by repeating the comparisons using our eGWAS results obtained with the HapMap2-imputed genotypes (>2M SNPs). In our repeat eGWAS, we also applied less stringent criteria, such as no restrictions for transcript detection rates and a p value threshold of <1.0E-4. Comparison of the three published eGWAS results, to these new results revealed 346 common transcripts with the liver eGWAS[^1^](#_ENREF_1) (26% of reported liver transcripts with significant *cis*SNPs); 32 with the “normal” brain eGWAS[^2^](#_ENREF_2) (32%), and 69 with the follow-up brain eGWAS[^3^](#_ENREF_3) (24%). Despite the differences in the threshold of reporting significant results, data QC, expression and genotyping arrays, this overlap is similar to or better than that seen in other eGWAS comparisons[^1^](#_ENREF_1),[^2^](#_ENREF_2).

**Comparison of cerebellar eGWAS results with other published complex disease and trait GWAS:**

Of the 180,669 *cis*SNPs tested in both ADs and controls in our cerebellar eGWAS, 1,277 existed in the disease/trait GWAS catalog. Of the top 2,596 *cis*SNPs, 47 had disease/trait associations. Based on 1 million simulations adjusting for minor allele frequencies of the *cis*SNPs[^9^](#_ENREF_9), we expect a mean of 19.49 significant *cis*SNPs that were also disease/trait-associated. Since there are 47 such *cis*SNPs, we conclude that there is 2.4-fold enrichment of top cerebellar eGWAS *cis*SNPs within the disease/trait GWAS catalog. All 47 *cis*SNP/transcript associations were highly significant even after stringent Bonferroni corrections. These 47 *cis*SNPs had 60 disease/trait associations overall with p<8.0x10^-6^. Under the stringent assumption that each of the 2,596 top *cis*SNPs were tested against each of the 433 unique diseases/traits in the GWAS catalog, the disease/trait associations should have p <3.87x10^-8^ to achieve study-wide significance. Forty of the 60 disease/trait associations were significant with p <3.87x10^-8^ (27 unique SNPs).

It should be noted that the simulations do not adjust for correlations between probes or SNPs. Nevertheless, it is unlikely that such correlations are responsible for the highly significant p values of enrichment obtained in our analyses. Furthermore, the disease/trait associations pertain to 36 unique conditions and 41 unique transcripts, arguing against correlation-related effects contributing to our significant enrichment results.

**Cerebellar and temporal cortex *cis*SNP/transcript associations in the Progressive Supranuclear Palsy (PSP) subset:**

Most of the subjects among the non-ADs consist of those with PSP pathology (Table S2 in Dataset S1). We, therefore, repeated the top 2,980 cerebellar *cis*SNP/transcript associations for the 98 pathology-confirmed PSP subjects in our cerebellar eGWAS and determined that 673 of these top associations (239 unique probes and 226 unique genes) were significant even after a stringent Bonferroni correction for 443,132 tests conducted in this relatively small subset (Table S19 in Dataset S1). After a study-wide correction for 2,980 tests, 1,361 (402 unique probes and 373 unique genes) of the top associations were significant in the PSP subjects. The direction and magnitude of the top *cis*SNP/transcript associations that are also significant in the PSP subset revealed that 2,097 out of 2,980 associations had regression coefficients within ≤20% of each other. We determined that 2,636 of the top associations were detectable in the temporal cortex of the PSP subjects, of which 867 achieved study-wide significance (Table S20 in Dataset S1) and 493 reached genome-wide significance. There were 656 top *cis*SNP/transcript associations that achieved study-wide significance in the cerebellar and temporal cortex analyses for the PSP subjects (189 unique probes and 181 unique genes). The regression coefficients for 499 of these top associations that are also significant in two brain regions of PSP subjects were within ≤20% of each other between the cerebellar and temporal cortex PSP associations.

**Combined analysis of eGWAS and ADGC GWAS Results:**

There were 665 significant cerebellar transcript associations (380 unique SNPs, 15 unique genes) with *cis*SNPs that were also suggestive AD risk SNPs in the ADGC GWAS (Tables S17-S18 in Dataset S1). There were 460 such temporal cortex associations (432 unique SNPs, 15 unique genes). Genes that have significant brain *cis*SNPs and suggestive AD risk association include *MAPT* and *LRR37A4*, previously implicated in PSP[^10^](#_ENREF_10) and PD GWAS[^11^](#_ENREF_11) and *ABCA7*, recently identified in LOAD GWAS. Significant *MAPT cis*SNPs have AD risk association meta-analysis p values (p_meta_) of 8.82x10^-4^-1.53x10^-5^ (Tables S17-S18 in Dataset S1). *MME*, encoding neprilysin, which is an amyloid β degrading protease had significant brain expression level (p_Cer_=7.46x10^-6^) and suggestive AD risk association (p_meta_=3x10^-4^), as did *UBE2L3* (p_Cer_=7.99x10^-5^-3.69x10^-5^; p_meta_=5.11x10^-5^-1.07x10^-5^), encoding ubiquitin-conjugating enzyme E2 L3, which interacts with the familial PD gene product parkin in protein degradation[^16^](#_ENREF_16). Given the converging statistical and biological evidence, these genes merit further investigations as potential genetic modifiers for risk of AD, though the gene expression associations for *MME* and *UBE2L3* require additional confirmation given the existence of a SNP within the expression probe for these two genes.

**Variants within Probes:**

There is concern that presence of sequence variants within genomic regions targeted by the expression probes could lead to artifactual variance in the expression levels. Consequently, the variants within probe regions or those with which they are in linkage disequilibrium could have spurious and non-biological associations with levels of transcript detected by that probe. Given this concern, we annotated all of the probes by comparing their positions according to NCBI Ref Seq, Build 36.3 to those of all variants within dbSNP131 and identified the list of probes which have ≥1 variants within their sequence. Among the 443,784 cerebellar eGWAS associations (17,121 unique probes) tested in the combined group, 42,607 (10%) pertained to probes with ≥1 variants within their sequence. There were 1,441 unique probes that harbor a polymorphism (8%). The top 2,980 cerebellar *cis*SNP/transcript associations (746 unique probes) had 572 results from 124 unique probes with ≥1 polymorphisms in them. Thus, 19% of the top associations (17% of unique probes) come from probes with variants.

While the majority of the top associations arise from probes that do not have an annotated variant within their sequence, there was an excess of probes with variants among the top hits compared with all tested probes.

*MAPT* expression levels were found to have substantial genetic influence in the brain by others[^2^](#_ENREF_2) and also in our study. However, the *MAPT* DASL probe that yields the strongest associations (ILMN_1710903) also harbors two annotated SNPs, rs67759530 and rs66561280, within its sequence. We genotyped these SNPs in our eGWAS subjects and determined that both are in complete LD with the top eSNP rs1981997 and the *MAPT* H1-haplotype tagging SNP rs1052553. While this does not automatically indicate that the eGWAS *MAPT* expression/rs1981997 association is an artifact, it nevertheless raises concern. We determined that a second *MAPT* DASL probe (ILMN_2298727) also yielded significant eGWAS results, though the strength of association for the best eSNP (rs8070723, p=3.4x10^-7^ in ALL) was less than that for ILMN_1710903 (rs1981997, p=4.2x10^-71^). Although ILMN_2298727 also has a SNP in its sequence, rs73314997, it is essentially monomorphic in the eGWAS subjects (only one heterozygote and two missing genotypes). After re-running the eGWAS *MAPT* associations for ILMN_2298727 expression levels on subjects who are major homozygotes for the probe variant, rs73314997, the significant associations persisted, as expected (data not shown). Importantly, the *MAPT* H1 haplotype-tagging SNP rs1052553 was associated with higher *MAPT* ILMN_2298727 probe levels (p=1.9x10^-6^ in ALL) as well as the H1c haplotype-tagging SNP rs242557 (p=0.01 in ALL). In summary, these results suggest that *MAPT*-expression/eSNP associations in our cerebellar eGWAS are unlikely to be due to artifact and are in agreement with previous findings.

**Trans associations:**

Although not the main focus of this study, we also assessed *trans*-associations with SNPs outside of genes and their 100 kb flanking regions. In the combined ADs+non-ADs analysis, there were ~5.45x10^9^ *trans*-associations tested (17,121 unique probes x 318,237 unique SNPs – 443,784 *cis*-associations), requiring an uncorrected p value < 9.18x10^-12^ to achieve a Bonferroni-corrected p_Bonf_ < 0.05. There were 1,288 such *trans*-associations in the combined analysis. Upon further investigation, we noted that 121 of these associations arose from probes that did not have annotated chromosome locations. Of the remaining 1,167 significant *trans*-associations, all but two were from SNP/transcript pairs that reside on the same chromosome. The small number of significant *trans*-associations suggest that *trans*SNPs are likely to have smaller effect sizes than *cis*SNPs, as consistent with prior eQTL studies.

**Supplementary Methods:**

**Gene expression measurements:**

Transcript levels were measured using the Whole Genome DASL assay (WG-DASL, c-**D**NA mediated **A**nnealing, extension, **S**election, **L**igation assay, Illumina, San Diego, CA), which is designed specifically for partially degraded RNA obtained from fresh frozen or formalin-fixed paraffin-embedded tissues. WG-DASL assay content has 24,526 probes against 18,401 genes and is annotated according to the NCBI Ref Seq, Build 36.2. Extracted RNA was labeled and hybridized according to manufacturer’s instruction for WG-DASL assays at the Mayo Clinic, Gene Expression Core. Briefly, 100 ng of total RNA was reverse transcribed with biotinylated oligo(dT) and random nonamer primers. The resulting cDNA was annealed to chimeric query oligonucleotides, which contain a gene-specific region and a universal primer sequence for PCR amplification, and then bound to streptavidin-conjugated paramagnetic particles. The gene-specific oligonucleotides were extended by second-strand cDNA synthesis and then ligated. Subsequently, the products were sequestered by magnetic separation, washed to remove unbound molecules, and then amplified by PCR with fluorophore-labeled universal primers. The resulting PCR products were purified, applied to HumanRef-8 v3 beadchips (Illumina), and then hybridized for 16 h at 58º C. The beadchips were washed and then scanned in a BeadArray Reader using BeadScan v3 software (Illumina). Quality control (QC) parameters were determined to be within normal ranges before proceeding to the final data reduction.

There were 4 AD and 4 non-AD temporal cortex samples that were measured in 5 replicates. For the cerebellar gene expression measurements, 10 AD samples were run in replicate fashion across six plates and 5 non-AD samples were run as replicates across five plates. Universal human RNA (UHR) samples were also run on each PCR plate as part of QC.

**Genotype data:**

Genomic DNA was obtained from the cerebellum of subjects in the AUT series by Wizard® Genomic DNA Purification Kit (Promega Corp., Madison, WI) and subjected to whole genome amplification using the Illustra GenomiPhi V2 DNA Amplification Kit (GE Healthcare Bio-Sciences Corp., Piscataway, NJ), as described[^21^](#_ENREF_21).

The LOAD GWAS QC methods were previously published[^21^](#_ENREF_21). Briefly, using PLINK[^22^](#_ENREF_22), subjects with genotyping call rates of <90%, duplicate genotyping and/or sex-mismatch between recorded and deduced sex were eliminated from analyses. All SNPs with genotyping call rates <90%, minor allele frequencies<0.01, and/or Hardy-Weinberg p values<0.01 were eliminated from analyses.

**Statistical Methods for eGWAS:**

PLINK analyses were run in parallel on a Beowulf cluster of servers. Perl scripts were used to extract results on *cis*-SNPs and the results are maintained on an SQL server. We annotated all of the probes by comparing their positions according to NCBI Ref Seq, Build 36.3 to those of all SNPs within dbSNP131 and identified the list of probes which have ≥1 variants within their sequence. We added this information to all of the results in our database and this manuscript.

Multiple testing corrections were done with the Bonferroni, q value and permutation methods. The numbers of tests for the AD, non-AD and combined cerebellar eGWAS analyses, which were used in Bonferroni corrections, were 444,372; 443,171 and 443,784, respectively.

**Q-Q Plots:**

Q-Q plots of p-values of association in the combined AD and non-AD cerebellar eGWAS analysis (Figures S1a-b), suggested an inflation in the number of significant p-values. After controlling for this genomic inflation, we continued to observe an excess of significant associations (Figure S1-d). While this could, in part, be due to correlations between tested transcripts and/or linkage disequilibrium between the SNPs that are not completely accounted for even after controlling for genomic inflation, they are most likely to be indicative of true positive *cis*SNP/transcript associations. Using the inflation-corrected p values, we obtained FDR-based q values (q)[^23^](#_ENREF_23). The same approach was applied to obtain q values for the cerebellar and temporal cortex associations which utilized the Hap Map 2 imputed >2 million SNPs, that were utilized subsequently for joint analysis with the ADGC GWAS (Figures S6a-d).

**Variance of Gene Expression:**

To estimate the variance in human cerebellar gene expression, we first determined between-subject variance, as a percentage of the total variance in probe expression (intraclass coefficients=ICC) for 15 samples measured in replicate on 5-6 different plates and 2-3 different days. 10 AD samples on 6 plates hybridized on 3 separate days and 5 non-AD samples on 5 plates hybridized on 2 separate days. To determine the relative amount of gene expression variance between the different unique samples as a percentage of the total variance, intraclass correlation coefficients (ICC) were calculated[^24^](#_ENREF_24), where ICC is defined as:

[Variance between unique samples (i.e. across samples variation)]/[Total variance];

where [Total variance] is:

[Variance between unique samples + Variance of the residuals (i.e. within samples)].

The variances are obtained from the random effects model after adjustment for the plate effect.

Using multivariable linear regression models, we then calculated the proportion of variance in cerebellar gene expression levels that were explained by technical effects, biological covariates and the “best” *cis*SNP for each probe. These analyses were carried out on the combined dataset consisting of expression measurements from 374 subjects and 15,283 probes with at least one *cis*SNP. The proportion of variance in gene expression that is due to technical effects (R^2^_technical_) is defined as the coefficient of determination of the linear regression model adjusted for technical variables only (i.e. plates and RIN). Added proportion of explained variation due to biological covariates (addR^2^_covariates_) represents the increase in the R^2^ value after adding the non-technical variables (i.e. age, sex and ApoE4 dose) to the linear regression model. To determine the variance due to the “best” *cis*SNP, the supplementary increase in R^2^ was calculated one *cis*SNP at a time. The *cis*SNP which led to the greatest increase in R^2^, after its inclusion in the “technical+covariates” model was the “best” *cis*SNP for that probe with added explained variation denoted by addR^2^_best-SNP_. For comparison of expression variation while controlling for technical variance, which can be large, we used adjusted R^2^, such that “adjR^2^_covariates_” = (addR^2^_covariates_)/(1- R^2^_technical_) for biological effects and “adjR^2^_best-SNP_” = (addR^2^_best-SNP_)/(1- R^2^_technical_) for the “best” *cis*SNP.

**Comparison of cerebellar eGWAS results with other published eGWAS:**

To compare the results of our human cerebellar eGWAS to those from other studies, we obtained the results from a human liver[^1^](#_ENREF_1) and two human brain eGWAS. The liver eGWAS[^1^](#_ENREF_1) was conducted on 427 unrelated subjects measured for levels of 39,280 transcripts (34,266 genes), using a custom Agilent microarray and 782,476 SNPs using Affymetrix 500K and Illumina 650Y platforms. In this study, at Bonferroni adjusted p <0.05, there were 1,350 expression traits (1,273 genes, 3.7%) and at FDR <10%, 3,210 traits (3,043 genes; 8.8%) which had ≥1 significant *cis*SNP (within gene±1 Mb from transcription start-stop site). Trans eQTLs significant at Bonferroni p<0.05 were 242 traits (236 genes), and at FDR<10% were 491 traits (474 genes). The eSNPs explained 2-90% of variation in expression levels, in this study. The significant *cis*SNP results from Supplementary Table S2 of this study were linked to our list of 2,980 significant *cis*SNPs (Table S3 in Dataset S1) by both gene and SNP names.

The first brain eGWAS[^2^](#_ENREF_2) assessed neuropathologically “normal” cerebral cortical tissue from 193 subjects for 14,078 transcripts using Illumina Human Refseq-8 Expression BeadChip and 366,140 SNPs on the Affymetrix 500K platform. They determined that 58% of the transcriptome had expression in ≥5% of control brains. Of these, 21% had significant associations with a *cis* (within gene±1 Mb from transcription start-stop site) or *trans*SNP. This study identified 433 significant *cis*SNPs (99 transcripts) and 16,701 *trans*SNPs (2,876 transcripts). The significant *cis*SNP results from Supplementary Tables 2 and 3 of this study were linked to our list of 2,980 significant *cis*SNPs by both gene and SNP names.

In the follow-up brain eGWAS, 176 brain samples with AD neuropathology were assessed, and a joint evaluation with 188 control brains from the prior study[^2^](#_ENREF_2) was conducted. The analyses were restricted to 8,650 transcripts out of 24,357 (35.5%) measured with Illumina Human Refseq-8 Expression BeadChips, which were detected in >90% of the cases and controls. Again, the Affymetrix 500K platform (380,157 SNPs) was used. Similar to control brains, 58% of the transcriptome was detectable in ≥5% of AD brains. There were 1,829 significant *cis*SNPs (within gene±1 Mb from transcription start-stop site) in their combined sample and 656 significant *trans*-eSNPs. Twenty-seven percent of all eQTLs were found to have significant interaction term with diagnosis. The significant *cis*SNP results from Supplementary Tables S5 and S6 of this study were linked to our list of 2,980 significant *cis*SNPs by both gene and SNP names.

**Alzheimer’s Disease Genetics Consortium (ADGC) Meta-Analyses:**

ADGC stage 1 cohorts are Adult Changes in Thought (ACT)/ Electronic Medical Records and Genetics (eMERGE) study, the National Institute on Aging (NIA) Alzheimer Disease Centers (ADCs), the Alzheimer Disease Neuroimaging Initiative (ADNI) Study, the Multi-Site Collaborative Study for Genotype-Phenotype Associations in Alzheimers Disease (GenADA) Study, the University of Miami/Vanderbilt University/Mt. Sinai School of Medicine (UM/VU/MSSM), the MIRAGE Study, Oregon Health and Science University (OHSU), the NIA-LOAD Study, and the Translational Genomics Research Institute series 2 (TGEN2). ADGC stage 2 is comprised of the following cohorts: Mayo Clinic, the Rush University Religious Orders Study/Memory and Aging Project (ROSMAP), the University of Pittsburgh (UP), and Washington University (WU). The detailed descriptions of these cohorts are provided elsewhere.

Each cohort was evaluated individually for identification and removal of outliers prior to estimation of population substructure and principal components using EIGENSTRAT[^26^](#_ENREF_26), as described[^12^](#_ENREF_12). The genotyped and imputed SNPs, which passed QC, were tested for AD risk association for each cohort by a logistic regression approach assuming an additive model where the genotyped SNPs were coded by the number of minor alleles (0, 1, 2) and the imputed SNPs by the expected dosage of the tested allele from MACH[^27^](#_ENREF_27). The results reported here are based on the extended model, which adjusts for age (at onset for cases and at exam for controls), sex and APOE ε4 dosage, in addition to the principal components from EIGENSTRAT[^26^](#_ENREF_26).

The meta-analyses results were generated using the inverse variance method implemented in the software package METAL[^28^](#_ENREF_28). The meta-analysis P value was estimated by the summarized test statistic after applying a genomic control within each individual study. The SNP/transcript and SNP/AD risk associations presented here (Tables S17-18 in Dataset S1) depict the effects of same tested allele to simplify comparison of brain gene expression and AD risk effects for each SNP.

**Combined analysis of eGWAS and ADGC GWAS Results:**

To ensure a uniform analysis of variants, a common set of >2 million SNPs were imputed by the ADGC, as described[^12^](#_ENREF_12). We assessed meta-analyses results from combined Stage 1 and 2 cohorts for 2,273,789 SNPs from ADGC. Imputations were also done for the subjects in our eGWAS. To maximize the SNP comparisons between the ADGC GWAS and our brain eGWAS, we repeated the eGWAS analyses using 2,543,887 SNPs and the same analytic models as described above. There were 3,150,482 cerebellar and 3,169,701 temporal cortex *cis*SNP/transcript results. Inflation-corrected Q-Q plots clearly show an excess of significant brain expression associations (**Figures S6a-d**). There were 77,126 cerebellar (63,652 unique SNPs, 2,338 unique genes) and 68,172 temporal cortex (57,922 unique SNPs and 2,201 unique genes) *cis*SNP/transcript associations significant at q<0.05 (p_cerebellum_=1.1x10^-4^-1.75x10^-157^; _pTCx_=1.4x10^-4^-2.01x10^-136^). We linked these significant *cis*SNPs to the suggestive meta-analysis results from ADGC (p<10^-3^, 3,796 SNPs).

**Supplementary References:**

1. Schadt EE, Molony C, Chudin E, et al. Mapping the genetic architecture of gene expression in human liver. PLoS Biol 2008;6:e107.

2. Myers AJ, Gibbs JR, Webster JA, et al. A survey of genetic human cortical gene expression. Nat Genet 2007;39:1494-1499.

3. Webster JA, Gibbs JR, Clarke J, et al. Genetic control of human brain transcript expression in Alzheimer disease. Am J Hum Genet 2009;84:445-458.

4. Caffrey TM, Joachim C, Paracchini S, Esiri MM, Wade-Martins R. Haplotype-specific expression of exon 10 at the human MAPT locus. Hum Mol Genet 2006;15:3529-3537.

5. Caffrey TM, Joachim C, Wade-Martins R. Haplotype-specific expression of the N-terminal exons 2 and 3 at the human MAPT locus. Neurobiol Aging 2008;29:1923-1929.

6. Myers AJ, Pittman AM, Zhao AS, et al. The MAPT H1c risk haplotype is associated with increased expression of tau and especially of 4 repeat containing transcripts. Neurobiol Dis 2007;25:561-570.

7. Li YJ, Oliveira SA, Xu P, et al. Glutathione S-transferase omega-1 modifiesage-at-onset of Alzheimer disease and Parkinson disease. Hum Mol Genet 2003;12:3259-3267.

8. Li YJ, Scott WK, Zhang L, et al. Revealing the role of glutathione S-transferase omega in age-at-onset of Alzheimer and Parkinson diseases. Neurobiol Aging 2006;27:1087-1093.

9. Nicolae DL, Gamazon E, Zhang W, Duan S, Dolan ME, Cox NJ. Trait-associated SNPs are more likely to be eQTLs: annotation to enhance discovery from GWAS. PLoS Genet 2010;6:e1000888.

10. Hoglinger GU, Melhem NM, Dickson DW, et al. Identification of common variants influencing risk of the tauopathy progressive supranuclear palsy. Nat Genet 2011;43:699-705.

11. Simon-Sanchez J, Schulte C, Bras JM, et al. Genome-wide association study reveals genetic risk underlying Parkinson's disease. Nat Genet 2009;41:1308-1312.

12. Naj AC, Jun G, Beecham GW, et al. Common variants at MS4A4/MS4A6E, CD2AP, CD33 and EPHA1 are associated with late-onset Alzheimer's disease. Nat Genet 2011;43:436-441.

13. Hollingworth P, Harold D, Sims R, et al. Common variants at ABCA7, MS4A6A/MS4A4E, EPHA1, CD33 and CD2AP are associated with Alzheimer's disease. Nat Genet 2011;43:429-435.

14. Iwata N, Tsubuki S, Takaki Y, et al. Metabolic regulation of brain Abeta by neprilysin. Science 2001;292:1550-1552.

15. Leissring MA, Farris W, Chang AY, et al. Enhanced proteolysis of beta-amyloid in APP transgenic mice prevents plaque formation, secondary pathology, and premature death. Neuron 2003;40:1087-1093.

16. Shimura H, Hattori N, Kubo S, et al. Familial Parkinson disease gene product, parkin, is a ubiquitin-protein ligase. Nat Genet 2000;25:302-305.

17. Stranger BE, Forrest MS, Clark AG, et al. Genome-wide associations of gene expression variation in humans. PLoS Genet 2005;1:e78.

18. Doss S, Schadt EE, Drake TA, Lusis AJ. Cis-acting expression quantitative trait loci in mice. Genome Res 2005;15:681-691.

19. Cheung VG, Spielman RS, Ewens KG, Weber TM, Morley M, Burdick JT. Mapping determinants of human gene expression by regional and genome-wide association. Nature 2005;437:1365-1369.

20. Goring HH, Curran JE, Johnson MP, et al. Discovery of expression QTLs using large-scale transcriptional profiling in human lymphocytes. Nat Genet 2007;39:1208-1216.

21. Carrasquillo MM, Zou F, Pankratz VS, et al. Genetic variation in PCDH11X is associated with susceptibility to late-onset Alzheimer's disease. Nat Genet 2009;41:192-198.

22. Purcell S, Neale B, Todd-Brown K, et al. PLINK: a tool set for whole-genome association and population-based linkage analyses. Am J Hum Genet 2007;81:559-575.

23. Storey JD, Tibshirani R. Statistical significance for genomewide studies. Proc Natl Acad Sci U S A 2003;100:9440-9445.

24. Barry WT, Kernagis DN, Dressman HK, et al. Intratumor heterogeneity and precision of microarray-based predictors of breast cancer biology and clinical outcome. J Clin Oncol 2010;28:2198-2206.

25. Jun G, Naj AC, Beecham GW, et al. Meta-analysis Confirms CR1, CLU, and PICALM as Alzheimer Disease Risk Loci and Reveals Interactions With APOE Genotypes. Arch Neurol 2010.

26. Price AL, Patterson NJ, Plenge RM, Weinblatt ME, Shadick NA, Reich D. Principal components analysis corrects for stratification in genome-wide association studies. Nat Genet 2006;38:904-909.

27. Li Y, Willer CJ, Ding J, Scheet P, Abecasis GR. MaCH: using sequence and genotype data to estimate haplotypes and unobserved genotypes. Genet Epidemiol 2010;34:816-834.

28. Willer CJ, Li Y, Abecasis GR. METAL: fast and efficient meta-analysis of genomewide association scans. Bioinformatics 2010;26:2190-2191.
